# Molecular and Physiological Adaptations to Low Temperature in *Thioalkalivibrio* Strains Isolated from Soda Lakes with Different Temperature Regimes

**DOI:** 10.1128/mSystems.01202-20

**Published:** 2021-04-27

**Authors:** Anne-Catherine Ahn, Evelien Jongepier, J. Merijn Schuurmans, W. Irene C. Rijpstra, Jaap S. Sinninghe Damsté, Erwin A. Galinski, Pawel Roman, Dimitry Sorokin, Gerard Muyzer

**Affiliations:** a Microbial Systems Ecology, Department of Freshwater and Marine Ecology, Institute for Biodiversity and Ecosystem Dynamics, University of Amsterdam, Amsterdam, The Netherlands; b Department of Marine Microbiology and Biogeochemistry, NIOZ Royal Netherlands Institute for Sea Research and Utrecht University, Den Burg, The Netherlands; c Department of Earth Sciences, Faculty of Geosciences, Utrecht University, Utrecht, The Netherlands; d Institute of Microbiology and Biotechnology, Rheinische Friedrich-Wilhelms University, Bonn, Germany; e Wetsus, European Centre of Excellence for Sustainable Water Technology, Leeuwarden, The Netherlands; f Winogradsky Institute of Microbiology, Research Centre of Biotechnology, Russian Academy of Sciences, Moscow, Russia; g Department of Biotechnology, Delft University of Technology, Delft, The Netherlands; California State University, Northridge

**Keywords:** *Thioalkalivibrio*, soda lake, system biology, RNA-Seq, membrane lipid composition, glycine betaine

## Abstract

The genus *Thioalkalivibrio* comprises sulfur-oxidizing bacteria thriving in soda lakes at high pH and salinity. Depending on the geographical location and the season, these lakes can strongly vary in temperature. To obtain a comprehensive understanding of the molecular and physiological adaptations to low temperature, we compared the responses of two *Thioalkalivibrio* strains to low (10°C) and high (30°C) temperatures. For this, the strains were grown under controlled conditions in chemostats and analyzed for their gene expression (RNA sequencing [RNA-Seq]), membrane lipid composition, and glycine betaine content. The strain Thioalkalivibrio versutus AL2^T^ originated from a soda lake in southeast Siberia that is exposed to strong seasonal temperature differences, including freezing winters, whereas Thioalkalivibrio nitratis ALJ2 was isolated from an East African Rift Valley soda lake with a constant warm temperature the year round. The strain AL2^T^ grew faster than ALJ2 at 10°C, likely due to its 3-fold-higher concentration of the osmolyte glycine betaine. Moreover, significant changes in the membrane lipid composition were observed for both strains, leading to an increase in their unsaturated fatty acid content via the Fab pathway to avoid membrane stiffness. Genes for the transcriptional and translational machinery, as well as for counteracting cold-induced hampering of nucleotides and proteins, were upregulated. Oxidative stress was reduced by induction of vitamin B_12_ biosynthesis genes, and growth at 10°C provoked downregulation of genes involved in the second half of the sulfur oxidation pathway. Genes for intracellular signal transduction were differentially expressed, and interestingly, AL2^T^ upregulated flagellin expression, whereas ALJ2 downregulated it.

**IMPORTANCE** In addition to their haloalkaline conditions, soda lakes can also harbor a variety of other extreme parameters, to which their microbial communities need to adapt. However, for most of these supplementary stressors, it is not well known yet how haloalkaliphiles adapt and resist. Here, we studied the strategy for adaptation to low temperature in the haloalkaliphilic genus *Thioalkalivibrio* by using two strains isolated from soda lakes with different temperature regimes. Even though the strains showed a strong difference in growth rate at 10°C, they exhibited similar molecular and physiological adaptation responses. We hypothesize that they take advantage of resistance mechanisms against other stressors commonly found in soda lakes, which are therefore maintained in the bacteria living in the absence of low-temperature pressure. A major difference, however, was detected for their glycine betaine content at 10°C, highlighting the power of this osmolyte to also act as a key compound in cryoprotection.

**Author Video:** An author video summary of this article is available.

## INTRODUCTION

Soda lakes are extreme haloalkaline environments with a high microbial diversity despite their harsh conditions (1–3). These conditions are characterized by a pH ranging from 9.5 to 11 and salt concentrations up to saturation (4, 5). These hypersaline alkaline lakes with a large fraction of sodium carbonates are found worldwide in arid and semiarid regions, for example, in the Kulunda Steppe in South Siberia (2, 3), in the Wadi El Natrun in Egypt (6), in the Rift Valley in East Africa (7), in the Great Basin Desert in eastern California and western Nevada (8), and in the Cariboo Plateau in British Columbia (Canada) (9). Soda lakes can exert multiple types of stresses on their microbial community in addition to their haloalkaline conditions. For instance, certain lakes are exposed to fluctuating temperatures throughout the year, whereas others are located in regions with a stable temperature profile. Despite these multiple extreme conditions, various bacteria and archaea and also some eukaryotes can thrive in these lakes (1, 5, 9–11). These communities are actively involved in the biogeochemical cycling of carbon, nitrogen, and sulfur, which makes these ecosystems very productive (12). One of the most abundant bacterial genera found in hypersaline soda lakes is *Thioalkalivibrio* (2, 3).

Members of the genus *Thioalkalivibrio* are haloalkaliphilic chemolithoautotrophic sulfur-oxidizing bacteria grouped in the family *Ectothiorhodospiraceae* within the *Gammaproteobacteria*. The genus consists of more than 100 isolates (13), including 10 described species (14) and 25 *in silico*-defined species (15), which are all well adapted to the haloalkaline conditions of soda lakes (12, 16). These strains have been isolated from soda lakes worldwide, including lakes with different temperature regimes (17–19), raising the questions of how they are adapted to different temperatures in general and to low temperature in particular.

Low temperature profoundly affects the growth and survival of bacteria. To adapt to it, bacteria have developed a number of strategies. For example, the cold-induced rigidification of the cellular membrane is counteracted by increasing the proportion of unsaturated, short-chain, and branched-chain fatty acids (20). To reduce the induced damage inside the cell, compatible compounds, which include the osmolytes glycine betaine and ectoine, are assimilated or produced (21, 22). Moreover, negative supercoiling of DNA (23) and overstabilization of secondary structures in RNA (24, 25) are corrected by helicases (26) and cold shock proteins (27, 28) and protein misfolding (29) by chaperones such as GroEL and GroES (30). Another stressor that accompanies the drop in temperature is the increased formation of reactive oxygen species (ROS) (31) due to higher solubility of oxygen at low temperatures (32). To overcome this threat, antioxidant enzymes such as superoxide dismutase, catalase, and peroxidase are induced (31). Despite all these challenges, bacteria have managed to successfully colonize cold environments (33).

Here, we studied the molecular and physiological adaptation mechanisms of two moderate halophilic *Thioalkalivibrio* strains, Thioalkalivibrio versutus AL2^T^ and Thioalkalivibrio nitratis ALJ2, to low temperature. These strains were chosen because they were isolated from soda lakes with different temperature regimes: *T. versutus* AL2^T^ was isolated from Lake Hadyn in southeastern Siberia, where it is subjected to strong seasonal fluctuations with freezing temperatures in winter (down to –35°C) and warm summers (up to 27°C) (17, 34) (https://worldweather.wmo.int/en/city.html?cityId=1031), while *T. nitratis* ALJ2 originates from Lake Elmenteita in the East African Rift Valley with a constant temperature of ca. 20°C the year round (7, 17) (https://worldweather.wmo.int/en/city.html?cityId=518). The strains were grown under controlled conditions in chemostats at 10°C and 30°C, also referred to here as low and high temperatures, respectively. To compare their responses to low temperature, we studied gene expression by transcriptomics, including strain-specific responses to temperature, and analyzed their adaptation of membrane lipid composition and the production of the cryoprotectant glycine betaine at both 10°C and 30°C.

## RESULTS AND DISCUSSION

For our experiment, we selected two *Thioalkalivibrio* strains from geographical locations with different temperature regimes. Based on their different origins, we expected that these strains would exhibit different responses to low temperature.

Batch cultivation showed that *T. versutus* AL2^T^ grew much faster than *T. nitratis* ALJ2 (μ_max_ of 0.064/h versus 0.024/h, respectively) at 10°C, while this was the opposite at 30°C (μ_max_ of 0.28/h versus 0.43/h) (Table 1). To gain further insights into the mechanisms behind these differences in growth rate, the bacteria were subsequently grown at 10°C and 30°C under controlled conditions in chemostats until steady state. As the growth rate of the bacterial culture is set by the dilution rate and the samples were taken at the steady state of the chemostat cultures, all changes in the gene expression and the membrane lipids originate from the difference in temperature and strain between the reactors and are not influenced by the growth state of the culture, as is the case in batch cultivation. The strength of repeatability of chemostat cultivation is illustrated by the close clustering of the samples from the same condition in the principal-component analysis (PCA) (Fig. S1C). The PCA also shows that the gene expression profiles of both strains were markedly different between the two temperature regimes and strains.

**TABLE 1:** Growth parameters of batch cultures of *T. versutus* AL2^T^ and *T. nitratis* ALJ2 at 10°C and 30°C

Organism and growth temp (°C)	Lag phase (h)	Maximum growth rate (/h)	Stationary phase (10^8^ cells/ml)
*T. versutus* AL2^T^			
10	107.22 ± 6.21	0.064 ± 0.012	1.63 ± 0.041
30	24.63 ± 2.23	0.28 ± 0.064	2.62 ± 0.12
*T. nitratis* ALJ2			
10	306.17 ± 2.63	0.024 ± 0.0012	2.92 ± 0.087
30	27.32 ± 1.85	0.43 ± 0.094	3.41 ± 0.081

10.1128/mSystems.01202-20.1 FIG S1 (A) Schematic representation of the experimental set-up using continuous cultivation in chemostat reactors. (B) Cell counts measured by flow cytometry during chemostat cultivation of *T. versutus* AL2^T^ and *T. nitratis* ALJ2 at 10°C and 30°C. Arrows and dashed lines indicate the periods of continuous cultivation. The error bars depict the standard deviation of the average. A zoom is depicted for *T. versutus* AL2^T^ at 30°C. (C) Principal-component analysis (PCA) based on the expression data of the identified orthologs of *T. versutus* AL2^T^ and *T. nitratis* ALJ2. The symbols represent the projections of samples onto the principal components 1 and 2 based on sleuth-normalized est_counts using the plot_pca utility of the sleuth R package (93). Download FIG S1, EPS file, 2.1 MB. Copyright © 2021 Ahn et al. 2021 Ahn et al. https://creativecommons.org/licenses/by/4.0/ This content is distributed under the terms of the Creative Commons Attribution 4.0 International license.

**FIG 1:**
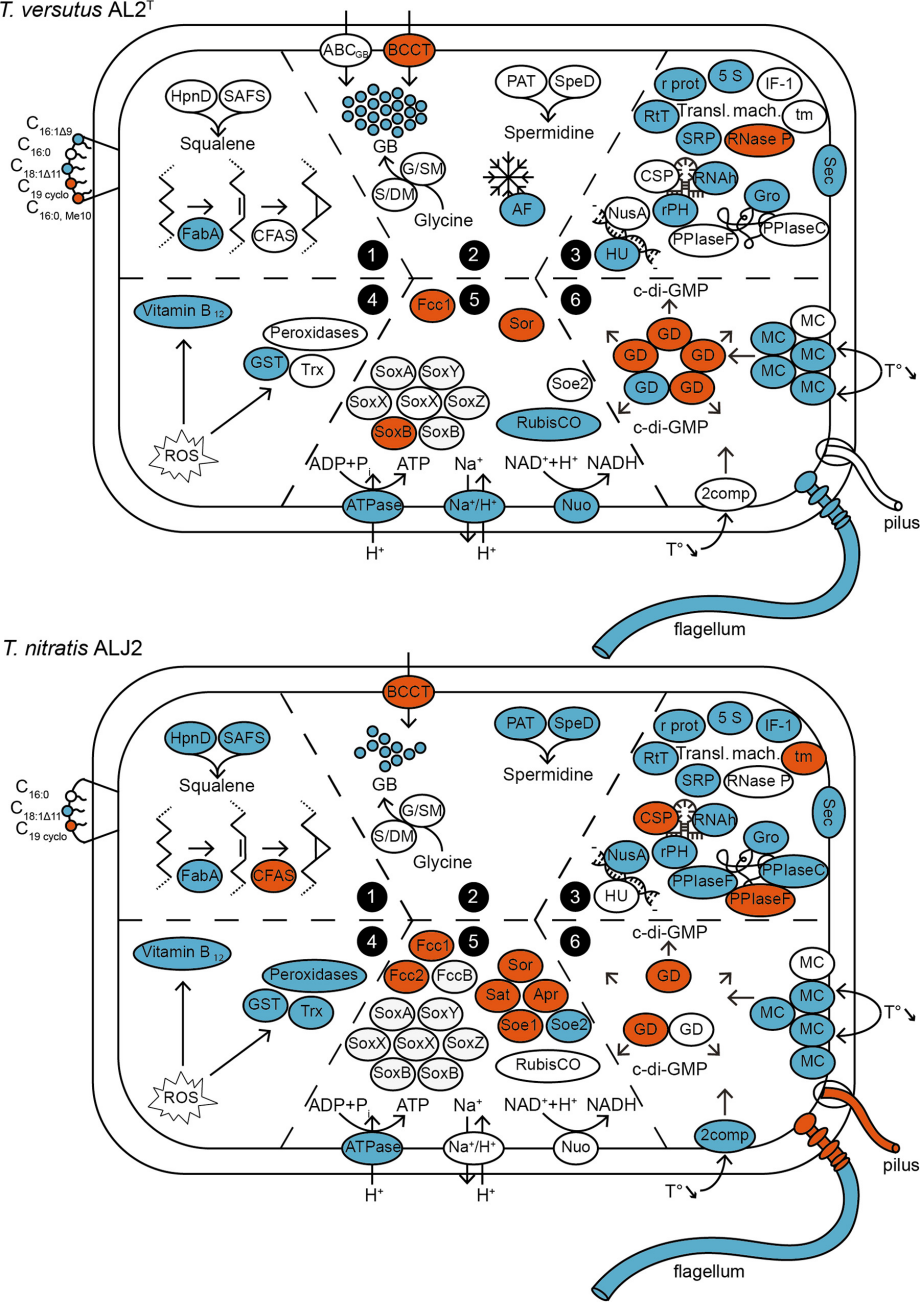
Conceptual model summarizing the responses to low temperature at the level of transcriptomics, fatty acid membrane composition, and glycine betaine concentration for *T. versutus* AL2^T^ and *T. nitratis* ALJ2. Categories 1 to 6 are described in detail in Results and Discussion. Genes that are upregulated at 10°C are depicted in blue; those that are downregulated at 10°C are in red. (Category 1) Membrane lipid composition. SAFS, squalene-associated FAD-dependent desaturase; CFAS, cyclopropane-fatty-acyl-phospholipid synthase. (Category 2) Compatible solutes and antifreeze proteins. GB, glycine betaine; ABC_GB_, ABC glycine betaine transporter; BCCT, betaine-carnitine-choline transporter; G/SM, glycine/sarcosine *N*-methyltransferase; S/DM, sarcosine/dimethylglycine *N*-methyltransferase; AF, antifreeze-like protein; PAT, polyamine aminopropyltransferase. (Category 3) Stability of nucleic acids and proteins. CSP, cold shock protein; RNAh, RNA helicase; rPH, RNase PH; HU, HU family DNA-binding protein; Gro, GroEL and GroES chaperones; PPIaseF, FKBP-type peptidyl-prolyl isomerases; PPIaseC, cyclophilin-type peptidyl-prolyl isomerases; 5S, 5S rRNA; r prot, ribosomal proteins; RtT, RtT sRNA; SRP, SRP RNA; RNase P, RNase P RNA component; tm, tmRNA; IF-1, translation initiation factor IF-1. (Category 4) Protection against oxidative stress. Trx, thioredoxin; GST, glutathione *S*-transferase family protein. (Category 5) Sulfur, carbon, and energy metabolism. RubisCO, ribulose-biphosphate carboxylase; Na^+^/H^+^, Na^+^/H^+^ antiporter. (Category 6) Chemotaxis and motility. MC, methyl-accepting chemotaxis protein; GD, GGDEF domain-containing protein; c-di-GMP, bis-(3′-5′)-cyclic dimeric GMP; 2comp, two-component system. Locus tags and differential expression values are listed in Table S9.

Temperature adaptation in both *Thioalkalivibrio* strains consisted of molecular and physiological shifts in several biological processes and components, which are depicted in a conceptual model (Fig. 1). A summary of the expression data for the different categories of genes mentioned in Fig. 1 and in the sections below is given in Table S9. Statistical differences in temperature responses between the two strains (Tables S6 and S7) are highlighted in the text by giving the Gene Ontology (GO) ID of the orthologs possessing a strain-temperature interaction.

10.1128/mSystems.01202-20.7 TABLE S6 Significant interaction effect of strain and temperature on orthogroup expression. Orthologous groups, for which the strains responded differently to temperature treatment: orthogroups that are upregulated in *T. versutus* AL2^T^ but downregulated in *T. nitratis* ALJ2 (comparatively). Orthogroup description will appear/disappear when clicking on the red diamonds. For each orthologous group, the differential expression plot will appear/disappear when clicking on the red orthogroup IDs. These pop-up box plots display bootstrap values of estimated read counts per strain and temperature regime. Functional annotations and gene assignments for each orthogroup are listed in Table S8. Download Table S6, PDF file, 0.5 MB. Copyright © 2021 Ahn et al. 2021 Ahn et al. https://creativecommons.org/licenses/by/4.0/ This content is distributed under the terms of the Creative Commons Attribution 4.0 International license.

10.1128/mSystems.01202-20.8 TABLE S7 Significant interaction effect of strain and temperature on orthogroup expression. Orthologous groups, for which the strains responded differently to temperature treatment: Orthogroups that are upregulated in *T. nitratis* ALJ2 but downregulated in *T. versutus* AL2^T^ (comparatively). Orthogroup description will appear/disappear when clicking on the red diamonds. For each orthologous group, the differential expression plot will appear/disappear when clicking on the red orthogroup IDs. These pop-up box plots display bootstrap values of estimated read counts per strain and temperature regime. Functional annotations and gene assignments for each orthogroup are listed in Table S8. Download Table S7, PDF file, 0.8 MB. Copyright © 2021 Ahn et al. 2021 Ahn et al. https://creativecommons.org/licenses/by/4.0/ This content is distributed under the terms of the Creative Commons Attribution 4.0 International license.

10.1128/mSystems.01202-20.9 TABLE S8 Functional annotations and orthology group assignments of differentially expressed genes. Blue text links to the respective web pages for the Gene Ontology (GO) ID, PFAM-A domain ID (PF), or InterProScan ID (IPR). Download Table S8, PDF file, 0.3 MB. Copyright © 2021 Ahn et al. 2021 Ahn et al. https://creativecommons.org/licenses/by/4.0/ This content is distributed under the terms of the Creative Commons Attribution 4.0 International license.

10.1128/mSystems.01202-20.10 TABLE S9 Gene expression per section, including the annotation, locus tags, *b* value, *P* value, and *P*_adj_ value. Download Table S9, PDF file, 0.3 MB. Copyright © 2021 Ahn et al. 2021 Ahn et al. https://creativecommons.org/licenses/by/4.0/ This content is distributed under the terms of the Creative Commons Attribution 4.0 International license.

### Category 1: membrane lipid composition.

Changes in temperature may have substantial effects on the fluidity of the membrane and consequently also on the functioning of membrane-associated metabolic pathways, such as respiratory electron transfer. Therefore, the adaptation of the lipid composition is crucial to counteract cold-induced membrane stiffness and to maintain the fluidity of the membrane (20, 35). The head group composition of intact polar membrane lipids did not change significantly with temperature and, for both *Thioalkalivibrio* strains, was mainly composed of phosphatidylcholine with smaller amounts of lysophosphatidylcholine and phosphatidylglycerol (Table 2). Furthermore, phosphatidylethanolamine was present at low abundance in both strains and increased in concentration at 10°C for *T. nitratis* ALJ2. Diphosphatidylglycerol was found in *T. nitratis* ALJ2, whereas it was detected only in low quantities for *T. versutus* AL2^T^ grown at 10°C (Table 2). To our knowledge, the head group composition of the intact polar membrane lipids was measured for the first time for *Thioalkalivibrio* by this study.

**TABLE 2:** Head group composition of intact polar membrane lipids and their detected total fatty acid content in *T. versutus* AL2^T^ and *T. nitratis* ALJ2 at 10°C and 30°C

Intact polar lipid	*T. versutus* AL2^T^ at:				*T. nitratis* ALJ2 at:			
	10°C		30°C		10°C		30°C	
	Quantity^*a*^	FA content^*b*^	Quantity^*a*^	FA content^*b*^	Quantity^*a*^	FA content^*b*^	Quantity^*a*^	FA content^*b*^
Phosphatidylethanolamine	*	C_34:1_, C_36:2_, C_34:2_, C_32:1_, C_37:2_	*	C_36:1_, C_35:1_	+	C_34:1_, C_36:2_	*	
Phosphatidylglycerol	+	C_34:1_, C_34:2_, C_36:2_, C_35:1_	+	C_36:1_, C_35:1_, C_38:2_	+	C_34:1_, C_36:2_	+	C_35:1_, C_37:2_, C_38:2_
Diphosphatidylglycerol	*		ND		+		+	
Phosphatidylcholine	+++	C_34:1_, C_35:1_	+++	C_36:1_, C_35:1_, C_38:2_, C_33:0_	+++	C_34:1_, C_36:2_, C_37:2_	+++	C_35:1_, C_37:2_, C_38:2_
Lysophosphatidylcholine	+	C_18:1_, C_19cyclopropyl_, C_16:0_, C_16:1_	++	C_19cyclopropyl_, 10-Me-C_16_, C_16:0_	+	C_20_H_33_O_3_	+	

a Abundance is represented by +, ++, and +++; *, result below 10% intensity; ND, not detected.

b Total fatty acids detected. For entries in the form α-Me-C_β:γ_, α indicates the position of the methyl branch from the carboxyl terminus, β is the number of carbons, and γ is the number of double bonds.

In strong contrast to the head group composition, major changes in the fatty acid (FA) composition of the cell membrane were detected as a result of the difference in cultivation temperature (Tables 3 and 4). At 10°C, a decrease of lactobacillic acid (C_19_ cyclopropyl FA) in both strains and of 10-methylhexadecanoic acid (10-Me-C_16:0_) in *T. versutus* AL2^T^, as well as an increase of octadec-11-enoic acid (C_18:1Δ11_) in both strains and of hexadec-9-enoic acid (C_16:1Δ9_) in *T. versutus* AL2^T^, was observed in comparison to the FA composition at 30°C (Fig. 1; Table 3). The saturated FA hexadecanoic acid (C_16:0_) remained abundant (20 to 24%) at both temperatures. An increase in the proportion of unsaturated FA at low temperatures is a well-known mechanism to keep the membrane fluidity optimal by lowering its phase-transition temperature (35, 36). Apart from increasing the proportion of unsaturated FAs, a reduction in chain length and increase in branching of FA are also used by bacteria as an adaptation to lower temperature (20). Therefore, it is somewhat surprising that *T. versutus* AL2^T^ reduced the amount of branched FA and both species almost maintained the ratio of shorter- to longer-chain FA (Table 4). This suggests that the high abundance of unsaturated FA provides sufficient membrane fluidity for growth at 10°C. With respect to the shift in proportion of the C_19_ cyclopropane FA, i.e., from ca. 40% at 30°C to 3 to 12% at 10°C (Table 3), its increased presence has been associated with heat resistance (37–39) but has also been detected in low-temperature adaptation (20, 38).

**TABLE 3:** Adaptations of the membrane’s fatty acid composition during growth at 10°C and at 30°C in *T. versutus* AL2^T^ and *T. nitratis* ALJ2

Fatty acid^*a*^	% of total fatty acids in^*b*^:			
	*T. versutus* AL2^T^		*T. nitratis* ALJ2	
	10°C	30°C	10°C	30°C
C_12:1Δ5_	0.2 ± 0	0.2 ± NA	1.2 ± 0.1	0.8 ± 0.1
C_12:0_	5.2 ± 0.6	6.5 ± 0.5	5.4 ± 0.3	5.7 ± 0.2
C_14:1Δ7_	0.7 ± 0.1	0.2 ± 0	ND	ND
C_14:0_	0.4 ± 0	0.3 ± 0.1	ND	ND
C_15:0_	0.4 ± 0	0.4 ± 0.1	ND	ND
C_16:1Δ5_	1.8 ± 0.4	1.9 ± 0.3	ND	ND
C_16:1Δ9_	11.5 ± 0.7	2.1 ± 0.4	0.8 ± 0.1	0.8 ± 0.1
C_16:0_	20.0 ± 0.7	21.8 ± 0.7	22.0 ± 2.6	24.5 ± 0.6
10-Me-C_16:0_	3.4 ± 0.6	19.3 ± 1.7	ND	ND
10-Me-C_16:1Δ9_	3.9 ± 0.2	1.7 ± 0.7	ND	ND
C_17:1Δ5_	0.3 ± 0	0.4 ± 0.1	ND	ND
C_17:1Δ11_	ND	ND	0.4 ± 0.1	0.3 ± NA
C_17:0_	0.5 ± 0	1.0 ± 0.1	1.3 ± 0.1	2.4 ± 0.2
C_18:1Δ5_	ND	1.0 ± 0.1	ND	ND
C_18:1Δ11_	39.5 ± 2.9	2.9 ± 0.4	63.8 ± 2.7	21.0 ± 2.8
C_18:1Δ13_	ND	ND	ND	0.7 ± 0.1
C_18:0_	0.4 ± 0.1	1.6 ± 0.1	2.2 ± 0.2	3.6 ± 0.3
12-Me-C_18:0_	0.2 ± NA	0.8 ± 0.1	ND	ND
C_19 cyclopropyl_	11.9 ± 2.4	38.2 ± 1.0	3.0 ± 0.8	40.5 ± 2.3

a For entries in the form α-Me-C_β:γΔδ_, α is the position of the methyl branch from the carboxyl terminus, β is the number of carbons, γ is the number of double bonds, and Δδ is the position of the double bond, counting from the carboxyl terminus.

b ND, not detected; NA, not applicable.

**TABLE 4:** Features of the fatty acid composition during growth at 10°C and at 30°C in *T. versutus* AL2^T^ and *T. nitratis* ALJ2

Organism and growth temp (°C)	Short-chain/long-chain FA^*a*^	% unsaturated FA	% branched FA
*T. versutus* AL2^T^			
10	0.9	57.9	7.5
30	1.2	10.4	21.9
*T. nitratis* ALJ2			
10	0.4	66.3	0
30	0.5	23.5	0

a The ratio of the FA with a chain length < C_17_ to the FA with a chain length of ≥ C_17_.

The changes in FA composition correlated with the differential expression of genes responsible for the membrane polar lipid biosynthesis of both *Thioalkalivibrio* strains (Fig. 1; Tables 3 and 4; Table S9). Desaturation of FAs is performed either by the fatty acid desaturases (Des) or by the FabA dehydratase/isomerase, which replaces single bonds with doubled bonds in the carbon chain (40, 41). The genes *desA* and *desC*, coding for Δ12 and Δ9 desaturases, respectively, were detected only in the genome of *T. versutus* AL2^T^ but were not differentially expressed at 10°C. However, at 10°C, both strains upregulated *fabA* (Table S5), and *T. nitratis* ALJ2 also upregulated a malonyl coenzyme A (malonyl-CoA)-acyl carrier protein transacylase gene, *fabD*. Thus, the Fab pathway appears to be a major desaturation mechanism at low temperatures in *Thioalkalivibrio*. This is in good agreement with the fact that the abundant unsaturated FAs (C_16:1Δ9_ and C_18:1Δ11_) are omega-7 FAs, which are produced by the Fab pathway. Lactobacillic acid is produced from C_18:1Δ11_ FA by cyclopropanation by the cyclopropane-fatty-acyl-phospholipid synthase. The methylene donor is a methyl group on *S*-adenosylmethionine (42). Multiple genes encoding this enzyme were detected in the genomes of both strains. Two of them were significantly downregulated at 10°C in *T. nitratis* ALJ2 but not in *T. versutus* AL2^T^ (strain-temp interaction for OG0001444) (Table S7).

10.1128/mSystems.01202-20.6 TABLE S5 Differential orthogroup expression in response to temperature: Orthologous groups that were consistently upregulated at 10°C compared to 30°C. For each orthologous group, the differential expression plot will appear/disappear when clicking on the red orthogroup IDs. These pop-up box plots display bootstrap values of estimated read counts per strain and temperature regime. Functional annotations and gene assignments for each orthogroup are listed in Table S8. Download Table S5, PDF file, 0.2 MB. Copyright © 2021 Ahn et al. 2021 Ahn et al. https://creativecommons.org/licenses/by/4.0/ This content is distributed under the terms of the Creative Commons Attribution 4.0 International license.

Other differentially expressed genes involved in the membrane lipid biosynthesis included in *T. nitratis* ALJ2 the upregulation at 10°C of the squalene-associated FAD-dependent desaturase *hpnE* and the squalene synthase *hpnD*, which are both involved in the production of the neutral lipid squalene (Fig. 1; Table S9). Interestingly, squalene was only detected in a minor fraction (<1%) of the total lipids, even though it was found in high concentrations in the *Thioalkalivibrio* strain ALJ15 (43), and in *T. paradoxus* ARh1^T^ its derivative lanosterol constituted up to 50% of the total lipids (44). Furthermore, multiple genes coding for proteins involved in peptidoglycan, lipoprotein, and lipopolysaccharide synthesis were found to be differentially expressed in both strains as well as a decrease in expression at 10°C for a range of genes containing a PEP-CTERM domain (Table S9). PEP-CTERM domain-containing proteins are believed to constitute a protein export sorting system, which is linked to exopolysaccharide protein expression (45).

### Category 2: compatible solutes and antifreeze proteins.

**FIG 2:**
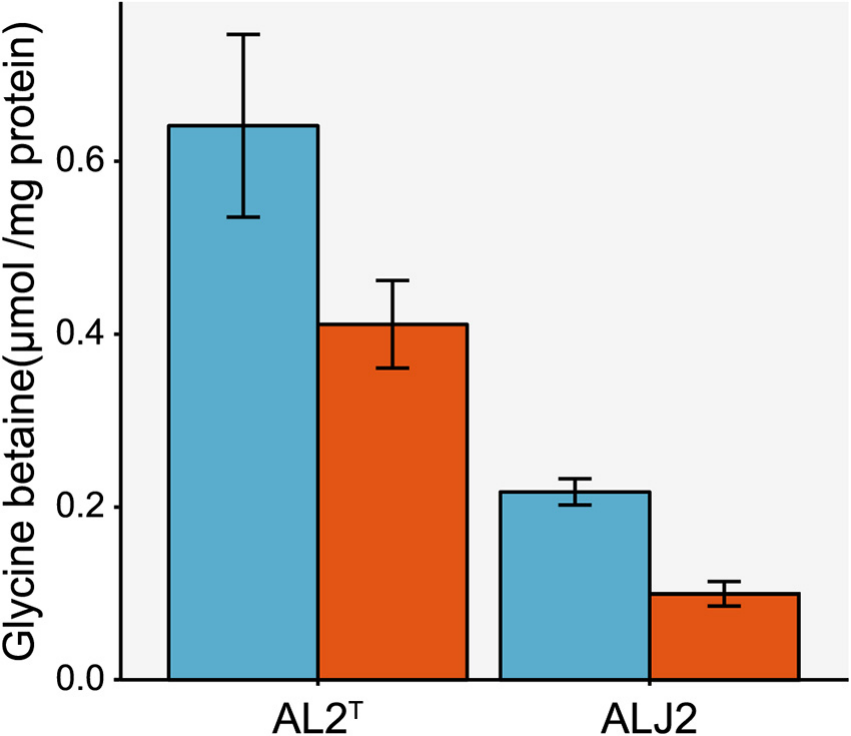
Intracellular glycine betaine content of *T. versutus* AL2^T^ and *T. nitratis* ALJ2 at 10°C and 30°C. The glycine betaine concentration at 10°C is shown in blue, and that at 30°C is in red. The error bars depict the standard deviations of the averages.

A key adaptation to reduced temperature is the production of compatible solutes and antifreeze proteins. Among these compatible solutes, glycine betaine is, apart from its feature as an osmolyte (46), also known to enhance cryotolerance in bacteria (21, 47–49). Accordingly, the glycine betaine concentration in both strains was higher at 10°C than at 30°C (Fig. 2). Moreover, the glycine betaine content was 3-fold higher for *T. versutus* AL2^T^ than for *T. nitratis* ALJ2 at 10°C (Fig. 1 and 2). Hence, *T. versutus* AL2^T^ appears to have a clear advantage for growing at low temperatures compared to *T. nitratis* ALJ2. Osmolytes provide cryotolerance by protecting the cytoplasmic proteins from denaturation during freezing (50) and by reducing the cytoplasmic freezing point, which prevents the formation of ice crystals inside the cell (51, 52).

Glycine betaine can be taken up by the cell via the betaine-carnitine-choline transporter (BCCT) (53) or by the ATP-driven ABC glycine betaine transporter (54). Both *Thioalkalivibrio* strains contain four genes for BCCT, and *T. versutus* AL2^T^ also possesses the ATP-dependent betaine transporter. Interestingly, one of the annotated BCCT genes is downregulated in both strains at 10°C, whereas the others are not differentially expressed (Fig. 1; Table S9).

Another option for intracellular accumulation of glycine betaine is *de novo* synthesis from the precursor choline (55) or from glycine in a three-step methylation process (56). Neither *Thioalkalivibrio* strain encodes BetABI, which is responsible for the synthesis of glycine betaine from choline (55). However, both strains do possess the *de novo* synthesis pathway from glycine, which is catalyzed by two methyltransferases, glycine sarcosine methyltransferase and sarcosine dimethylglycine methyltransferase (56). Both methyltransferases are slightly upregulated in *T. nitratis* ALJ2 (*b* values of 0.69 [*P*_adj_ = 1.31 × 10^−7^] and 0.89 [*P*_adj_ = 2.30 × 10^−12^], respectively).

Some bacteria also shield themselves from intracellular ice formation by the production of antifreeze or ice-binding proteins, which bind to ice crystals and prevent them from growing (57). A gene encoding such an antifreeze-like protein was overexpressed in *T. versutus* AL2^T^ at the low temperature (Fig. 1; Table S9) but was not detected in the genome of *T. nitratis* ALJ2. Another molecule protecting cells against cold stress is the polyamine spermidine (58). This might be explained by its properties of protecting DNA from oxidative damage, intervening in transcriptional and translational regulation, and playing a role in the structure and the stability of nucleic acids (59). At 10°C, *T. nitratis* ALJ2 upregulates the expression of genes for two enzymes facilitating spermidine production, polyamine aminopropyltransferase and adenosylmethionine decarboxylase (Fig. 1; Table S9), while their expression is consistently high in *T. versutus* AL2^T^ (strain-temp interactions for OG0001863 and OG0000401) (Table S6).

### Category 3: stability of nucleic acids and proteins.

A decrease in temperature causes an increase in the negative supercoiling of DNA (23) and also an increase in the stability of the secondary structures of mRNA (24, 25), which negatively affects the efficiency of replication, recombination, transcription, and translation (28). Furthermore, a decrease in temperature can lead to protein misfolding (29). To counteract these effects, genes coding for helicases, chaperones, and proteins of the transcription and translational machinery were generally upregulated in both *Thioalkalivibrio* strains growing at 10°C (Fig. 1; Table S9).

Multiple genes annotated for nucleotide synthesis and repair were upregulated with growth at the low temperature in both strains (Table S9). Furthermore, several transcription regulators were differentially expressed in *Thioalkalivibrio*. Cold shock proteins (CSP) counteract the formation of secondary structures in mRNA and thereby improve the transcription and translation efficiency by acting as chaperones of nucleic acids (28). Interestingly, only one CSP was slightly upregulated in *T. nitratis* ALJ2, whereas the other was downregulated (*b* values of 0.68 [*P*_adj_ = 6.63 × 10^−5^] and −1.30 [*P*_adj_ = 3.53 × 10^−2^], respectively). Other low-temperature-induced genes involved in transcription included the genes for DEAD box-containing RNA helicase (*rhlE*) and the RNase PH in both strains (Table S5), the DNA-binding protein of the HU family in *T. versutus* AL2^T^, and the transcription termination/antitermination factor NusA in *T. nitratis* ALJ2 (Fig. 1; Table S9).

Both strains induced the gene expression of multiple tRNAs, genes involved in the synthesis of tRNAs and amino acids, the 5S rRNA and several ribosomal protein-encoding genes (Fig. 1; Table S9). Multiple noncoding RNAs (ncRNA) implicated in translation were upregulated as well and include RtT sRNA (small RNA processed from the *tyrT* transcript) and signal recognition particle (SRP) RNA, whereas the RNase P RNA component and the tmRNA were downregulated (Fig. 1; Table S9). Furthermore, the gene encoding the translation initiation factor IF-1 was upregulated at 10°C in *T. nitratis* ALJ2, while it was consistently highly expressed in *T. versutus* AL2^T^ (strain-temp interaction for OG0000216) (Fig. 1; Table S6).

The genes of the chaperones GroEL and GroES of the Hsp60 system were highly expressed in both *Thioalkalivibrio* strains at 10°C (Fig. 1; Table S5). These chaperones enable the proper folding of proteins, which is essential under denaturing conditions (30), such as cold stress (29). In contrast, Hsp20 heat shock proteins were downregulated at 10°C in the two tested *Thioalkalivibrio* strains (Table S4). Other protein chaperones induced under cold stress are the peptidyl-prolyl isomerases (PPIase), including the FKBP (FK506 binding protein) and the cyclophilin types (60–62). In *T. nitratis* ALJ2, a FKBP- and a cyclophilin-type PPIase were strongly upregulated at 10°C, but another FKBP-type PPIase was highly downregulated (Fig. 1; Table S9). Contrary to the effect in *T. nitratis* ALJ2, the cyclophilin-type PPIase was consistently highly expressed in *T. versutus* AL2^T^ (strain-temp interaction for OG0001177) (Table S6).

10.1128/mSystems.01202-20.5 TABLE S4 Differential orthogroup expression in response to temperature: Orthologous groups that were consistently upregulated at 30°C compared to 10°C. For each orthologous group, the differential expression plot will appear/disappear when clicking on the red orthogroup IDs. These pop-up box plots display bootstrap values of estimated read counts per strain and temperature regime. Functional annotations and gene assignments for each orthogroup are listed in Table S8. Download Table S4, PDF file, 0.3 MB. Copyright © 2021 Ahn et al. 2021 Ahn et al. https://creativecommons.org/licenses/by/4.0/ This content is distributed under the terms of the Creative Commons Attribution 4.0 International license.

Finally, the *secD* and *yajC* gene components of the general secretory pathway (Sec) were upregulated in both *Thioalkalivibrio* at 10°C (Fig. 1; Table S5). The complex formed by SecDF-YajC might play an important role in cold adaptation, as inactivation of this complex induces cold sensitivity (63).

### Category 4: protection against oxidative stress.

With the reduction of temperatures, oxygen solubility in the medium increases (32), which can lead to the formation of reactive oxygen species (ROS), oxidative stress, and subsequently damage to DNA, proteins, and lipids (31). Neither a superoxide dismutase nor a catalase was differentially expressed at 10°C in the two *Thioalkalivibrio* strains. However, multiple peroxiredoxins were upregulated in *T. nitratis* ALJ2 (strain-temp interaction for OG0001493) (Table S6), and a glutathione *S*-transferase was upregulated in both strains at 10°C (Fig. 1; Table S9).

In contrast, genes involved in vitamin B_12_ biosynthesis were strongly upregulated at 10°C for both strains (Fig. 1; Tables S5 and S9). Interestingly, genes for vitamin B_12_ production were also highly upregulated in *Thioalkalivibrio* under arsenite stress, where vitamin B_12_ was previously proposed as an antioxidant in this bacterium (64). Its antioxidant capacity has already been demonstrated in both prokaryotic and eukaryotic cells under oxidative stress (65–67). Furthermore, for cold stress, genes encoding vitamin B_12_ biosynthesis have been shown to be expressed and linked to oxidative stress protection (60, 68).

### Category 5: sulfur, carbon, and energy metabolism.

*Thioalkalivibrio* strains are sulfur-oxidizing bacteria (17) that under standard thiosulfate-limited steady state conditions completely oxidize thiosulfate to sulfate. However, accumulation of elemental sulfur was observed in the chemostats of both *Thioalkalivibrio* strains growing at 10°C, whereas elemental sulfur was not observed in the chemostats at 30°C. This might indicate a decrease in the metabolic activity of the second oxidation step from elemental sulfur to sulfate compared to the first oxidation step of thiosulfate to elemental sulfur at low temperatures. Indeed, the multienzyme *sox* system, which is responsible for the oxidation of thiosulfate and the release of sulfate and SoxY-bound sulfane sulfur (69, 70), did not change significantly in expression, except for the downregulation of *soxB* in *T. versutus* AL2^T^. Interestingly, the heterodisulfide reductase (*hdr*) genes, which are thought to be responsible for the further processing of the sulfane sulfur (71), also did not change their expression.

In contrast, genes involved in the transformation of sulfite to sulfate were downregulated at the low temperature in both *Thioalkalivibrio* strains. These include genes for the indirect sulfite oxidation pathway with *sat* and *aprAB* (72, 73), which are present only in *T. nitratis* ALJ2, and genes for the two direct pathways, including the quinone-dependent sulfite oxidoreductase SoeABC of cluster 1 (as defined in reference 64) (74) and the cytochrome *c*-dependent oxidoreductase SorAB (Table S4) (72). While both strains downregulated *sorA* at 10°C, the effect was more pronounced in *T. nitratis* ALJ2 (strain-temp interaction for OG0000759) (Table S7). The genes encoding SoeABC of cluster 2 (as defined in reference 64), however, were upregulated in *T. nitratis* ALJ2. Furthermore, the sulfide dehydrogenase gene *fccAB* was also downregulated at 10°C in both *Thioalkalivibrio* strains (Fig. 1; Table S9). A decrease in the expression of genes responsible for the oxidation of elemental sulfur to sulfate at the low temperature was also observed for the psychrotolerant bacterium Acidithiobacillus ferrivorans SS3 at 8°C, where *hdr*, *sat*, and *apr* were downregulated and *sox* genes were significantly upregulated (68).

*T. versutus* AL2^T^ appears to increase its capacity to fix carbon in response to growth at low temperatures, as it induces the transcription of the small and the large subunit of RubisCO (ribulose-biphosphate carboxylase), as well as a subunit of the carboxysome. Moreover, *T. nitratis* ALJ2 upregulates a carbon storage regulator *csrA* (Fig. 1; Table S9). These results suggest an increased carbon demand at lower temperatures.

Looking at genes encoding electron transport chain proteins, it can be seen that the NuoL subunit of NADH-quinone oxidoreductase was significantly induced in *T. versutus* AL2^T^ and the NuoM subunit was downregulated at 30°C in *T. versutus* AL2^T^ but not in *T. nitratis* ALJ2 (strain-temp interaction for OG0000591) (Fig. 1; Table S7). The induction of NADH-quinone oxidoreductase subunits at low temperatures was also found in other studies (68, 75). Furthermore, at the low temperature, multiple subunits of the ATP synthase were also significantly induced in both *Thioalkalivibrio* strains and a gene encoding a Na^+^/H^+^ antiporter was upregulated in *T. versutus* AL2^T^ (Fig. 1; Tables S5 and S9). The upregulation of genes involved in the energy generation reflects an increased need for ATP to perform adaptations in response to low temperature, for example, the *de novo* production of glycine betaine (76).

### Category 6: chemotaxis and motility.

Organisms can sense fluctuations in environmental temperatures by changes in the membrane fluidity, as well as by structural changes in DNA, RNA, and proteins (25, 77). These signals are transmitted and can induce physiological adaptations to cope with a change in environmental temperature. For example, two-component systems with sensor histidine kinases and response regulator proteins are known to be involved in the sensing and transmitting of environmental signals, including temperature (78). Methyl-accepting chemotaxis proteins were found to be involved in sensing temperature in Escherichia coli and to alternate accordingly its swimming behavior (25). Indeed, gene expression results for *Thioalkalivibrio* grown at 10°C revealed several genes encoding methyl-accepting chemotaxis proteins and a gene encoding a two-component system protein with differential expression (Fig. 1; Table S9). Moreover, multiple genes encoding GGDEF-domain proteins, which are involved in signal transduction by producing the secondary messenger bis-(3′-5′)-cyclic dimeric GMP (c-di-GMP) (79), were found to be differentially expressed as well (Fig. 1; Tables S4, S5, and S9). A GGDEF domain-containing protein (OG0000928) is upregulated at 10°C in *T. versutus* AL2^T^ but downregulated in *T. nitratis* ALJ2, while a methyl-accepting chemotaxis protein (OG0000306) is strongly downregulated at 10°C in *T. nitratis* ALJ2. Furthermore, a methyl-accepting chemotaxis protein (OG0001236) is strongly upregulated at 10°C for both strains (strain-temp interactions for OG0000928, OG0000306, and OG0001236) (Table S7).

Flagella are important for the motility of bacteria in their aqueous environment and to respond to chemotaxis (80), as are type IV pili enabling twitching motility on surfaces (81). Furthermore, gene expression of flagellum components and motility is primarily influenced by various environmental conditions in bacteria (82, 83). In particular, movement rates of the flagellum and, with this, swimming speed are physically directly influenced by temperature (84). *Thioalkalivibrio* strains are motile with a single polar flagellum (17). At the low temperature, all *fli*, *flg*, and *flh* flagellum-building blocks as well as genes assisting in the assembly of the flagellum (*flgANM* and *fliST*) were upregulated for *T. versutus* AL2^T^. In contrast, for *T. nitratis* ALJ2, genes for structural flagellum proteins were downregulated, with the exception of the genes encoding the filament and the filament cap of the flagellum (*fliCD*) and their respective chaperones (*fliST*) (85) (Fig. 1; Tables S4, S5, and S9). However, comparing expression levels of the flagellum genes of both strains to each other revealed that both strains shift to a similar level at 10°C (strain-temp interactions for OG0000450-52, OG0000486-9, OG0001024-28, OG0001070-71, OG0001073-74, OG0001257-58, OG0001286-87, OG0001559-64, OG0001613-15, OG0001644-50, OG0002008, OG0002052, and OG0002056) (see the box plots in Table S7). Type IV pilus synthesis genes did not change their expression with temperature in *T. versutus* AL2^T^, but certain of these genes showed a downregulation at 10°C in *T. nitratis* ALJ2 (strain-temp interactions for OG0001191, OG0001834, OG0001850, OG0002020, and OG0002096) (Fig. 1; Tables S7 and S9). When the flagellum is damaged, the cell is able to repair it by incorporating new flagellum protein units (86, 87). The upregulation of the flagellum units in *T. versutus* AL2^T^ could be explained by the increased need for repair at low temperatures. However, *T. nitratis* ALJ2 might reduce its mobility under these shearing circumstances and thus downregulate its flagellum genes.

### Conclusion.

In this study, we investigated the low-temperature responses of two *Thioalkalivibrio* strains isolated from soda lakes with different temperature regimes. *T. versutus* AL2^T^, which originates from a soda lake with freezing periods during winter, had a clear growth advantage at 10°C compared to *T. nitratis* ALJ2. Remarkably, *T. nitratis* ALJ2 exhibited an adaptation response to the low temperature similar to that of *T. versutus* AL2^T^, even though *T. nitratis* ALJ2 was isolated from a Kenyan soda lake with a stable warm temperature the year round. The exception was the 3-fold-higher concentration of the osmolyte glycine betaine in *T. versutus* AL2^T^, identifying this compatible solute as an essential actor in cryoprotection of the cold-adapted strain. We hypothesize that the adaptation to low-temperature stress invokes multiple “basic” stress responses that trigger inherent protection mechanisms against, e.g., oxidative stress or protein denaturation. Moreover, glycine betaine is also one of the key factors in protection against osmotic stress. Therefore, even though *T. nitratis* ALJ2 does not face cold stress in its environment, it must cope with other common soda lake stressors, such as high UV radiation or high salinity. This study enlightens differences and commonalities in adaptation by strains of the same genus with different temperature regime backgrounds and contributes to the general understanding of low-temperature adaptation in bacteria.

## MATERIALS AND METHODS

### Strains and growth conditions.

Growth parameters (lag phase [hours], maximum growth rate [μ_max_] [per hour], and stationary phase [cells per milliliter]) of *T. versutus* AL2^T^ and *T. nitratis* ALJ2 were determined from axenic cultures grown in duplicate 500-ml batch-mode reactors at 10°C and 30°C by cell count measurements using flow cytometry. The batch reactors were magnetically stirred at 700 rpm and aerated with 0.5 liter/min of compressed air. The medium contained 17.5 g/liter Na_2_CO_3_, 13.9 g/liter NaHCO_3_, 6.1 g/liter NaCl, 1 g/liter K_2_HPO_4_, 0.2 g/liter MgCl_2_, 40 mM Na_2_S_2_O_3_, 5 mM KNO_3_ and 1:1,000 trace metals (88). As the pH is influenced by temperature, it was adjusted to 9.8 at 10°C and 30°C. To obtain the growth parameters, the cell count measurements of the batch cultures were fit into a logistic fit model using the R package GroFit (89) (lag phase) and Jupyter Notebook (https://jupyter.org) (maximal growth rate and stationary phase).

To study the response to low temperature, *T. versutus* AL2^T^ and *T. nitratis* ALJ2 were grown at 10°C and 30°C in 500-ml chemostat reactors under continuous cultivation with a dilution rate of 0.010 ± 0.00027/h. The dilution rate was regularly monitored by measuring the pump rate over time with a burette placed between the medium vessel and the peristaltic pump. The chemostats were continuously aerated with compressed air at 0.5 liter/min and magnetically stirred at 600 rpm. Metallic baffles inside the reactor were used to achieve full aeration. All reactors were inoculated with 20 ml of bacterial cultures and were kept in batch mode for 2 days at 30°C to obtain a dense culture. The 30°C reactors were then switched to a continuous culture mode, whereas the 10°C reactors were transferred to 10°C and kept there for 4 days to acclimatize before being switched to a continuous culture mode. The composition of the growth medium for the continuous cultivation was kept the same as described for the batch cultivation except for using 20 mM Na_2_S_2_O_3_ and an increase of the pH to 10. Per condition, two reactors were run in parallel and each reactor was run twice using approximately 100 ml from the previous steady state culture as starting material, providing in the end four replicates for each condition. Figure S1A gives a schematic illustration of the experimental chemostat setup. Cell material was harvested at steady state, i.e., after at least five volume changes of the reactor. To assess steady state, cell count measurements by flow cytometry were performed (Fig. S1B) during the entire chemostat run and thiosulfate and sulfate concentrations were measured during the 3 days before the culture entered steady state growth (Table S1).

10.1128/mSystems.01202-20.2 TABLE S1 Thiosulfate and sulfate concentrations measured during the last three days before steady state in the chemostat cultivation. nd, not detected. Download Table S1, PDF file, 0.02 MB. Copyright © 2021 Ahn et al. 2021 Ahn et al. https://creativecommons.org/licenses/by/4.0/ This content is distributed under the terms of the Creative Commons Attribution 4.0 International license.

### Cell counting by flow cytometry.

Cell counting by flow cytometry was first used to determine the growth curves of the batch cultivation and subsequently the growth parameters, which were used to set up the continuous cultivation experiment. Second, flow cytometry was also used to count cells during the continuous cultivation experiment in order to assess the presence of the steady state at the sampling time.

Cell material was harvested from the reactors throughout the experiment and fixed with formaldehyde at a final concentration of 1% (wt/vol). The samples were incubated for 1 h at 4°C and thereafter centrifuged at 19,000 × *g* for 10 min at 4°C. The supernatant was discarded, and the cell pellet was dissolved in 0.5 ml TE buffer (10 mM Tris-HCl, 1 mM disodium EDTA [pH 8.0]), directly flash-frozen in liquid nitrogen, and stored at −80°C until analysis.

For the flow cytometry analysis, the samples were diluted in TE buffer to reach cell counts below 2,500 events per μl. Samples were preheated for 5 min to 35°C, and SYBR green I (Invitrogen, Carlsbad, CA, USA) was added. The stained samples were incubated for 10 min at 35°C and then kept in the dark until measurement. Flow cytometry was performed on a BD Accuri C6 flow cytometer (BD Bioscience, Franklin Lakes, NJ, USA) with 50 μl of cell sample with a medium flow rate (35 μl/min). To distinguish the events of stained bacterial cells from the background noise, a dot plot of the FL1-A (excitation at 488 nm, fluorescence at 533/30 nm) and FL3-A (excitation at 488 nm, fluorescence at 670 nm) was used that was gated from the counts of FL1-A between fluorescence values of 10,000 and 1,000,000. A threshold at 10,000 on FL-1 H was set.

### Analysis of thiosulfate and sulfate.

Thiosulfate and sulfate were analyzed using a protocol as published before (90, 91). Briefly, samples were filtered over 0.2-μm polyethersulfone (PES) membrane filters (VWR International, Radnor, PA, USA) and stored at −20°C until further processing. Thiosulfate and sulfate concentrations were measured by ion chromatography (Compact IC 761) (Metrohm, Herisau, Switzerland) with an anion column (Metrohm Metrosep A Supp 5; 150/4.0 mm) equipped with a precolumn (Metrohm Metrosep A Supp 4/5 Guard). The ion chromatography system included a chemical suppressor (Metrohm), a CO_2_ suppressor (853; Metrohm), and a conductivity detector (Metrohm). In addition, suppressors for eluent conductivity and carbon dioxide were used (Metrohm). The mobile phase consisted of 3.2 mM sodium carbonate, 1 mM sodium bicarbonate solution, and 1% (vol/vol) acetone and had a flow rate of 0.7 ml/min.

### RNA sequencing and data analysis.

Bacterial cells were harvested from the steady state cultures, and the RNA was extracted and sequenced (RNA sequencing [RNA-Seq]) as described previously (64). The reference sequences of *T. versutus* AL2^T^ (NZ_MVAR00000000.1) and *T. nitratis* ALJ2 (NZ_ARKB00000000.1) were downloaded from the NCBI RefSeq FTP server. For the general transcriptomic analysis, RNA-Seq reads were analyzed using the software programs kallisto v0.44.0 (92) and sleuth v0.30.0 (93) as described previously (64) (Table S2). A gene was considered differentially expressed if |*b*| was >1 and its *P*_adj_ value was <0.05. The *b* value is defined as a biased estimator of the log(fold change) on a natural-log scale (93). General information on the RNA-Seq reads is presented in Table S3.

10.1128/mSystems.01202-20.3 TABLE S2 Differential expression data for the RNA-Seq samples of *T. versutus* AL2^T^ (10°C/30°C) and *T. nitratis* ALJ2 (10°C/30°C). The tables include the locus tag, *P* value, *q* value (*P*_ajd_ value), *b* value (beta value), ste of *b* value (standard error of the beta value), mean of obs (mean of natural log counts of observations), var of obs (variance of observation), tech var (technical variance of observation from the bootstraps), sigma sq (raw estimator of the variance once the technical variance has been removed), smooth sigma sq (smooth regression fit for the shrinkage estimation), final sigma sq (max[sigma sq, smooth sigma sq]), protein product and the raw read counts for the different RNA-Seq samples. Download Table S2, XLSX file, 1.2 MB. Copyright © 2021 Ahn et al. 2021 Ahn et al. https://creativecommons.org/licenses/by/4.0/ This content is distributed under the terms of the Creative Commons Attribution 4.0 International license.

10.1128/mSystems.01202-20.4 TABLE S3 General information on the individual RNA-Seq samples. Download Table S3, PDF file, 0.02 MB. Copyright © 2021 Ahn et al. 2021 Ahn et al. https://creativecommons.org/licenses/by/4.0/ This content is distributed under the terms of the Creative Commons Attribution 4.0 International license.

To assess strain-specific gene expression responses to temperature, one-to-one orthologs were identified with OrthoFinder v2.3.11 (94) and analyzed with kallisto v0.46.2 and sleuth v0.30.0. Main effects of strain and temperature were evaluated with the Wald test, and their interaction effect with a likelihood ratio test (LRT). Functional annotations were obtained with InterProScan v5.40-77.0 (95) including Gene Ontology (GO) terms, PFAM A domain content (database version 32), and InterProScan terms (Tables S4 to S8).

### Membrane lipid analysis.

Bacterial cells were harvested as for RNA-Seq analysis, stored at −80°C, and then lyophilized. The lyophilized cells were hydrolyzed with 1 N KOH-methanol (96%) by refluxing for 1 h. The hydrolysate was adjusted to pH 4 with 2 N HCl-methanol, and after addition of H_2_O, it was extracted with dichloromethane (DCM). The fatty acids in this DCM extract were converted to fatty acid methyl esters (FAMEs) by methylation with diazomethane. FAMEs were analyzed by gas chromatography (GC) and GC-mass spectrometry (GC-MS) as described previously (96). Double-bond positions of the monounsaturated FAMEs were determined using the mass spectra of their dimethyl disulfide derivatives as described previously (97). The measurement was done on three samples per condition.

The intact polar lipids (IPLs) were extracted from the lyophilized cells using a modified Bligh-Dyer technique and analyzed by high-performance liquid chromatography (HPLC)–electrospray ionization (ESI)–MS as described previously (96). The measurement was done on two samples per condition.

### Determination of the concentration of glycine betaine.

*T. versutus* AL2^T^ and *T. nitratis* ALJ2 were grown in triplicate at 10°C and 30°C in 1-liter batch cultures in the culture medium that was used for the chemostat experiment. These cultures had to be produced separately, as the continuous cultivation experiments did not generate enough biomass to perform all the analyses. Cultures were centrifuged at 10,000 × *g* for 5 min and the supernatant was removed. The pellet was resuspended in a small volume, transferred to a 50-ml Greiner tube, and centrifuged at 7,000 × *g*. The pellets were stored at −20°C and then freeze-dried. From this, 30 mg of dry bacterial biomass was used for extraction with 500 μl methanol-chloroform-water (10:5:4) according to a modified protocol from reference 98 as described in reference 99. Phase separation was achieved by adding 130 μl chloroform and 130 μl water, followed by a 5-min centrifugation step at 10,000 × *g*. The upper, polar phase was removed and evaporated at reduced pressure in a SpeedVac vacuum concentrator at 50°C and 1,500 Pa. The residue was dissolved in 100 μl of an 80% (vol/vol) acetonitrile-water mixture and subsequently analyzed by isocratic HPLC on a LiChroCART aminopropyl column (Merck, Darmstadt, Germany) with 80% (vol/vol) acetonitrile-water as the mobile phase and at a flow rate of 1 ml/min.

The remaining lower chloroform phase (including the layer of insoluble cell material) was evaporated overnight. The dry residue was resuspended in 1 ml of 0.1 M NaOH and boiled for 5 min. The solubilized material was diluted and subjected to total protein determination using the Pierce bicinchoninic acid (BCA) protein assay kit (Thermo Fisher Scientific, Waltham, MA, USA) according to the recommendations of the manufacturer. The total protein content was measured to standardize the glycine betaine concentration.

### Data availability.

The raw RNA-Seq data sets have been deposited in the NCBI Sequence Read Archive under SRA accession numbers SRX7551034 to SRX7551049.

## Supplementary Material

Reviewer comments
