# Supplementary material for: Molecular and Physiological Adaptations to Low Temperature in Thioalkalivibrio Strains Isolated from Soda Lakes with Different Temperature Regimes
Source: mSystems. 2021 Apr 27;6(2):e01202-20. doi: 10.1128/mSystems.01202-20 (PMC8092127; doi:10.1128/mSystems.01202-20)
Supplement: TABLE S6 [file msystems.01202-20-st006.pdf]

Orthogroup	LRT Mean	LRT stat	LRT $b \pm se$	LRT P_{adj}	$AL2^T$: 30°C vs 10°C b	$AL2^T$: 30°C vs 10°C P_{adj}	$ALJ2$: 30°C vs 10°C b	$ALJ2$: 30°C vs 10°C P_{adj}	10°C: $ALJ2$ vs $AL2^T$ b	10°C: $ALJ2$ vs $AL2^T$ P_{adj}	30°C: $ALJ2$ vs $AL2^T$ b	30°C: $ALJ2$ vs $AL2^T$ P_{adj}
✦OG0000343	3.51	24.56	3.53±0.37	8.003e-06	3.81	8.274e-23	0.28	5.088e-01	0.39	3.491e-01	-3.13	1.994e-16
✦OG0000626	3.88	39.29	2.95±0.18	1.733e-08	2.50	8.184e-40	-0.45	2.032e-02	0.85	7.649e-06	-2.10	1.557e-29
✦OG0001492	6.68	45.72	2.87±0.14	1.290e-09	0.97	9.242e-11	-1.90	2.021e-38	0.78	1.755e-07	-2.09	4.022e-47
✦OG0001493	7.89	52.66	2.58±0.10	1.252e-10	0.82	1.366e-14	-1.76	1.893e-63	0.01	9.406e-01	-2.58	4.161e-136
✦OG0000925	4.27	25.25	2.43±0.25	5.955e-06	2.88	1.093e-28	0.45	9.685e-02	0.64	1.595e-02	-1.79	3.264e-12
✦OG0000691	5.03	41.58	2.43±0.14	6.924e-09	-0.35	1.962e-02	-2.79	4.895e-86	-0.17	2.709e-01	-2.60	2.891e-76
✦OG0000439	4.35	36.81	2.05±0.14	5.071e-08	2.61	6.587e-74	0.56	1.664e-04	0.69	2.036e-06	-1.36	2.049e-21
✦OG0001489	5.58	42.40	1.79±0.10	5.210e-09	1.03	2.181e-23	-0.76	2.098e-13	1.67	3.522e-61	-0.12	2.742e-01
✦OG0001867	5.80	50.01	1.70±0.09	2.892e-10	0.64	1.295e-11	-1.06	1.604e-29	1.16	6.397e-36	-0.54	1.143e-08
✦OG0002040	5.55	42.02	1.62±0.09	6.110e-09	1.03	1.199e-25	-0.60	1.377e-09	0.20	5.150e-02	-1.42	6.184e-50
✦OG0000309	5.83	41.11	1.53±0.09	8.014e-09	0.39	4.487e-05	-1.14	2.616e-34	0.93	1.008e-23	-0.60	1.842e-10
✦OG0001526	4.66	27.96	1.49±0.14	1.919e-06	0.46	2.055e-03	-1.03	4.513e-13	1.23	4.210e-18	-0.26	7.937e-02
✦OG0000690	4.39	20.30	1.44±0.18	5.275e-05	-0.32	1.061e-01	-1.76	3.254e-21	-0.24	2.335e-01	-1.68	1.224e-19
✦OG0001050	4.49	28.45	1.43±0.13	1.582e-06	0.85	4.946e-10	-0.58	1.993e-05	1.76	4.490e-40	0.33	1.711e-02
✦OG0001619	4.18	22.41	1.41±0.16	2.090e-05	0.66	1.164e-04	-0.74	1.219e-05	1.31	3.685e-15	-0.09	6.159e-01
✦OG0001237	5.65	8.81	1.40±0.33	7.716e-03	-0.80	2.480e-02	-2.21	8.331e-11	1.49	1.251e-05	0.09	8.107e-01
✦OG0001082	4.48	35.03	1.39±0.12	1.043e-07	0.76	6.451e-09	-0.64	1.013e-06	-0.04	7.862e-01	-1.43	9.249e-30
✦OG0002000	4.75	11.13	1.37±0.27	2.887e-03	1.91	2.831e-11	0.54	7.055e-02	-1.62	1.347e-08	-2.99	9.880e-27
✦OG0000267	6.19	16.99	1.32±0.19	2.190e-04	-0.68	9.156e-04	-2.01	6.029e-24	0.75	2.084e-04	-0.57	5.349e-03
✦OG0001535	5.75	33.53	1.31±0.10	1.846e-07	0.57	5.724e-08	-0.75	3.793e-13	0.83	5.654e-16	-0.49	2.447e-06
✦OG0001880	5.46	34.03	1.29±0.10	1.495e-07	0.90	2.003e-19	-0.39	9.874e-05	0.29	5.164e-03	-1.01	7.243e-25
✦OG0001167	5.03	22.91	1.26±0.14	1.669e-05	0.36	1.997e-02	-0.90	1.200e-09	0.95	1.582e-10	-0.32	3.561e-02
✦OG0001152	6.02	26.37	1.25±0.12	3.776e-06	1.56	1.525e-34	0.32	1.615e-02	0.58	6.338e-06	-0.67	1.407e-07
✦OG0000216	6.35	25.08	1.24±0.13	6.346e-06	-0.06	6.927e-01	-1.30	7.295e-23	-0.29	3.591e-02	-1.53	1.062e-31
✦OG0001886	9.10	36.09	1.24±0.11	6.604e-08	0.33	4.658e-03	-0.92	7.017e-17	0.85	1.579e-14	-0.40	3.449e-04
✦OG0001200	5.10	21.65	1.23±0.15	2.856e-05	-0.03	8.711e-01	-1.26	7.517e-17	1.26	4.753e-17	0.03	8.450e-01
✦OG0001539	5.83	30.18	1.23±0.10	7.698e-07	0.83	3.363e-14	-0.40	3.060e-04	0.05	6.997e-01	-1.18	1.810e-28
✦OG0001105	4.16	19.61	1.22±0.16	7.193e-05	0.32	7.080e-02	-0.90	4.372e-08	1.78	3.244e-28	0.56	6.561e-04
✦OG0001126	5.42	23.05	1.21±0.14	1.565e-05	0.88	7.093e-10	-0.33	2.243e-02	0.29	4.749e-02	-0.92	4.674e-11
✦OG0001721	5.79	39.60	1.20±0.09	1.549e-08	0.83	2.101e-18	-0.37	9.777e-05	-0.43	6.540e-06	-1.63	4.708e-70
✦OG0000918	4.94	16.90	1.19±0.18	2.283e-04	1.30	1.065e-12	0.11	5.870e-01	1.45	8.915e-16	0.26	1.750e-01
✦OG0001378	4.60	29.22	1.19±0.12	1.135e-06	0.24	7.338e-02	-0.95	1.847e-14	1.29	5.885e-26	0.11	4.251e-01
✦OG0001541	5.24	30.24	1.19±0.10	7.671e-07	0.36	8.137e-04	-0.83	2.559e-15	0.92	1.450e-18	-0.28	1.020e-02
✦OG0000871	4.48	16.81	1.18±0.17	2.382e-04	-0.28	1.483e-01	-1.46	4.295e-16	0.57	2.062e-03	-0.61	7.807e-04
✦OG0000680	5.49	22.34	1.18±0.14	2.145e-05	0.39	8.105e-03	-0.79	2.770e-08	0.56	8.909e-05	-0.62	1.279e-05
✦OG0001234	4.54	23.83	1.18±0.14	1.101e-05	0.00	9.828e-01	-1.18	4.338e-17	-1.33	1.518e-21	-2.51	1.332e-74
✦OG0001537	5.28	16.25	1.17±0.18	3.039e-04	0.40	3.926e-02	-0.77	3.583e-05	-0.58	1.922e-03	-1.75	3.446e-22

Orthogroup	LRT				$AL2^T$: 30°C. vs 10°C.		$ALJ2$: 30°C. vs 10°C.		10°C.: $ALJ2$ vs $AL2^T$		30°C.: $ALJ2$ vs $AL2^T$	
	Mean	stat	$b \pm se$	P_{adj}	b	P_{adj}	b	P_{adj}	b	P_{adj}	b	P_{adj}
✦OG0000586	5.40	19.82	1.17±0.15	$6.538e-05$	-0.24	$1.522e-01$	-1.41	$9.157e-20$	0.57	$2.726e-04$	-0.59	$1.582e-04$
✦OG0001177	6.22	29.74	1.17±0.10	$9.058e-07$	-0.42	$1.071e-04$	-1.59	$4.602e-53$	-0.29	$7.906e-03$	-1.46	$1.806e-45$
✦OG0001575	6.58	22.36	1.16±0.13	$2.134e-05$	1.65	$1.309e-32$	0.49	$4.921e-04$	-1.68	$9.271e-35$	-2.84	$9.902e-98$
✦OG0001154	5.43	22.42	1.16±0.13	$2.090e-05$	-0.22	$1.483e-01$	-1.38	$1.368e-23$	0.10	$5.061e-01$	-1.06	$1.077e-14$
✦OG0001753	5.53	26.47	1.15±0.11	$3.640e-06$	-0.62	$1.486e-07$	-1.78	$1.310e-53$	0.86	$1.415e-13$	-0.29	$1.522e-02$
✦OG0000607	5.63	28.13	1.15±0.11	$1.816e-06$	-0.11	$3.745e-01$	-1.26	$5.549e-31$	0.43	$1.187e-04$	-0.72	$3.190e-11$
✦OG0001143	7.64	28.35	1.15±0.10	$1.652e-06$	0.15	$1.977e-01$	-1.00	$2.763e-20$	-0.27	$1.596e-02$	-1.42	$1.822e-40$
✦OG0000813	7.02	27.91	1.14±0.11	$1.939e-06$	0.08	$5.056e-01$	-1.06	$2.613e-22$	0.62	$1.529e-08$	-0.52	$2.358e-06$
✦OG0000287	5.62	17.39	1.13±0.16	$1.845e-04$	0.48	$6.190e-03$	-0.65	$1.379e-04$	1.13	$1.669e-11$	-0.00	$9.969e-01$
✦OG0001863	4.75	24.90	1.13±0.12	$6.869e-06$	-0.35	$8.892e-03$	-1.48	$1.229e-31$	-0.24	$6.950e-02$	-1.38	$6.545e-28$
✦OG0002107	5.01	19.15	1.12±0.15	$8.712e-05$	0.58	$2.078e-04$	-0.53	$6.456e-04$	0.25	$1.192e-01$	-0.87	$1.470e-08$
✦OG0000401	6.23	27.12	1.12±0.11	$2.741e-06$	-0.24	$3.811e-02$	-1.37	$2.109e-35$	0.13	$2.663e-01$	-0.99	$1.772e-19$
✦OG0001179	5.17	34.80	1.11±0.10	$1.135e-07$	0.63	$3.132e-09$	-0.49	$3.765e-06$	0.08	$5.099e-01$	-1.04	$5.280e-24$
✦OG0000413	5.57	13.59	1.10±0.19	$9.940e-04$	1.85	$7.375e-21$	0.76	$1.568e-04$	0.63	$1.873e-03$	-0.47	$1.974e-02$
✦OG0000296	4.87	18.03	1.10±0.15	$1.382e-04$	1.01	$3.158e-10$	-0.09	$6.147e-01$	-0.91	$1.017e-08$	-2.01	$3.503e-38$
✦OG0001107	4.59	28.06	1.10±0.12	$1.846e-06$	0.09	$5.257e-01$	-1.01	$8.745e-16$	1.55	$8.066e-36$	0.45	$3.869e-04$
✦OG0000514	6.84	32.85	1.10±0.09	$2.428e-07$	0.15	$1.330e-01$	-0.95	$2.201e-26$	-0.19	$4.518e-02$	-1.28	$6.118e-48$
✦OG0001985	4.86	28.09	1.09±0.11	$1.840e-06$	0.66	$1.061e-08$	-0.43	$2.383e-04$	-0.32	$6.616e-03$	-1.41	$3.307e-36$
✦OG0000866	6.46	37.18	1.09±0.08	$4.342e-08$	0.45	$3.581e-07$	-0.64	$3.359e-13$	0.39	$8.762e-06$	-0.70	$7.615e-16$
✦OG0001416	5.42	25.29	1.07±0.11	$5.955e-06$	0.06	$6.184e-01$	-1.01	$7.127e-19$	0.22	$6.831e-02$	-0.86	$3.591e-14$
✦OG0001711	4.28	19.75	1.06±0.14	$6.693e-05$	0.85	$3.890e-09$	-0.22	$1.532e-01$	0.39	$7.855e-03$	-0.68	$2.049e-06$
✦OG0001206	5.84	34.29	1.05±0.09	$1.402e-07$	0.54	$6.646e-09$	-0.51	$4.273e-08$	-0.20	$3.294e-02$	-1.26	$7.653e-44$
✦OG0001334	3.81	13.85	1.04±0.18	$8.878e-04$	-0.27	$1.784e-01$	-1.31	$1.207e-12$	0.53	$4.827e-03$	-0.51	$6.899e-03$
✦OG0000682	6.05	26.17	1.04±0.10	$4.128e-06$	0.44	$4.837e-05$	-0.59	$3.037e-08$	0.02	$8.718e-01$	-1.02	$3.358e-22$
✦OG0000785	6.67	6.89	1.03±0.28	$1.714e-02$	2.22	$2.289e-14$	1.19	$4.677e-05$	-1.41	$1.250e-06$	-2.44	$1.212e-17$
✦OG0001546	3.69	17.02	1.01±0.16	$2.155e-04$	1.26	$1.469e-13$	0.25	$1.664e-01$	-0.13	$4.953e-01$	-1.14	$1.162e-11$
