# Supplementary material for: Molecular and Physiological Adaptations to Low Temperature in Thioalkalivibrio Strains Isolated from Soda Lakes with Different Temperature Regimes
Source: mSystems. 2021 Apr 27;6(2):e01202-20. doi: 10.1128/mSystems.01202-20 (PMC8092127; doi:10.1128/mSystems.01202-20)
Supplement: TABLE S7 [file msystems.01202-20-st007.pdf]

	LRT				$AL2^T$: 30°C. vs 10°C.		$ALJ2$: 30°C. vs 10°C.		10°C.: $ALJ2$ vs $AL2^T$		30°C.: $ALJ2$ vs $AL2^T$	
Orthogroup	Mean	stat	$b \pm se$	P_{adj}	b	P_{adj}	b	P_{adj}	b	P_{adj}	b	P_{adj}
✦OG0000880	7.37	31.19	-1.01±0.09	$5.079e-07$	-0.36	$2.872e-04$	0.66	$7.867e-12$	-1.31	$7.594e-44$	-0.30	$2.300e-03$
✦OG0001565	5.99	30.40	-1.01±0.09	$7.139e-07$	0.73	$3.182e-15$	1.74	$4.231e-82$	0.46	$5.691e-07$	1.47	$7.477e-60$
✦OG0001382	6.71	22.72	-1.01±0.12	$1.801e-05$	-0.52	$2.143e-05$	0.49	$4.536e-05$	-0.71	$2.959e-09$	0.30	$1.281e-02$
✦OG0000561	6.05	19.90	-1.01±0.13	$6.393e-05$	-0.19	$1.870e-01$	0.82	$1.213e-09$	0.81	$1.803e-09$	1.81	$1.630e-43$
✦OG0001236	6.47	8.15	-1.01±0.25	$1.018e-02$	-2.40	$9.651e-21$	-1.39	$6.971e-08$	-0.70	$7.846e-03$	0.31	$2.507e-01$
✦OG0000953	6.33	34.02	-1.02±0.09	$1.495e-07$	-0.81	$3.850e-19$	0.21	$2.228e-02$	0.96	$6.340e-27$	1.98	$3.707e-112$
✦OG0001615	6.31	21.80	-1.02±0.12	$2.706e-05$	-0.91	$7.541e-13$	0.12	$3.932e-01$	-0.54	$1.988e-05$	0.48	$1.290e-04$
✦OG0001568	6.09	33.01	-1.04±0.09	$2.291e-07$	-0.68	$4.228e-14$	0.36	$9.932e-05$	-0.62	$3.398e-12$	0.42	$4.129e-06$
✦OG0001478	4.68	27.68	-1.04±0.11	$2.153e-06$	-0.34	$4.717e-03$	0.70	$1.663e-09$	-0.67	$7.504e-09$	0.37	$1.600e-03$
✦OG0000376	5.91	8.18	-1.04±0.26	$1.008e-02$	0.69	$1.297e-02$	1.73	$5.984e-11$	0.08	$7.992e-01$	1.12	$2.356e-05$
✦OG0001850	6.90	31.68	-1.07±0.09	$4.091e-07$	-0.56	$6.423e-10$	0.51	$2.321e-08$	-0.19	$4.518e-02$	0.88	$2.789e-23$
✦OG0001444	7.83	15.78	-1.07±0.17	$3.714e-04$	0.33	$7.163e-02$	1.40	$3.062e-16$	-2.55	$6.691e-52$	-1.48	$1.749e-18$
✦OG0000720	6.70	34.70	-1.08±0.09	$1.173e-07$	-0.36	$9.109e-05$	0.72	$3.174e-16$	0.62	$1.994e-12$	1.71	$6.660e-86$
✦OG0001140	5.70	22.12	-1.08±0.13	$2.372e-05$	0.30	$2.822e-02$	1.38	$1.285e-26$	-0.77	$3.355e-09$	0.31	$2.122e-02$
✦OG0001101	8.48	18.57	-1.08±0.15	$1.126e-04$	-0.28	$8.079e-02$	0.80	$1.989e-07$	-0.62	$5.198e-05$	0.46	$3.043e-03$
✦OG0001476	3.71	11.64	-1.08±0.21	$2.340e-03$	-0.15	$5.385e-01$	0.93	$2.314e-05$	-0.38	$9.606e-02$	0.70	$1.406e-03$
✦OG0000591	7.15	29.90	-1.09±0.09	$8.594e-07$	-0.91	$2.219e-20$	0.19	$6.743e-02$	-0.06	$5.822e-01$	1.03	$5.329e-27$
✦OG0001420	6.96	8.15	-1.09±0.27	$1.018e-02$	-1.03	$2.901e-04$	0.06	$8.520e-01$	-2.10	$1.942e-14$	-1.01	$2.665e-04$
✦OG0000829	8.41	26.99	-1.10±0.11	$2.909e-06$	-0.23	$4.931e-02$	0.87	$1.436e-15$	-0.53	$1.759e-06$	0.57	$1.598e-07$
✦OG0000754	6.51	12.23	-1.10±0.21	$1.798e-03$	0.14	$5.582e-01$	1.24	$7.145e-09$	-0.76	$4.742e-04$	0.34	$1.245e-01$
✦OG0001888	3.74	9.40	-1.10±0.25	$6.007e-03$	-1.19	$4.815e-06$	-0.09	$7.610e-01$	-0.08	$7.788e-01$	1.02	$7.349e-05$
✦OG0001230	3.41	8.55	-1.10±0.26	$8.679e-03$	-0.60	$3.621e-02$	0.50	$7.763e-02$	0.70	$1.176e-02$	1.81	$1.975e-11$
✦OG0002064	5.89	22.93	-1.12±0.13	$1.661e-05$	-0.84	$1.783e-10$	0.27	$4.309e-02$	-0.11	$4.489e-01$	1.01	$5.716e-15$
✦OG0001876	6.42	29.98	-1.14±0.10	$8.422e-07$	-0.21	$4.752e-02$	0.93	$4.237e-20$	-0.06	$5.939e-01$	1.08	$2.942e-27$
✦OG0001191	7.48	31.54	-1.15±0.09	$4.354e-07$	0.02	$8.297e-01$	1.18	$6.117e-34$	1.16	$2.075e-33$	2.32	$8.819e-130$
✦OG0002020	8.28	33.00	-1.15±0.10	$2.291e-07$	-0.60	$2.882e-08$	0.56	$1.780e-07$	-0.64	$1.246e-09$	0.51	$1.537e-06$
✦OG0001703	5.53	25.85	-1.15±0.12	$4.751e-06$	-0.09	$5.052e-01$	1.06	$5.461e-19$	1.33	$3.894e-29$	2.48	$1.389e-99$
✦OG0001948	6.71	17.28	-1.18±0.17	$1.942e-04$	-0.81	$6.466e-06$	0.37	$4.544e-02$	-1.17	$2.663e-11$	0.01	$9.742e-01$
✦OG0001475	2.98	13.10	-1.18±0.22	$1.221e-03$	-0.13	$6.133e-01$	1.05	$6.450e-06$	0.53	$2.653e-02$	1.71	$5.701e-14$
✦OG0000406	5.82	34.34	-1.19±0.09	$1.390e-07$	-0.04	$6.832e-01$	1.15	$3.231e-36$	-0.55	$2.342e-09$	0.64	$2.941e-12$
✦OG0002016	6.75	34.17	-1.19±0.09	$1.430e-07$	-0.07	$4.973e-01$	1.12	$1.795e-35$	-0.42	$4.836e-06$	0.77	$1.083e-17$
✦OG0001655	6.36	35.78	-1.20±0.08	$7.594e-08$	-0.81	$2.348e-20$	0.38	$1.659e-05$	-0.27	$2.710e-03$	0.93	$6.021e-27$
✦OG0001653	6.27	33.59	-1.20±0.09	$1.819e-07$	-0.55	$5.174e-09$	0.64	$6.251e-12$	0.02	$8.215e-01$	1.22	$1.648e-40$
✦OG0001648	5.88	29.00	-1.20±0.11	$1.236e-06$	0.12	$3.375e-01$	1.32	$3.091e-33$	0.70	$3.662e-10$	1.90	$1.303e-68$
✦OG0001674	9.15	19.47	-1.20±0.16	$7.596e-05$	0.01	$9.752e-01$	1.21	$9.942e-14$	-1.29	$1.265e-15$	-0.09	$6.078e-01$
✦OG0001284	5.97	35.45	-1.21±0.09	$8.521e-08$	-0.54	$2.927e-09$	0.66	$2.364e-13$	-0.62	$4.506e-12$	0.58	$9.406e-11$
✦OG0001344	7.16	38.65	-1.22±0.09	$2.233e-08$	0.41	$1.334e-05$	1.64	$1.407e-71$	-1.03	$3.457e-29$	0.19	$4.433e-02$
✦OG0002083	6.31	12.04	-1.24±0.23	$1.949e-03$	0.41	$1.161e-01$	1.65	$1.155e-11$	-0.06	$8.308e-01$	1.18	$1.278e-06$
✦OG0001029	6.73	39.55	-1.26±0.09	$1.549e-08$	-0.80	$5.310e-19$	0.46	$2.670e-07$	0.25	$6.540e-03$	1.51	$4.260e-67$
✦OG0000717	7.50	36.69	-1.26±0.09	$5.231e-08$	-0.94	$7.326e-22$	0.32	$1.518e-03$	-0.63	$1.339e-10$	0.63	$7.474e-11$

Orthogroup	LRT				$AL2^T$: 30°C vs 10°C		$ALJ2$: 30°C vs 10°C		10°C: $ALJ2$ vs $AL2^T$		30°C: $ALJ2$ vs $AL2^T$	
	Mean	stat	b±se	P_{adj}	b	P_{adj}	b	P_{adj}	b	P_{adj}	b	P_{adj}
✦OG0000486	6.84	29.76	-1.26±0.11	9.058e − 07	-0.28	1.995e − 02	0.98	2.507e − 18	-0.16	1.941e − 01	1.10	3.821e − 23
✦OG0001283	6.87	28.05	-1.27±0.12	1.846e − 06	-1.11	8.051e − 20	0.16	2.207e − 01	-0.76	4.424e − 10	0.51	2.756e − 05
✦OG0001484	5.42	32.61	-1.28±0.10	2.703e − 07	-0.74	1.505e − 12	0.54	2.157e − 07	-1.27	2.077e − 35	0.01	9.174e − 01
✦OG0001259	4.91	32.84	-1.28±0.11	2.428e − 07	-0.26	3.215e − 02	1.02	1.341e − 19	0.60	1.171e − 07	1.88	1.187e − 64
✦OG0000673	5.51	36.22	-1.29±0.09	6.285e − 08	-0.31	2.215e − 03	0.99	5.229e − 25	-0.35	3.182e − 04	0.94	3.967e − 23
✦OG0000775	6.20	26.24	-1.29±0.13	4.025e − 06	-0.82	1.007e − 09	0.47	4.536e − 04	0.77	5.305e − 09	2.06	2.503e − 57
✦OG0000403	7.42	32.11	-1.30±0.10	3.448e − 07	0.41	1.845e − 04	1.71	3.215e − 59	-1.34	9.417e − 37	-0.04	7.624e − 01
✦OG0001834	6.16	42.49	-1.32±0.09	5.177e − 09	-0.36	9.992e − 05	0.96	1.375e − 27	-0.45	4.187e − 07	0.87	7.102e − 23
✦OG0001613	7.89	19.88	-1.33±0.17	6.393e − 05	-1.46	2.212e − 16	-0.13	5.086e − 01	-0.57	1.723e − 03	0.76	1.698e − 05
✦OG0001445	6.87	13.36	-1.34±0.24	1.108e − 03	0.14	6.128e − 01	1.48	1.452e − 09	-3.08	1.658e − 37	-1.74	6.832e − 13
✦OG0001677	4.70	29.30	-1.35±0.12	1.106e − 06	-0.40	1.848e − 03	0.95	1.734e − 14	0.66	1.092e − 07	2.01	9.661e − 62
✦OG0000428	7.48	12.33	-1.35±0.25	1.720e − 03	0.52	5.878e − 02	1.87	5.897e − 13	-1.96	3.473e − 14	-0.61	2.094e − 02
✦OG0001643	8.08	39.72	-1.36±0.10	1.500e − 08	-0.72	7.624e − 12	0.64	9.295e − 10	0.02	8.726e − 01	1.38	1.439e − 41
✦OG0001692	8.40	41.02	-1.36±0.10	8.156e − 09	-0.39	4.373e − 04	0.98	3.703e − 20	-0.45	2.700e − 05	0.91	5.729e − 18
✦OG0000667	6.86	27.29	-1.39±0.13	2.546e − 06	-0.43	2.254e − 03	0.96	2.545e − 12	-2.34	1.333e − 68	-0.95	1.819e − 12
✦OG0001772	5.11	29.34	-1.41±0.12	1.096e − 06	-0.37	5.728e − 03	1.04	4.626e − 16	-0.97	3.308e − 14	0.44	7.173e − 04
✦OG0000489	6.79	36.45	-1.42±0.10	5.828e − 08	-0.16	1.292e − 01	1.25	1.108e − 36	0.54	8.530e − 08	1.95	5.050e − 89
✦OG0001614	6.55	25.26	-1.45±0.15	5.955e − 06	-1.36	1.875e − 18	0.09	5.924e − 01	-0.71	4.737e − 06	0.74	1.828e − 06
✦OG0001285	6.89	43.63	-1.54±0.09	3.269e − 09	-0.69	3.469e − 14	0.85	2.461e − 21	0.25	5.911e − 03	1.79	2.870e − 92
✦OG0001030	6.33	48.27	-1.56±0.08	5.855e − 10	-0.70	1.814e − 15	0.86	4.266e − 23	-0.76	1.376e − 18	0.80	2.509e − 20
✦OG0000929	6.73	48.97	-1.59±0.09	4.462e − 10	-0.13	1.781e − 01	1.46	1.739e − 62	0.29	1.536e − 03	1.88	8.060e − 104
✦OG0000485	6.60	30.82	-1.60±0.13	5.962e − 07	-0.54	1.522e − 04	1.06	1.208e − 14	-0.17	2.520e − 01	1.43	6.412e − 26
✦OG0001286	7.30	43.45	-1.63±0.09	3.430e − 09	-0.35	3.000e − 04	1.28	4.715e − 42	0.20	4.109e − 02	1.83	9.304e − 87
✦OG0001024	6.98	38.80	-1.67±0.11	2.120e − 08	-0.21	6.607e − 02	1.46	8.296e − 42	0.16	1.613e − 01	1.83	1.645e − 66
✦OG0001905	5.10	23.28	-1.67±0.19	1.396e − 05	-0.08	7.088e − 01	1.59	1.189e − 16	-2.48	3.274e − 39	-0.81	2.670e − 05
✦OG0001025	6.33	30.85	-1.70±0.14	5.962e − 07	-0.01	9.369e − 01	1.69	3.870e − 31	0.59	6.796e − 05	2.29	1.749e − 57
✦OG0001302	4.21	30.83	-1.71±0.14	5.962e − 07	0.46	2.876e − 03	2.17	4.169e − 50	-1.23	6.554e − 17	0.49	1.141e − 03
✦OG0000721	5.73	33.10	-1.74±0.13	2.238e − 07	0.32	2.767e − 02	2.06	1.343e − 51	0.07	6.367e − 01	1.81	8.538e − 41
✦OG0001201	7.05	31.93	-1.75±0.14	3.651e − 07	0.07	6.533e − 01	1.83	5.264e − 37	0.85	5.159e − 09	2.60	3.529e − 75
✦OG0001011	4.87	25.20	-1.76±0.18	6.081e − 06	-0.94	8.958e − 07	0.82	1.601e − 05	0.48	1.220e − 02	2.25	4.275e − 34
✦OG0002069	8.29	13.97	-1.78±0.30	8.407e − 04	0.53	1.161e − 01	2.32	1.658e − 13	-3.49	2.928e − 29	-1.70	5.733e − 08
✦OG0000536	5.74	7.30	-1.81±0.48	1.441e − 02	-0.60	2.614e − 01	1.21	1.719e − 02	-2.09	2.680e − 05	-0.28	6.111e − 01
✦OG0001028	6.37	41.92	-1.86±0.11	6.192e − 09	-0.97	8.371e − 19	0.89	2.339e − 16	0.17	1.420e − 01	2.03	4.096e − 81
✦OG0001906	4.80	34.25	-1.89±0.14	1.410e − 07	-0.25	1.029e − 01	1.64	2.138e − 30	-2.54	1.398e − 72	-0.65	5.908e − 06
✦OG0000947	7.40	16.46	-1.91±0.29	2.795e − 04	-0.54	8.649e − 02	1.36	5.201e − 06	-1.25	3.104e − 05	0.66	2.978e − 02
✦OG0002096	7.39	37.67	-2.12±0.14	3.447e − 08	-0.26	8.373e − 02	1.85	5.781e − 39	0.80	2.261e − 08	2.92	9.233e − 97
✦OG0001257	6.11	47.20	-2.21±0.10	7.827e − 10	-1.17	3.165e − 27	1.03	1.031e − 21	-0.81	6.398e − 14	1.40	2.455e − 39
✦OG0001591	5.89	38.40	-2.33±0.15	2.484e − 08	-0.47	3.509e − 03	1.87	2.097e − 34	0.22	1.851e − 01	2.55	2.597e − 64
✦OG0000759	8.76	9.40	-2.36±0.53	6.007e − 03	1.07	6.533e − 02	3.43	3.803e − 10	-2.78	3.843e − 07	-0.43	4.704e − 01
✦OG0002056	7.22	46.28	-2.45±0.12	1.022e − 09	-0.77	1.197e − 09	1.69	6.162e − 43	0.38	3.146e − 03	2.83	3.106e − 120
✦OG0000306	6.24	24.58	-2.54±0.27	8.003e − 06	0.82	4.519e − 03	3.35	2.437e − 34	-2.62	1.372e − 21	-0.09	7.803e − 01
✦OG0001649	7.07	41.36	-2.73±0.16	7.486e − 09	-1.04	3.211e − 10	1.69	1.261e − 25	0.06	7.275e − 01	2.79	6.768e − 69
✦OG0001564	6.06	58.14	-2.90±0.10	3.953e − 11	-0.89	7.527e − 19	2.01	4.017e − 93	0.13	2.187e − 01	3.03	5.671e − 213
✦OG0000487	3.78	32.01	-2.91±0.23	3.581e − 07	-1.68	4.511e − 12	1.23	3.927e − 07	-1.09	7.559e − 06	1.82	1.818e − 14
✦OG0002008	4.66	33.74	-3.05±0.23	1.703e − 07	-1.67	3.230e − 12	1.39	6.310e − 09	0.09	7.467e − 01	3.14	1.439e − 41
✦OG0001650	6.00	34.80	-3.10±0.22	1.135e − 07	-1.46	4.663e − 10	1.64	1.644e − 12	0.03	9.172e − 01	3.13	4.789e − 43
✦OG0000488	5.30	35.51	-3.10±0.22	8.416e − 08	-1.71	5.229e − 14	1.38	1.119e − 09	-0.11	6.700e − 01	2.99	2.087e − 41
✦OG0001645	5.98	46.95	-3.11±0.15	7.827e − 10	-1.62	1.020e − 25	1.49	2.546e − 22	-0.73	2.433e − 06	2.38	5.536e − 56

Orthogroup	LRT				$AL2^T$: 30°C. vs 10°C.		$ALJ2$: 30°C. vs 10°C.		10°C.: $ALJ2$ vs $AL2^T$		30°C.: $ALJ2$ vs $AL2^T$	
	Mean	stat	b±se	P_{adj}	b	P_{adj}	b	P_{adj}	b	P_{adj}	b	P_{adj}
✦OG0001644	6.47	37.97	-3.32±0.22	$3.018e-08$	-1.69	$4.212e-14$	1.63	$2.583e-13$	-0.19	$4.268e-01$	3.13	$1.250e-46$
✦OG0001287	5.58	39.89	-3.35±0.20	$1.412e-08$	-1.76	$1.010e-16$	1.59	$3.096e-14$	-0.03	$9.046e-01$	3.32	$1.173e-58$
✦OG0001074	6.84	50.13	-3.37±0.15	$2.892e-10$	-1.31	$3.488e-18$	2.06	$1.612e-43$	-0.42	$7.183e-03$	2.96	$5.643e-90$
✦OG0001590	5.94	35.52	-3.45±0.24	$8.416e-08$	-1.08	$2.864e-05$	2.37	$2.712e-21$	-0.35	$1.881e-01$	3.10	$5.058e-36$
✦OG0001026	6.87	41.22	-3.66±0.21	$7.822e-09$	-1.82	$1.951e-16$	1.84	$3.891e-17$	-0.39	$9.508e-02$	3.27	$2.223e-52$
✦OG0001451	6.14	28.96	-3.70±0.33	$1.252e-06$	-0.12	$7.675e-01$	3.59	$4.357e-26$	-2.91	$1.170e-17$	0.80	$2.307e-02$
✦OG0001563	7.58	56.83	-3.73±0.13	$3.953e-11$	-1.22	$1.909e-19$	2.52	$2.681e-81$	-0.50	$2.698e-04$	3.24	$7.584e-135$
✦OG0001647	7.13	50.06	-3.77±0.16	$2.892e-10$	-0.75	$1.539e-05$	3.02	$3.582e-74$	0.69	$5.827e-05$	4.45	$1.810e-161$
✦OG0001560	8.11	47.90	-3.82±0.18	$6.517e-10$	-1.48	$1.036e-15$	2.34	$5.622e-38$	-0.01	$9.672e-01$	3.81	$1.935e-100$
✦OG0001027	6.02	36.79	-3.87±0.26	$5.071e-08$	-2.12	$6.009e-15$	1.75	$1.114e-10$	-0.23	$4.313e-01$	3.64	$6.482e-43$
✦OG0001258	6.38	56.29	-3.92±0.14	$3.953e-11$	-1.69	$7.431e-32$	2.23	$2.946e-56$	-0.99	$4.994e-12$	2.93	$2.172e-97$
✦OG0000755	6.90	17.24	-3.94±0.57	$1.973e-04$	0.31	$6.437e-01$	4.26	$5.313e-13$	-2.38	$7.078e-05$	1.57	$9.152e-03$
✦OG0000450	7.15	44.39	-3.95±0.21	$2.424e-09$	-1.45	$1.768e-11$	2.50	$2.051e-32$	0.07	$7.869e-01$	4.02	$4.971e-83$
✦OG0001562	8.42	53.72	-4.00±0.15	$8.732e-11$	-1.48	$1.317e-20$	2.51	$4.218e-58$	-0.81	$3.726e-07$	3.19	$8.004e-94$
✦OG0001071	7.08	36.24	-4.01±0.28	$6.285e-08$	-1.42	$1.003e-06$	2.59	$8.194e-20$	0.16	$6.256e-01$	4.17	$2.675e-50$
✦OG0001561	6.94	44.16	-4.05±0.21	$2.597e-09$	-1.11	$6.954e-07$	2.94	$1.584e-41$	0.05	$8.428e-01$	4.10	$3.565e-81$
✦OG0001646	6.60	41.59	-4.10±0.23	$6.924e-09$	-1.90	$7.463e-15$	2.20	$1.053e-19$	-0.74	$3.001e-03$	3.36	$2.891e-45$
✦OG0000451	7.31	46.92	-4.18±0.20	$7.827e-10$	-1.54	$2.118e-13$	2.65	$4.889e-38$	-0.31	$1.558e-01$	3.87	$2.690e-81$
✦OG0001073	6.73	53.75	-4.24±0.16	$8.732e-11$	-0.72	$3.094e-05$	3.52	$1.379e-100$	-0.44	$1.107e-02$	3.80	$1.840e-118$
✦OG0002052	7.67	50.86	-4.24±0.18	$2.677e-10$	-1.31	$2.548e-12$	2.94	$2.005e-58$	-0.16	$4.310e-01$	4.08	$3.037e-113$
✦OG0000452	7.15	47.74	-4.24±0.20	$6.571e-10$	-1.39	$1.597e-11$	2.85	$5.455e-45$	-0.35	$1.033e-01$	3.89	$2.748e-84$
✦OG0001070	6.65	39.01	-4.26±0.27	$1.945e-08$	-1.65	$3.434e-09$	2.61	$1.649e-21$	0.55	$5.597e-02$	4.80	$2.023e-71$
✦OG0001559	7.47	43.12	-4.31±0.23	$3.906e-09$	-1.63	$3.340e-11$	2.68	$6.565e-29$	-0.07	$8.150e-01$	4.24	$1.218e-71$
✦OG0000928	7.38	46.91	-4.35±0.21	$7.827e-10$	-1.25	$1.051e-08$	3.10	$7.390e-48$	0.54	$1.585e-02$	4.89	$5.033e-119$
✦OG0002070	7.07	30.19	-4.36±0.37	$7.698e-07$	-0.49	$2.439e-01$	3.87	$4.287e-24$	-3.10	$6.516e-16$	1.27	$1.154e-03$
