# Supplementary material for: Molecular and Physiological Adaptations to Low Temperature in Thioalkalivibrio Strains Isolated from Soda Lakes with Different Temperature Regimes
Source: mSystems. 2021 Apr 27;6(2):e01202-20. doi: 10.1128/mSystems.01202-20 (PMC8092127; doi:10.1128/mSystems.01202-20)
Supplement: TABLE S8 [file msystems.01202-20-st008.pdf]

Orthogroup	$AL2^T$ protein ID	$AL2^T$ locus tag	$ALJ2$ protein ID	$ALJ2$ locus tag	GO, PFAM and IPR ID's	Protein product
OG0000216	WP_012982660.1	B0684_RS10160	WP_012982660.1	F468_RS0108435	GO:0003723, GO:0003743, GO:0006413, PF01176, IPR006196	translation initiation factor IF-1
OG0000217	WP_012983953.1	B0684_RS07755	WP_012983953.1	F468_RS0100780	GO:0015078, GO:0033177, GO:1902600, PF00137, IPR002379	F0F1 ATP synthase subunit C
OG0000224	WP_017941141.1	B0684_RS06715	WP_017941141.1	F468_RS0104125	PF13183, IPR017896	4Fe-4S dicluster domain-containing protein
OG0000225	WP_017941410.1	B0684_RS07100	WP_012983362.1	F468_RS0105920	GO:0003735, GO:0005840, GO:0006412, PF00471, IPR001705	50S ribosomal protein L33
OG0000254	WP_018143948.1	B0684_RS07030	WP_012981989.1	F468_RS0103800	GO:0003677, PF00216, IPR000119	HU family DNA-binding protein
OG0000266	WP_018144009.1	B0684_RS06710	WP_012982044.1	F468_RS0104120	PF01206, IPR001455	sulfurtransferase TusA family protein
OG0000267	WP_018144010.1	B0684_RS06705	WP_018649036.1	F468_RS0104115	PF00581, IPR001763	rhodanese-like domain-containing protein
OG0000283	WP_018144105.1	B0684_RS10830	WP_018994637.1	F468_RS0103615	GO:0003735, GO:0005840, GO:0006412, PF00380, IPR000754	30S ribosomal protein S9
OG0000284	WP_018144106.1	B0684_RS10825	WP_018138548.1	F468_RS0103610	GO:0003735, GO:0005840, GO:0006412, PF00572, IPR005822	50S ribosomal protein L13
OG0000287	WP_018144141.1	B0684_RS10645	WP_018138609.1	F468_RS0105695	GO:0004068, GO:0006523, PF02261, IPR003190	aspartate 1-decarboxylase
OG0000293	WP_018144175.1	B0684_RS04585	WP_018137751.1	F468_RS0107165	GO:0003677, GO:0032784, PF01272, PF14760, IPR001437, IPR029462	nucleoside diphosphate kinase regulator
OG0000296	WP_018144197.1	B0684_RS04475	WP_018139171.1	F468_RS0111870	PF00383, IPR002125	nucleoside deaminase
OG0000297	WP_018144213.1	B0684_RS04395	WP_018138874.1	F468_RS0111495	PF09996, IPR018714	DUF2237 domain-containing protein
OG0000306	WP_018144418.1	B0684_RS03415	WP_018137713.1	F468_RS0107460		hypothetical protein;methyl-accepting chemotaxis protein
OG0000309	WP_018144475.1	B0684_RS04885	WP_018174848.1	F468_RS0110370	PF02381, IPR020603	transcriptional regulator MraZ
OG0000327	WP_018144556.1	B0684_RS09260	WP_018175241.1	F468_RS0102180		hypothetical protein
OG0000343	WP_018144698.1	B0684_RS10560	WP_018995051.1	F468_RS0109780	PF03625, IPR005180	DUF302 domain-containing protein
OG0000344	WP_018144712.1	B0684_RS10490	WP_012983699.1	F468_RS0109745		hypothetical protein
OG0000351	WP_018144765.1	B0684_RS13565	WP_018139460.1	F468_RS0109190	PF01135	protein-L-isoaspartate O-methyltransferase
OG0000353	WP_018144849.1	B0684_RS13160	WP_012983857.1	F468_RS0100220	GO:0015159, GO:0015774, GO:0016020, PF02563, IPR003715	sugar ABC transporter substrate-binding protein
OG0000354	WP_018144868.1	B0684_RS13205	WP_018139470.1	F468_RS0100140	PF02597, IPR003749	molybdopterin converting factor subunit 1
OG0000360	WP_018144912.1	B0684_RS07540	WP_012981443.1	F468_RS0101000	GO:0016787, PF01546, PF07687, IPR002933, IPR011650	Zn-dependent hydrolase
OG0000364	WP_018144928.1	B0684_RS07620	WP_018994490.1	F468_RS0101485	GO:0000105, GO:0004424, PF00475, IPR000807	imidazoleglycerol-phosphate dehydratase HisB
OG0000369	WP_018144945.1	B0684_RS07710	WP_018139732.1	F468_RS0100735		hypothetical protein
OG0000374	WP_018144985.1	B0684_RS02700	WP_018994493.1	F468_RS0101545	PF17319, IPR035287	hypothetical protein

Orthogroup	$AL2^T$ protein ID	$AL2^T$ locus tag	$ALJ2$ protein ID	$ALJ2$ locus tag	GO, PFAM and IPR ID's	Protein product
OG0000375	WP_018144999.1	B0684_RS02770	WP_018994471.1	F468_RS0101275		DNA-binding response regulator;hypothetical protein
OG0000376	WP_018145016.1	B0684_RS02865	WP_018994481.1	F468_RS0101350		hypothetical protein
OG0000383	WP_018145076.1	B0684_RS03155	WP_012981566.1	F468_RS0101630	PF04380, IPR007475	hypothetical protein
OG0000385	WP_018145090.1	B0684_RS03225	WP_018138224.1	F468_RS0101700	PF03597, IPR004714	cbb3-type cytochrome oxidase assembly protein CcoS
OG0000397	WP_018145150.1	B0684_RS12670	WP_018137886.1	F468_RS0109605	GO:0009055, GO:0020037, PF13442, IPR009056	cytochrome c
OG0000401	WP_018145175.1	B0684_RS12725	WP_012983717.1	F468_RS0109655	GO:0004014, GO:0008295, PF02675, IPR003826	adenosylmethionine decarboxylase
OG0000403	WP_018145185.1	B0684_RS12780	WP_012983665.1	F468_RS0109905	GO:0003677, GO:0003700, GO:0006352, GO:0006355, GO:0016987, PF04542, PF08281, IPR007627, IPR013249	RNA polymerase subunit sigma-24;RNA polymerase sigma factor
OG0000404	WP_018145193.1	B0684_RS12820	WP_018174901.1	F468_RS0109950	PF02643, IPR003795	DUF192 domain-containing protein
OG0000405	WP_018145194.1	B0684_RS12825	WP_012983655.1	F468_RS0109955	GO:0030001, GO:0046872, PF00403, IPR006121	heavy metal transporter
OG0000406	WP_018145208.1	B0684_RS11625	WP_018139056.1	F468_RS0102320	PF13098, IPR012336	thioredoxin
OG0000407	WP_018145210.1	B0684_RS11635	WP_018139058.1	F468_RS0102330	GO:0006457, PF00166, IPR020818	co-chaperone GroES
OG0000413	WP_018145252.1	B0684_RS11790	WP_018137953.1	F468_RS0102560		hypothetical protein
OG0000421	WP_018145301.1	B0684_RS11435	WP_012981824.1	F468_RS0102960	PF13740	glycine cleavage system protein R
OG0000428	WP_018145335.1	B0684_RS03720	WP_018137668.1	F468_RS0103155		hypothetical protein
OG0000439	WP_018145403.1	B0684_RS00485	WP_012983058.1	F468_RS0111285		hypothetical protein
OG0000450	WP_018145467.1	B0684_RS00840	WP_018139143.1	F468_RS0112030	PF06429, IPR010930	flagellar basal-body rod protein FlgF
OG0000451	WP_018145468.1	B0684_RS00845	WP_018139142.1	F468_RS0112035	GO:0071973, PF00460, PF06429, IPR001444, IPR010930	flagellar basal-body rod protein FlgG
OG0000452	WP_018145469.1	B0684_RS00850	WP_018139141.1	F468_RS0112040	GO:0003774, GO:0009427, GO:0071973, PF02107, IPR000527	flagellar basal body L-ring protein FlgH
OG0000461	WP_018145553.1	B0684_RS08115	WP_018173981.1	F468_RS0113155		globin
OG0000463	WP_018145571.1	B0684_RS01090	WP_018139614.1	F468_RS0113245	PF00557, IPR000994	type I methionyl aminopeptidase
OG0000473	WP_018145638.1	B0684_RS01470	WP_018995297.1	F468_RS0112525	PF02082, IPR000944	transcriptional regulator
OG0000485	WP_018145737.1	B0684_RS01615	WP_018139241.1	F468_RS0108770	PF01618, IPR002898	flagellar motor protein
OG0000486	WP_018145741.1	B0684_RS01635	WP_018139237.1	F468_RS0108790	GO:0000160, PF00072, IPR001789	chemotaxis protein CheY
OG0000487	WP_018145748.1	B0684_RS01670	WP_012982568.1	F468_RS0108825	GO:0009306, GO:0016020, PF01313, IPR002191	flagellar biosynthetic protein FliQ
OG0000488	WP_018145749.1	B0684_RS01675	WP_018139231.1	F468_RS0108830	GO:0009306, GO:0016020, PF00813, IPR005838	flagellar biosynthetic protein FliP
OG0000489	WP_018145756.1	B0684_RS01710	WP_018174305.1	F468_RS0108865	GO:0005524, PF00006, PF18269, IPR000194, IPR040627	FliI/YscN family ATPase
OG0000491	WP_018145769.1	B0684_RS01840	WP_018139212.1	F468_RS0108925	GO:0004332, GO:0006096, PF00274, IPR000741	fructose-bisphosphate aldolase class I
OG0000499	WP_018145833.1	B0684_RS02180	WP_018139379.1	F468_RS0108230	PF02699, IPR003849	preprotein translocase subunit YajC
OG0000500	WP_018145857.1	B0684_RS02310	WP_018139346.1	F468_RS0108055		hypothetical protein
OG0000503	WP_018145863.1	B0684_RS02340	WP_018139340.1	F468_RS0108025	GO:0003700, GO:0006355, PF00126, PF03466, IPR000847, IPR005119	LysR family transcriptional regulator

Orthogroup	$AL2^T$ protein ID	$AL2^T$ locus tag	$ALJ2$ protein ID	$ALJ2$ locus tag	GO, PFAM and IPR ID's	Protein product
OG0000504	WP_018145889.1	B0684_RS02500	WP_018994861.1	F468_RS0107390	GO:0007165, GO:0016020, GO:0016021, PF00015, PF00672, IPR003660, IPR004089	methyl-accepting chemotaxis protein
OG0000510	WP_018145981.1	B0684_RS06095	WP_018138258.1	F468_RS0104625	PF00334, IPR034907	nucleoside-diphosphate kinase
OG0000511	WP_018145987.1	B0684_RS06065	WP_018138884.1	F468_RS0111445	GO:0005506, GO:0016226, GO:0051536, PF01592, IPR002871	Fe-S cluster assembly scaffold IscU
OG0000513	WP_018145998.1	B0684_RS06010	WP_018138735.1	F468_RS0105065		hypothetical protein
OG0000514	WP_018146032.1	B0684_RS05820	WP_018138389.1	F468_RS0106945	PF01883, PF10609, IPR002744, IPR033756	iron-sulfur cluster carrier protein ApbC
OG0000519	WP_018146058.1	B0684_RS05690	WP_017925939.1	F468_RS0104745		hypothetical protein
OG0000525	WP_018146069.1	B0684_RS05635	WP_012982157.1	F468_RS0104690	PF07977, IPR013114	3-hydroxyacyl-[acyl-carrier-protein] dehydratase FabA
OG0000529	WP_018146120.1	B0684_RS05365	WP_018175465.1	F468_RS0106405	PF00581, IPR001763	rhodanese-like domain-containing protein;sulfurtransferase
OG0000530	WP_018146122.1	B0684_RS05355	WP_026296502.1	F468_RS0106395	GO:0005887, GO:0008961, GO:0042158, PF01790, IPR001640	prolipoprotein diacylglyceryl transferase
OG0000535	WP_018146152.1	B0684_RS12060	WP_012983227.1	F468_RS14140	GO:0005886, PF07043, IPR009760	DUF1328 domain-containing protein
OG0000536	WP_018146154.1	B0684_RS12050	WP_018138481.1	F468_RS0106595	PF13937, IPR019886	DUF4212 domain-containing protein
OG0000537	WP_018146175.1	B0684_RS11325	WP_018137876.1	F468_RS0111095	PF00581, IPR001763	hypothetical protein
OG0000539	WP_018146209.1	B0684_RS11140	WP_018995195.1	F468_RS0111215	PF10116, IPR019291	host attachment protein
OG0000555	WP_018146304.1	B0684_RS09580	WP_018138640.1	F468_RS0105885	PF00625, IPR008145	guanylate kinase
OG0000561	WP_018146435.1	B0684_RS12415	WP_018138188.1	F468_RS0105250	GO:0003697, PF00436, IPR000424	single-stranded DNA-binding protein
OG0000563	WP_018146440.1	B0684_RS12440	WP_012983508.1	F468_RS0105225	GO:0003723, GO:0019843, PF00163, PF01479, IPR001912, IPR002942	30S ribosomal protein S4
OG0000586	WP_018167891.1	B0684_RS11090	WP_018994681.1	F468_RS0104605	GO:0001522, GO:0003723, GO:0006396, GO:0009451, GO:0009982, PF01509, PF09157, PF16198, IPR002501, IPR015240, IPR032819	tRNA pseudouridine(55) synthase TruB
OG0000591	WP_018167897.1	B0684_RS11050	WP_018174736.1	F468_RS0104565	GO:0006120, GO:0055114, PF00361, PF01059, IPR000260, IPR001750	NADH-quinone oxidoreductase subunit M
OG0000600	WP_018167914.1	B0684_RS02610	WP_038038657.1	F468_RS0104880	PF01812, IPR002698	5-formyltetrahydrofolate cyclo-ligase
OG0000607	WP_018167932.1	B0684_RS09295	WP_012982076.1	F468_RS0104280	PF02635, IPR003787	DsrE family protein
OG0000616	WP_018168013.1	B0684_RS06230	WP_018862951.1	F468_RS0105560		hypothetical protein
OG0000622	WP_018168072.1	B0684_RS13470	WP_018994745.1	F468_RS0105655	GO:0005515, PF13410, PF13417, IPR004045	glutathione S-transferase family protein
OG0000626	WP_018168086.1	B0684_RS10690	WP_018994176.1	F468_RS0111075	PF07883, IPR013096	cupin domain-containing protein
OG0000632	WP_018168112.1	B0684_RS10865	WP_018138812.1	F468_RS0103645	PF11146, IPR021320	DUF2905 domain-containing protein
OG0000633	WP_018168121.1	B0684_RS13100	WP_018138664.1	F468_RS0106035	GO:0008658, PF00905, PF03717, IPR001460, IPR005311	penicillin-binding protein 2
OG0000656	WP_018168179.1	B0684_RS09810	WP_018174554.1	F468_RS0105410	GO:0005525, GO:0006777, PF03205, IPR004435	molybdopterin-guanine dinucleotide biosynthesis protein B
OG0000661	WP_018168187.1	B0684_RS12330	WP_018864759.1	F468_RS0105370	GO:0003677, GO:0003887, GO:0006260, PF04364, IPR007459	DNA polymerase III subunit chi
OG0000667	WP_018168196.1	B0684_RS12385	WP_018138182.1	F468_RS0105280	PF07295, IPR009912	hypothetical protein
OG0000673	WP_018168216.1	B0684_RS03935	WP_018995107.1	F468_RS0110485	GO:0004140, GO:0005524, GO:0015937, PF01121, IPR001977	dephospho-CoA kinase

Orthogroup	$AL2^T$ protein ID	$AL2^T$ locus tag	$ALJ2$ protein ID	$ALJ2$ locus tag	GO, PFAM and IPR ID's	Protein product
OG0000680	WP_018168226.1	B0684_RS04830	WP_018995103.1	F468_RS0110425	GO:0008762, GO:0016491, GO:0050660, GO:0055114, PF01565, PF02873, IPR006094, IPR011601	UDP-N-acetylmuramate dehydrogenase;UDP-N-acetylenolpyruvoylglucosamine reductase
OG0000682	WP_018168229.1	B0684_RS04850	WP_018993163.1	F468_RS0110405	GO:0005524, GO:0009058, GO:0016874, PF02875, PF08245, IPR004101, IPR013221	UDP-N-acetylmuramoyl-L-alanine–D-glutamate ligase
OG0000690	WP_018168245.1	B0684_RS04955	WP_018995091.1	F468_RS0110305	GO:0005507, GO:0042597, GO:0046688, PF04234, IPR007348	hypothetical protein;copper resistance protein CopC
OG0000691	WP_018168247.1	B0684_RS04965	WP_018995089.1	F468_RS0110295	GO:0015562, GO:0055085, PF02321, IPR003423	transporter;TolC family protein
OG0000712	WP_018168331.1	B0684_RS09035	WP_018175182.1	F468_RS0101990		hypothetical protein
OG0000714	WP_018168358.1	B0684_RS08840	WP_018138932.1	F468_RS0110070	PF14334, IPR025500	DUF4390 domain-containing protein
OG0000717	WP_018168362.1	B0684_RS08820	WP_012983636.1	F468_RS0110050	PF01476, IPR018392	LysM peptidoglycan-binding domain-containing protein;LysM domain-containing protein
OG0000720	WP_018168391.1	B0684_RS08675	WP_018175158.1	F468_RS0101800	PF00696, IPR001048	acetylglutamate kinase
OG0000721	WP_018168393.1	B0684_RS08665	WP_018994513.1	F468_RS0101790	GO:0016021, PF01925, IPR002781	sulfite exporter TauE/SafE family protein
OG0000729	WP_018168414.1	B0684_RS06725	WP_018138342.1	F468_RS0104135	GO:0016491, GO:0055114, PF07992, IPR023753	CoB–CoM heterodisulfide reductase iron-sulfur subunit A family protein
OG0000734	WP_018168436.1	B0684_RS06890	WP_026182202.1	F468_RS0106995	GO:0008080, PF00583, IPR000182	ribosomal-protein-alanine N-acetyltransferase
OG0000745	WP_018168485.1	B0684_RS07230	WP_018174644.1	F468_RS0103690	PF00308, IPR013317	DnaA regulatory inactivator Hda
OG0000750	WP_018168504.1	B0684_RS03580	WP_018138363.1	F468_RS0106805		hypothetical protein
OG0000751	WP_018168511.1	B0684_RS03540	WP_018174145.1	F468_RS0106855	GO:0008080, PF13673, IPR000182	GNAT family N-acetyltransferase
OG0000753	WP_018168526.1	B0684_RS03465	WP_018138691.1	F468_RS0104820		hypothetical protein
OG0000754	WP_018168527.1	B0684_RS03460	WP_018138694.1	F468_RS0104835		hypothetical protein
OG0000755	WP_018168529.1	B0684_RS03450	WP_051080571.1	F468_RS0107430	PF16576, IPR032317	efflux RND transporter periplasmic adaptor subunit
OG0000759	WP_018168565.1	B0684_RS12250	WP_018138298.1	F468_RS0104385	GO:0016491, GO:0030151, GO:0042128, GO:0055114, PF00174, PF03404, IPR000572, IPR005066	hypothetical protein;sulfite oxidase
OG0000762	WP_018168575.1	B0684_RS12295	WP_018994676.1	F468_RS0104425	PF01566, IPR001046	divalent metal cation transporter
OG0000767	WP_018168588.1	B0684_RS08265	WP_018138853.1	F468_RS0111600	GO:0003677, PF00440, IPR001647	TetR/AcrR family transcriptional regulator
OG0000774	WP_018168600.1	B0684_RS08330	WP_018138845.1	F468_RS0111670	PF01656, PF13614, IPR002586, IPR025669	chromosome partitioning protein ParA
OG0000775	WP_018168601.1	B0684_RS08335	WP_018138844.1	F468_RS0111675		hypothetical protein
OG0000781	WP_018168646.1	B0684_RS08590	WP_018995229.1	F468_RS0111575	GO:0016020, GO:0055114, PF02322, IPR003317	cytochrome d ubiquinol oxidase subunit II
OG0000784	WP_018168660.1	B0684_RS13155	WP_012983858.1	F468_RS0100225	PF02397, PF13727, IPR003362	hypothetical protein;UDP-phosphate galactose phosphotransferase
OG0000785	WP_018168675.1	B0684_RS07365	WP_026296471.1	F468_RS0100120	PF00011, IPR002068	Hsp20/alpha crystallin family protein
OG0000789	WP_018168758.1	B0684_RS07515	WP_018175062.1	F468_RS0100705	GO:0004356, GO:0006542, GO:0006807, PF00120, PF03951, IPR008146, IPR008147	glutamate–ammonia ligase
OG0000793	WP_018168769.1	B0684_RS07570	WP_018139800.1	F468_RS0101220	GO:0009116, PF00156, IPR000836	orotate phosphoribosyltransferase
OG0000808	WP_018168803.1	B0684_RS07835	WP_018994444.1	F468_RS0100855		hypothetical protein
OG0000813	WP_018168817.1	B0684_RS02685	WP_012981556.1	F468_RS0101580	PF01865, IPR018445	TIGR00153 family protein

Orthogroup	$AL2^T$ protein ID	$AL2^T$ locus tag	$ALJ2$ protein ID	$ALJ2$ locus tag	GO, PFAM and IPR ID's	Protein product
OG0000820	WP_018168839.1	B0684_RS02805	WP_018994476.1	F468_RS0101310	GO:0051536, GO:0055114, PF01058, IPR006137	oxidoreductase
OG0000822	WP_018168857.1	B0684_RS02890	WP_012981508.1	F468_RS0101375	PF00037, PF12838, IPR017896	ferredoxin;4Fe-4S dicluster domain-containing protein
OG0000829	WP_018168873.1	B0684_RS03010	WP_012981454.1	F468_RS0101055	GO:0017004, GO:0020037, PF01578, IPR002541	c-type cytochrome biogenesis protein CcsB
OG0000851	WP_018168925.1	B0684_RS03310	WP_038038511.1	F468_RS0109565	GO:0001522, GO:0003723, GO:0009451, GO:0009982, PF00849, IPR006145	ribosomal large subunit pseudouridine synthase E;pseudouridine synthase
OG0000857	WP_018168934.1	B0684_RS13305	WP_018993249.1	F468_RS0109400	GO:0006364, PF03652, IPR005227	Holliday junction resolvase RuvX
OG0000866	WP_018168961.1	B0684_RS12705	WP_018995044.1	F468_RS0109635	GO:0016757, PF00591, PF02885, IPR000312, IPR017459	anthranilate phosphoribosyltransferase
OG0000871	WP_018168968.1	B0684_RS12760	WP_018138909.1	F468_RS0109880		hypothetical protein
OG0000879	WP_018168985.1	B0684_RS12870	WP_018993467.1	F468_RS0102295	GO:0016209, GO:0016491, GO:0055114, PF00578, IPR000866	TlpA family protein disulfide reductase
OG0000880	WP_018168986.1	B0684_RS12875	WP_018994557.1	F468_RS0102300	GO:0016020, GO:0017004, GO:0055114, PF02683, PF11412, PF13899, IPR003834, IPR028250	protein-disulfide reductase DsbD
OG0000897	WP_018169058.1	B0684_RS11540	WP_018137840.1	F468_RS0102855		hypothetical protein
OG0000915	WP_018169147.1	B0684_RS03795	WP_018994610.1	F468_RS0103230	GO:0000166, GO:0004812, GO:0005524, GO:0006418, PF00152, IPR004364	EF-P lysine aminoacylase GenX
OG0000917	WP_018169159.1	B0684_RS03715	WP_018137667.1	F468_RS0103150		hypothetical protein
OG0000918	WP_018169160.1	B0684_RS03705	WP_018137665.1	F468_RS0103140		hypothetical protein
OG0000924	WP_018169183.1	B0684_RS10575	WP_018995053.1	F468_RS0109795	GO:0003700, GO:0006355, GO:0043565, PF12833, IPR018060	AraC family transcriptional regulator
OG0000925	WP_018169184.1	B0684_RS10570	WP_018995052.1	F468_RS0109790	PF13847, IPR025714	class I SAM-dependent methyltransferase
OG0000928	WP_018169199.1	B0684_RS10480	WP_018137911.1	F468_RS0109735	PF00990, IPR000160	GGDEF domain-containing protein
OG0000929	WP_018169200.1	B0684_RS10475	WP_018995047.1	F468_RS0109730	GO:0003700, GO:0006355, PF00126, PF03466, IPR000847, IPR005119	hydrogen peroxide-inducible genes activator
OG0000932	WP_018169217.1	B0684_RS10385	WP_018175153.1	F468_RS0101770	PF13649, IPR041698	class I SAM-dependent methyltransferase
OG0000940	WP_018169266.1	B0684_RS00100	WP_026296475.1	F468_RS0100550	PF03023, IPR004268	murein biosynthesis integral membrane protein MurJ
OG0000942	WP_018169317.1	B0684_RS00370	WP_018994388.1	F468_RS0100290	PF13401, IPR003593	hypothetical protein;NACHT domain-containing protein
OG0000943	WP_018169333.1	B0684_RS11275	WP_018137870.1	F468_RS0111125	PF01741, IPR037673	large-conductance mechanosensitive channel protein MscL
OG0000945	WP_018169342.1	B0684_RS11320	WP_018995191.1	F468_RS0111150	GO:0009916, GO:0055114, PF01786, IPR002680	oxidase
OG0000946	WP_018169343.1	B0684_RS11330	WP_018994894.1	F468_RS0107660		hypothetical protein
OG0000947	WP_018169356.1	B0684_RS12045	WP_018138480.1	F468_RS0106590	GO:0016020, GO:0022857, GO:0055085, PF00474, IPR001734	cation acetate symporter
OG0000953	WP_018169368.1	B0684_RS12130	WP_018138496.1	F468_RS0106680	PF00929, IPR013520	oligoribonuclease
OG0000956	WP_018169385.1	B0684_RS05370	WP_018175466.1	F468_RS0106410	PF13444	GNAT family N-acetyltransferase
OG0000966	WP_018169409.1	B0684_RS05490	WP_018138871.1	F468_RS0111510	PF05982, IPR010293	sodium-dependent bicarbonate transport family permease
OG0000967	WP_018169410.1	B0684_RS05495	WP_018995220.1	F468_RS0111515		hypothetical protein

Orthogroup	$AL2^T$ protein ID	$AL2^T$ locus tag	$ALJ2$ protein ID	$ALJ2$ locus tag	GO, PFAM and IPR ID's	Protein product
OG0000977	WP_018169451.1	B0684_RS05770	WP_012983101.1	F468_RS0107220		hypothetical protein
OG0000981	WP_018169456.1	B0684_RS05805	WP_018994825.1	F468_RS0106930		hypothetical protein
OG0000988	WP_018169470.1	B0684_RS05880	WP_018174775.1	F468_RS0104935	PF01618, IPR002898	flagellar motor protein MotA
OG0000989	WP_018169471.1	B0684_RS05885	WP_018138715.1	F468_RS0104940	PF09919, IPR018676	hypothetical protein;DUF2149 domain-containing protein
OG0000991	WP_018169473.1	B0684_RS05895	WP_018994699.1	F468_RS0104950	GO:0003824, GO:0009236, PF01656, PF07685, IPR002586, IPR011698	cobyrinate a,c-diamide synthase
OG0000992	WP_018169474.1	B0684_RS05900	WP_051080568.1	F468_RS0104955	GO:0008168, PF00590, IPR000878	uroporphyrinogen-III C-methyltransferase
OG0000993	WP_018169475.1	B0684_RS05905	WP_026296496.1	F468_RS0104960	GO:0008168, GO:0009236, PF01888, IPR002748	cobalt-precorrin-5B (C(1))-methyltransferase
OG0000994	WP_018169478.1	B0684_RS05920	WP_018994703.1	F468_RS0104975	GO:0016491, GO:0055114, PF03460, IPR005117	precorrin-3B synthase
OG0000995	WP_018169479.1	B0684_RS05925	WP_018994704.1	F468_RS0104980	GO:0009236, GO:0016993, PF02570, IPR003722	precorrin-8X methylmutase
OG0001000	WP_018169486.1	B0684_RS05960	WP_012982222.1	F468_RS0105015	GO:0005506, GO:0009055, GO:0020037, PF01322, IPR002321	hypothetical protein
OG0001001	WP_018169490.1	B0684_RS05975	WP_018138731.1	F468_RS0105030	PF14347, IPR025512	DUF4399 domain-containing protein
OG0001009	WP_018169561.1	B0684_RS04800	WP_018138473.1	F468_RS0106555		hypothetical protein
OG0001011	WP_018169568.1	B0684_RS02520	WP_018137695.1	F468_RS0107365	PF03447, IPR005106	hypothetical protein
OG0001014	WP_018169587.1	B0684_RS02415	WP_018993271.1	F468_RS0109215	GO:0003824, GO:0030151, GO:0030170, PF03473, IPR005302	MOSC domain-containing protein
OG0001018	WP_018169631.1	B0684_RS02170	WP_018139381.1	F468_RS0108240	PF02355, PF07549, IPR022646, IPR022813	protein translocase subunit SecF
OG0001019	WP_018169632.1	B0684_RS02165	WP_026278833.1	F468_RS0108245	PF03625, IPR005180	DUF302 domain-containing protein
OG0001024	WP_018169717.1	B0684_RS01720	WP_018862905.1	F468_RS0108875	PF01706, PF14841, PF14842, IPR023087, IPR028263, IPR032779	flagellar motor switch protein FliG
OG0001025	WP_018169718.1	B0684_RS01715	WP_018139223.1	F468_RS0108870	PF02108, IPR018035	hypothetical protein
OG0001026	WP_018169722.1	B0684_RS01690	WP_018139228.1	F468_RS0108845	GO:0003774, GO:0009425, GO:0071973, PF01052, PF02154, IPR001543, IPR001689	flagellar motor switch protein FliM
OG0001027	WP_018169723.1	B0684_RS01685	WP_018139229.1	F468_RS0108840	PF01052, IPR001543	flagellar motor switch protein FliN
OG0001028	WP_018169731.1	B0684_RS01640	WP_018994974.1	F468_RS0108795	GO:0003700, GO:0006352, GO:0006355, PF04539, PF04542, PF04545, IPR007624, IPR007627, IPR007630	RNA polymerase sigma factor FliA
OG0001029	WP_018169734.1	B0684_RS01610	WP_018174317.1	F468_RS0108765	PF00691, PF13677, IPR006665, IPR025713	flagellar motor protein MotB
OG0001030	WP_018169735.1	B0684_RS01605	WP_018993940.1	F468_RS0108760	PF13614, IPR025669	ParA family protein
OG0001037	WP_018169760.1	B0684_RS10010	WP_018993920.1	F468_RS0108590	GO:0004590, GO:0006207, PF00215, IPR001754	orotidine-5'-phosphate decarboxylase
OG0001050	WP_018169817.1	B0684_RS01450	WP_018139676.1	F468_RS0112505	GO:0003824, GO:0051536, PF04055, IPR007197	radical SAM protein
OG0001054	WP_018169854.1	B0684_RS01210	WP_051080581.1	F468_RS0113365	PF00990, IPR000160	GGDEF domain-containing protein
OG0001056	WP_018169856.1	B0684_RS01195	WP_018173955.1	F468_RS0113350	PF00497, IPR001638	hypothetical protein;ABC transporter substrate-binding protein
OG0001061	WP_018169875.1	B0684_RS08180	WP_018139607.1	F468_RS0113210	GO:0016491, PF03358, IPR005025	NAD(P)H:quinone oxidoreductase
OG0001070	WP_018169962.1	B0684_RS00825	WP_018139146.1	F468_RS0112015	GO:0071973, PF00460, PF06429, IPR001444, IPR010930	flagellar basal body rod protein FlgC
OG0001071	WP_018169963.1	B0684_RS00815	WP_012982336.1	F468_RS0112010	GO:0071973, PF00460, IPR001444	flagellar basal body rod protein FlgB

Orthogroup	$AL2^T$ protein ID	$AL2^T$ locus tag	$ALJ2$ protein ID	$ALJ2$ locus tag	GO, PFAM and IPR ID's	Protein product
OG0001073	WP_018169965.1	B0684_RS00800	WP_018995250.1	F468_RS0111995	PF13144, PF17656, IPR017585, IPR041231	flagella basal body P-ring formation protein FlgA
OG0001074	WP_018169967.1	B0684_RS00790	WP_018139151.1	F468_RS0111985	GO:0005515, GO:0044780, PF05130, IPR007809	flagellar protein FlgN
OG0001075	WP_018169973.1	B0684_RS00735	WP_038038701.1	F468_RS0111930	PF00753, IPR001279	MBL fold metallo-hydrolase
OG0001081	WP_018170069.1	B0684_RS04445	WP_018649456.1	F468_RS0107730	GO:0009116, PF00156, IPR000836	phosphoribosyltransferase
OG0001082	WP_018170075.1	B0684_RS04415	WP_026278829.1	F468_RS0107955	GO:0004197, GO:0016020, GO:0071586, PF02517, IPR003675	CPBP family intramembrane metalloprotease
OG0001084	WP_018170091.1	B0684_RS04320	WP_018994892.1	F468_RS0107640	GO:0005515, PF00989, PF00990, PF01590, PF08448, PF13185, IPR000160, IPR003018, IPR013656, IPR013767	GGDEF domain-containing protein
OG0001086	WP_018170136.1	B0684_RS04105	WP_018994886.1	F468_RS0107595		hypothetical protein
OG0001089	WP_018175549.1	B0684_RS00350	WP_018993302.1	F468_RS0100305	PF13692	hypothetical protein;sugar transferase
OG0001090	WP_018175581.1	B0684_RS00135	WP_018139542.1	F468_RS0100495	PF13365	serine protease
OG0001091	WP_018175588.1	B0684_RS00060	WP_018139558.1	F468_RS0100585	GO:0006935, GO:0050568, PF03975, IPR005659	hypothetical protein
OG0001095	WP_018175630.1	B0684_RS11640	WP_018649196.1	F468_RS0102335	GO:0005524, PF00118, IPR002423	chaperonin GroEL
OG0001100	WP_018175660.1	B0684_RS12580	WP_018138132.1	F468_RS0109460		hypothetical protein
OG0001101	WP_018175662.1	B0684_RS00460	WP_038038509.1	F468_RS0109450	GO:0007165, GO:0016020, PF00015, PF13675, IPR004089, IPR029095	chemotaxis protein
OG0001105	WP_018175768.1	B0684_RS04560	WP_018137747.1	F468_RS0107140	PF01814, IPR012312	hemerythrin domain-containing protein
OG0001107	WP_018175794.1	B0684_RS10805	WP_018174630.1	F468_RS0103585	PF00753, IPR001279	MBL fold metallo-hydrolase
OG0001113	WP_018175811.1	B0684_RS10680	WP_018994748.1	F468_RS0105730	PF02578, IPR003730	multi-copper polyphenol oxidoreductase
OG0001126	WP_018175917.1	B0684_RS07690	WP_026296476.1	F468_RS0100715	PF01171, IPR011063	tRNA 2-thiocytidine(32) synthetase TtcA;potassium ABC transporter ATPase
OG0001132	WP_018175925.1	B0684_RS07820	WP_018994442.1	F468_RS0100840	GO:0003677, GO:0003887, GO:0006260, GO:0008408, GO:0009360, PF00712, PF02767, PF02768, IPR022634, IPR022635, IPR022637	DNA polymerase III subunit beta
OG0001137	WP_018175944.1	B0684_RS02795	WP_018994474.1	F468_RS0101300	GO:0008047, GO:0008233, PF01750, IPR000671	hypothetical protein
OG0001138	WP_018175945.1	B0684_RS02800	WP_018994475.1	F468_RS0101305	GO:0016151, PF00374, IPR001501	Ni/Fe hydrogenase subunit alpha
OG0001139	WP_018175948.1	B0684_RS02815	WP_018994478.1	F468_RS0101320	PF00027, IPR000595	cyclic nucleotide-binding domain-containing protein
OG0001140	WP_018175949.1	B0684_RS02820	WP_018994479.1	F468_RS0101325	PF17179, IPR017896	sulfite reductase subunit A
OG0001143	WP_018175953.1	B0684_RS02845	WP_018139826.1	F468_RS0101340	PF00316, IPR033391	class 1 fructose-bisphosphatase
OG0001146	WP_018175958.1	B0684_RS02895	WP_018994483.1	F468_RS0101380	PF01019	gamma-glutamyltransferase
OG0001152	WP_018175972.1	B0684_RS03095	WP_051080566.1	F468_RS0100935	PF00593, PF07715, IPR000531, IPR012910	TonB-dependent receptor
OG0001154	WP_018175974.1	B0684_RS03105	WP_018994454.1	F468_RS0100925	GO:0008272, GO:0015116, GO:0016021, PF00916, PF01740, IPR002645, IPR011547	SulP family inorganic anion transporter
OG0001167	WP_018176073.1	B0684_RS06920	WP_018994075.1	F468_RS0106240	GO:0009055, GO:0020037, PF00034, IPR009056	hypothetical protein;cytochrome c
OG0001176	WP_018176096.1	B0684_RS06615	WP_018649044.1	F468_RS0104030	PF00534, PF13439, IPR001296, IPR028098	hypothetical protein

Orthogroup	$AL2^T$ protein ID	$AL2^T$ locus tag	$ALJ2$ protein ID	$ALJ2$ locus tag	GO, PFAM and IPR ID's	Protein product
OG0001177	WP_018176101.1	B0684_RS06540	WP_038029928.1	F468_RS0106310	GO:0000413, GO:0003755, PF00160, IPR002130	cyclophilin;peptidylprolyl isomerase
OG0001179	WP_018176124.1	B0684_RS13135	WP_018994766.1	F468_RS0106070	GO:0006464, PF03099, IPR004143	lipoyl(octanoyl) transferase LipB
OG0001182	WP_018176170.1	B0684_RS05020	WP_018138967.1	F468_RS0110250	PF13419, IPR041492	haloacid dehalogenase type II
OG0001190	WP_018176228.1	B0684_RS08990	WP_026296486.1	F468_RS0101945	PF03781, IPR005532	Sulphatase-modifying factor protein
OG0001191	WP_018176230.1	B0684_RS08980	WP_018994531.1	F468_RS0101940	PF07963, PF16732, IPR012902, IPR031982	prepilin-type cleavage/methylation domain-containing protein;type IV pilin protein
OG0001200	WP_018176264.1	B0684_RS08680	WP_018994515.1	F468_RS0101805	GO:0016491, GO:0020037, GO:0055114, PF00042, PF00175, PF00970, IPR000971, IPR001433, IPR008333	NO-inducible flavohemoprotein
OG0001201	WP_018176265.1	B0684_RS08670	WP_038048930.1	F468_RS0101795	PF13511, IPR025392	DUF4124 domain-containing protein
OG0001203	WP_018176270.1	B0684_RS08625	WP_018995058.1	F468_RS0109835	GO:0033743, GO:0055114, PF01641, IPR002579	peptide-methionine (R)-S-oxide reductase
OG0001206	WP_018176274.1	B0684_RS10585	WP_018995055.1	F468_RS0109815	GO:0005515, PF13409, PF13410, IPR004045	glutathione S-transferase family protein
OG0001208	WP_018176278.1	B0684_RS10545	WP_018138894.1	F468_RS0109800		hypothetical protein
OG0001223	WP_018176324.1	B0684_RS13015	WP_018993270.1	F468_RS0109220	PF08279, PF13280, IPR013196, IPR026881	YafY family transcriptional regulator
OG0001230	WP_018176416.1	B0684_RS13640	WP_081601201.1	F468_RS0106580	PF00929, IPR013520	3'-5' exonuclease;DNA polymerase III subunit epsilon
OG0001231	WP_018176418.1	B0684_RS13590	WP_018138476.1	F468_RS0106570	GO:0003700, GO:0006355, PF00126, IPR000847	LysR family transcriptional regulator
OG0001234	WP_018176427.1	B0684_RS11355	WP_018995187.1	F468_RS0111070	PF08238, IPR006597	hypothetical protein;sel1 repeat family protein
OG0001236	WP_018176433.1	B0684_RS11310	WP_018137864.1	F468_RS0111155	GO:0007165, GO:0016020, GO:0016021, PF00015, PF00672, PF12729, IPR003660, IPR004089, IPR024478	methyl-accepting chemotaxis protein
OG0001237	WP_018176434.1	B0684_RS11305	WP_018137879.1	F468_RS0111080	PF02635, IPR003787	hypothetical protein
OG0001238	WP_018176435.1	B0684_RS11300	WP_018174827.1	F468_RS0111105		hypothetical protein
OG0001250	WP_018176517.1	B0684_RS09605	WP_051068167.1	F468_RS13725		hypothetical protein
OG0001251	WP_018176559.1	B0684_RS06450	WP_018994278.1	F468_RS0105450	GO:0042597, PF07813, PF13801, IPR012899, IPR025961	hypothetical protein
OG0001254	WP_018176625.1	B0684_RS00645	WP_018993580.1	F468_RS0112245	PF03625, IPR005180	DUF302 domain-containing protein
OG0001257	WP_018176642.1	B0684_RS00785	WP_018995249.1	F468_RS0111980	PF07317, IPR009926	flagellar brake protein;hypothetical protein
OG0001258	WP_018176643.1	B0684_RS00795	WP_018139150.1	F468_RS0111990	PF04316, IPR031316	flagellar biosynthesis anti-sigma factor FlgM
OG0001259	WP_018176668.1	B0684_RS01000	WP_018137705.1	F468_RS0107420	PF00581, IPR001763	sulfurtransferase
OG0001260	WP_018176695.1	B0684_RS07960	WP_018139090.1	F468_RS0112310	PF03928, IPR005624	heme-binding protein
OG0001261	WP_018176702.1	B0684_RS08010	WP_018995277.1	F468_RS0112345	GO:0003824, GO:0016491, GO:0050660, GO:0055114, PF01565, PF02913, IPR004113, IPR006094	FAD-binding oxidoreductase
OG0001264	WP_018176716.1	B0684_RS08155	WP_018995359.1	F468_RS0113185	PF00494	squalene synthase HpnC
OG0001265	WP_018176739.1	B0684_RS01235	WP_018139641.1	F468_RS0113390	PF04977, IPR007060	cell division protein FtsB
OG0001283	WP_018176828.1	B0684_RS01595	WP_018862903.1	F468_RS0108750	GO:0006935, GO:0007165, PF01584, IPR002545	chemotaxis protein CheW
OG0001284	WP_018176829.1	B0684_RS01600	WP_018174319.1	F468_RS0108755	GO:0006935, GO:0007165, PF01584, IPR002545	hypothetical protein

Orthogroup	$AL2^T$ protein ID	$AL2^T$ locus tag	$ALJ2$ protein ID	$ALJ2$ locus tag	GO, PFAM and IPR ID's	Protein product
OG0001285	WP_018176830.1	B0684_RS01620	WP_018139240.1	F468_RS0108775	GO:0000156, GO:0000160, GO:0005737, GO:0006935, GO:0008984, PF00072, PF01339, IPR000673, IPR001789	chemotaxis response regulator protein-glutamate methylesterase
OG0001286	WP_018176832.1	B0684_RS01630	WP_018139238.1	F468_RS0108785	GO:0003824, GO:0009288, GO:0050920, PF04344, IPR007439	protein phosphatase CheZ
OG0001287	WP_018176836.1	B0684_RS01665	WP_012982569.1	F468_RS0108820	GO:0006605, GO:0016020, PF01311, IPR002010	flagellar biosynthetic protein FliR
OG0001289	WP_018176866.1	B0684_RS02030	WP_018995310.1	F468_RS0112640	PF01230, IPR001310	HIT family protein
OG0001290	WP_018176934.1	B0684_RS04805	WP_018138474.1	F468_RS0106560	PF03625, IPR005180	DUF302 domain-containing protein
OG0001297	WP_018177035.1	B0684_RS08445	WP_018139076.1	F468_RS0112385	GO:0015075, GO:0016021, GO:0034220, PF04066, IPR007208	multiple resistance and pH regulation protein F
OG0001301	WP_018649563.1	B0684_RS13150	WP_018174993.1	F468_RS0100230	PF02518, PF13185, IPR003018, IPR003594	PEP-CTERM system histidine kinase PrsK
OG0001302	WP_018649564.1	B0684_RS13165	WP_018993292.1	F468_RS0100215	PF02698, IPR003848	YdcF family protein
OG0001307	WP_018649575.1	B0684_RS07385	WP_018649114.1	F468_RS0100100	GO:0009143, GO:0047429, PF01725, IPR002637	non-canonical purine NTP pyrophosphatase, RdgB/HAM1 family
OG0001322	WP_018649591.1	B0684_RS07555	WP_012981440.1	F468_RS0100985	PF13609, IPR033900	hypothetical protein;porin
OG0001327	WP_018649597.1	B0684_RS02750	WP_018175108.1	F468_RS0101260	PF01455, IPR001109	HypC/HybG/HupF family hydrogenase formation chaperone
OG0001329	WP_018649602.1	B0684_RS02810	WP_018994477.1	F468_RS0101315	GO:0016491, GO:0055114, PF00175, PF10418, IPR001433, IPR019480	hypothetical protein
OG0001332	WP_018649608.1	B0684_RS02965	WP_012982711.1	F468_RS0108165	PF13437, PF13533, IPR039562	HlyD family type I secretion periplasmic adaptor subunit
OG0001334	WP_018649610.1	B0684_RS03080	WP_018649151.1	F468_RS0101125	GO:0003723, GO:0006396, GO:0008173, PF00588, IPR001537	tRNA (cytidine(34)-2'-O)-methyltransferase
OG0001337	WP_018649613.1	B0684_RS03150	WP_018994498.1	F468_RS0101625	PF01078, PF13335, PF13541, IPR000523, IPR025158	ATP-dependent protease
OG0001344	WP_018649627.1	B0684_RS12785	WP_012983664.1	F468_RS0109910		hypothetical protein
OG0001354	WP_018649646.1	B0684_RS11750	WP_018137961.1	F468_RS0102520	PF06844, IPR023163	DUF1244 domain-containing protein
OG0001355	WP_018649647.1	B0684_RS11755	WP_018994579.1	F468_RS0102525	GO:0008360, GO:0016021, PF04093, IPR007227	rod shape-determining protein MreD
OG0001356	WP_018649649.1	B0684_RS11800	WP_026296410.1	F468_RS0102570		hypothetical protein
OG0001357	WP_018649651.1	B0684_RS11810	WP_018994583.1	F468_RS0102575	PF13847, PF17827, IPR025714, IPR040758	peptide chain release factor N(5)-glutamine methyltransferase
OG0001367	WP_018649666.1	B0684_RS11535	WP_018137839.1	F468_RS0102860	PF12710	HAD-IB family hydrolase
OG0001375	WP_018649676.1	B0684_RS03660	WP_018994603.1	F468_RS0103080	GO:0005524, GO:0006457, GO:0051082, PF00183, PF13589, IPR001404	molecular chaperone HtpG
OG0001376	WP_018649678.1	B0684_RS03690	WP_018994605.1	F468_RS0103125	PF10636, IPR019600	hemin uptake protein HemP
OG0001378	WP_018649681.1	B0684_RS03725	WP_018137669.1	F468_RS0103160		hypothetical protein
OG0001382	WP_018649685.1	B0684_RS03780	WP_018137744.1	F468_RS0103215		hypothetical protein
OG0001383	WP_018649690.1	B0684_RS03875	WP_018137726.1	F468_RS0103305		hypothetical protein
OG0001384	WP_018649691.1	B0684_RS03890	WP_018137725.1	F468_RS0103315	PF02698, IPR003848	YdcF family protein
OG0001391	WP_018649702.1	B0684_RS13385	WP_018138593.1	F468_RS0103375	GO:0008033, PF01715, IPR018022	tRNA (adenosine(37)-N6)-dimethylallyltransferase MiaA

Orthogroup	$AL2^T$ protein ID	$AL2^T$ locus tag	$ALJ2$ protein ID	$ALJ2$ locus tag	GO, PFAM and IPR ID's	Protein product
OG0001392	WP_018649704.1	B0684_RS13400	WP_018993763.1	F468_RS0103390	PF01464, PF01476, IPR008258, IPR018392	LysM peptidoglycan-binding domain-containing protein;lytic transglycosylase
OG0001397	WP_018649711.1	B0684_RS10675	WP_018138614.1	F468_RS0105725	GO:0001522, GO:0003723, GO:0009451, GO:0009982, PF00849, PF01479, IPR002942, IPR006145	23S rRNA pseudouridine(1911/1915/1917) synthase RluD
OG0001413	WP_018649734.1	B0684_RS11890	WP_018994772.1	F468_RS0106225	GO:0003723, GO:0006396, GO:0008168, GO:0008173, PF00588, PF08032, IPR001537, IPR013123	23S rRNA (guanosine(2251)-2'-O)-methyltransferase RlmB
OG0001416	WP_018649740.1	B0684_RS06535	WP_018175457.1	F468_RS0106305	GO:0016787, PF00149, PF12850, IPR004843, IPR024654	UDP-2,3-diacylglucosamine diphosphatase
OG0001420	WP_018649746.1	B0684_RS06785	WP_018138330.1	F468_RS0104195		hypothetical protein
OG0001432	WP_018649769.1	B0684_RS07255	WP_018993738.1	F468_RS0103710	PF00708, IPR001792	hypothetical protein;acylphosphatase
OG0001442	WP_018649787.1	B0684_RS03550	WP_038049058.1	F468_RS0106845		hypothetical protein
OG0001444	WP_018649791.1	B0684_RS03525	WP_018994816.1	F468_RS0106860	PF02353	class I SAM-dependent methyltransferase
OG0001445	WP_018649792.1	B0684_RS03520	WP_018994817.1	F468_RS0106865	PF07103, IPR010775	DUF1365 domain-containing protein
OG0001449	WP_018649798.1	B0684_RS03485	WP_018138687.1	F468_RS0104800	GO:0008654, GO:0016020, GO:0016780, PF01066, IPR000462	hypothetical protein;CDP-alcohol phosphatidyltransferase family protein
OG0001451	WP_018649804.1	B0684_RS03425	WP_018137711.1	F468_RS0107450	PF01656, IPR002586	ParA;hypothetical protein
OG0001453	WP_018649806.1	B0684_RS03400	WP_018649026.1	F468_RS0104330	GO:0003824, PF00501, IPR000873	long-chain fatty acid–CoA ligase
OG0001475	WP_018649842.1	B0684_RS08430	WP_018995279.1	F468_RS0112370	PF00420, IPR039428	Na+/H+ antiporter subunit C
OG0001476	WP_018649843.1	B0684_RS08435	WP_018995280.1	F468_RS0112375	PF04039, PF13244, IPR007182, IPR025383	DUF4040 domain-containing protein
OG0001478	WP_018649845.1	B0684_RS08450	WP_026296539.1	F468_RS0112390	GO:0006812, GO:0008324, GO:0016021, PF01899, IPR002758	hypothetical protein;cation transporter
OG0001484	WP_018649854.1	B0684_RS08505	WP_018994835.1	F468_RS0107060	GO:0016020, PF01758, IPR002657	hypothetical protein
OG0001489	WP_018649861.1	B0684_RS08570	WP_018995227.1	F468_RS0111555	GO:0005524, GO:0016021, GO:0016887, GO:0042626, GO:0055085, PF00005, PF00664, IPR003439, IPR011527	thiol reductant ABC exporter subunit CydC
OG0001490	WP_018649863.1	B0684_RS08580	WP_018138860.1	F468_RS0111565	GO:0016491, GO:0055114, PF07992, PF10518, IPR019546, IPR023753	hypothetical protein;NAD(P)/FAD-dependent oxidoreductase
OG0001491	WP_018649864.1	B0684_RS08585	WP_018138859.1	F468_RS0111570	GO:0009055, GO:0019646, GO:0070069, PF01654, IPR002585	cytochrome ubiquinol oxidase subunit I
OG0001492	WP_018649866.1	B0684_RS08600	WP_018138856.1	F468_RS0111585		hypothetical protein
OG0001493	WP_018649867.1	B0684_RS08605	WP_018138855.1	F468_RS0111590	GO:0016209, GO:0016491, GO:0051920, GO:0055114, PF00578, PF10417, IPR000866, IPR019479	peroxiredoxin
OG0001499	WP_018649886.1	B0684_RS04125	WP_018994898.1	F468_RS0107685		hypothetical protein
OG0001500	WP_018649889.1	B0684_RS04135	WP_018994997.1	F468_RS0109010	GO:0003824, GO:0050662, PF01370, IPR001509	complex I NDUFA9 subunit family protein
OG0001502	WP_018649898.1	B0684_RS04185	WP_018994881.1	F468_RS0107565		hypothetical protein
OG0001506	WP_018649911.1	B0684_RS04240	WP_018139295.1	F468_RS0107795		hypothetical protein
OG0001508	WP_018649913.1	B0684_RS04250	WP_018994907.1	F468_RS0107810	PF09864, IPR018660	hypothetical protein
OG0001510	WP_018649920.1	B0684_RS04290	WP_018174205.1	F468_RS0107865	GO:0006629, PF01734, IPR002641	hypothetical protein
OG0001512	WP_018649923.1	B0684_RS04315	WP_018994910.1	F468_RS0107880	GO:0055114, PF00107, PF08240, IPR013149, IPR013154	oxidoreductase

Orthogroup	$AL2^T$ protein ID	$AL2^T$ locus tag	$ALJ2$ protein ID	$ALJ2$ locus tag	GO, PFAM and IPR ID's	Protein product
OG0001516	WP_018649938.1	B0684_RS04435	WP_018139328.1	F468_RS0107965	GO:0008270, PF01258, IPR000962	DksA/TraR family C4-type zinc finger protein
OG0001519	WP_018649943.1	B0684_RS04470	WP_018139331.1	F468_RS0107980	GO:0002161, PF04073, IPR007214	hypothetical protein
OG0001526	WP_018649954.1	B0684_RS04550	WP_018174114.1	F468_RS0107125	GO:0055085, PF07690, IPR011701	MFS transporter
OG0001527	WP_018649955.1	B0684_RS04555	WP_018994844.1	F468_RS0107135	PF01814, PF10006, IPR012312, IPR018720	DUF2249 domain-containing protein
OG0001529	WP_018649957.1	B0684_RS04580	WP_018993989.1	F468_RS0107160	PF00581, IPR001763	rhodanese-like domain-containing protein
OG0001534	WP_018649965.1	B0684_RS04635	WP_018995209.1	F468_RS0111335	PF13649, IPR041698	class I SAM-dependent methyltransferase
OG0001535	WP_018649966.1	B0684_RS04640	WP_018995151.1	F468_RS0110835	GO:0047429, PF02545, IPR003697	septum formation inhibitor Maf
OG0001537	WP_018649969.1	B0684_RS04680	WP_018138530.1	F468_RS0110795	PF03167, IPR005122	SPO1 DNA polymerase
OG0001539	WP_018649988.1	B0684_RS00480	WP_018995203.1	F468_RS0111290	GO:0003677, GO:0004803, GO:0006313, PF01797, IPR002686	hypothetical protein;transposase
OG0001541	WP_018649990.1	B0684_RS00515	WP_018995260.1	F468_RS0112120	GO:0016020, GO:0022857, GO:0055085, PF00939, IPR001898	anion transporter
OG0001542	WP_018649991.1	B0684_RS00520	WP_018139124.1	F468_RS0112125	GO:0007165, GO:0016020, GO:0016021, PF00015, PF00672, PF14827, IPR003660, IPR004089, IPR029150	methyl-accepting chemotaxis protein
OG0001546	WP_018649995.1	B0684_RS00550	WP_018995263.1	F468_RS0112155	GO:0016020, GO:0055085, PF00528, IPR000515	phosphate ABC transporter, permease protein PstA
OG0001549	WP_018649999.1	B0684_RS00575	WP_038049255.1	F468_RS0112180	PF12706, IPR001279	3',5'-cyclic-nucleotide phosphodiesterase
OG0001551	WP_018650002.1	B0684_RS00640	WP_018139104.1	F468_RS0112240	GO:0016627, GO:0050660, GO:0055114, PF02771, IPR013786	acyl-CoA dehydrogenase
OG0001552	WP_018650003.1	B0684_RS00650	WP_018995271.1	F468_RS0112250	GO:0005506, GO:0016491, GO:0055114, PF00301, PF07992, PF18113, IPR023753, IPR024935, IPR041364	hypothetical protein
OG0001555	WP_018650008.1	B0684_RS00705	WP_018139164.1	F468_RS0111905		hypothetical protein
OG0001556	WP_018650009.1	B0684_RS00710	WP_018174463.1	F468_RS0111910	PF05114, IPR007801	DUF692 domain-containing protein
OG0001557	WP_018650010.1	B0684_RS00715	WP_018174464.1	F468_RS0111915	PF09836, IPR018640	DUF2063 domain-containing protein
OG0001559	WP_018650013.1	B0684_RS00830	WP_026278842.1	F468_RS0112020	PF03963, PF13860, PF13861, IPR005648, IPR025963, IPR025965	hypothetical protein;flagellar hook capping protein
OG0001560	WP_018650014.1	B0684_RS00835	WP_018139144.1	F468_RS0112025	GO:0071973, PF00460, PF06429, PF07559, IPR001444, IPR010930, IPR011491	flagellar hook protein FlgE
OG0001561	WP_018650016.1	B0684_RS00860	WP_018993597.1	F468_RS0112050	GO:0004040, PF01832, PF10135, IPR002901, IPR019301	flagellar assembly peptidoglycan hydrolase FlgJ
OG0001562	WP_018650017.1	B0684_RS00865	WP_018995251.1	F468_RS0112055	GO:0071973, PF00460, PF06429, IPR001444, IPR010930	flagellar hook-associated protein FlgK
OG0001563	WP_018650018.1	B0684_RS00870	WP_018995252.1	F468_RS0112060	GO:0005198, GO:0071973, PF00669, PF00700, IPR001029	flagellar hook-associated protein 3
OG0001564	WP_018650019.1	B0684_RS00875	WP_018139136.1	F468_RS0112065		hypothetical protein
OG0001565	WP_018650020.1	B0684_RS00880	WP_018993594.1	F468_RS0112070	PF04402, IPR007497	SIMPL domain-containing protein
OG0001568	WP_018650023.1	B0684_RS00895	WP_018649361.1	F468_RS0112085	PF10017, IPR019257	L-histidine N(alpha)-methyltransferase
OG0001575	WP_018650034.1	B0684_RS01005	WP_018139302.1	F468_RS0107830	GO:0003677, GO:0006355, PF13545, IPR012318	Crp/Fnr family transcriptional regulator
OG0001576	WP_018650051.1	B0684_RS07925	WP_018994870.1	F468_RS0107500	GO:0008757, PF05724, IPR008854	methyltransferase domain-containing protein
OG0001578	WP_018650055.1	B0684_RS07955	WP_018138037.1	F468_RS0105495	GO:0016021, PF01914, IPR002771	MarC family protein

Orthogroup	$AL2^T$ protein ID	$AL2^T$ locus tag	$ALJ2$ protein ID	$ALJ2$ locus tag	GO, PFAM and IPR ID's	Protein product
OG0001580	WP_018650058.1	B0684_RS07975	WP_018649454.1	F468_RS0107720	GO:0016787, PF01738, IPR002925	DeoR family transcriptional regulator;alpha/beta hydrolase
OG0001581	WP_018650059.1	B0684_RS07980	WP_018994901.1	F468_RS0107725	GO:0009116, PF00156, IPR000836	hypothetical protein
OG0001590	WP_018650073.1	B0684_RS01075	WP_018995366.1	F468_RS0113230		hypothetical protein
OG0001591	WP_018650074.1	B0684_RS01080	WP_018139612.1	F468_RS0113235	PF00691, IPR006665	membrane protein
OG0001592	WP_018650075.1	B0684_RS01085	WP_018995367.1	F468_RS0113240	GO:0016779, PF01842, PF01909, PF01966, PF08335, IPR002912, IPR002934, IPR006674, IPR013546	[protein-PII] uridylyltransferase
OG0001597	WP_018650082.1	B0684_RS01175	WP_026296545.1	F468_RS0113330	PF01351, IPR024567	ribonuclease HII
OG0001612	WP_018650098.1	B0684_RS01325	WP_018648908.1	F468_RS0113480	GO:0016020, PF04893, IPR006977	YIP1 family protein;DUF1282 domain-containing protein
OG0001613	WP_018650102.1	B0684_RS01345	WP_018995287.1	F468_RS0112445	GO:0007155, GO:0009288, GO:0009424, PF02465, PF07195, PF07196, IPR003481, IPR010809, IPR010810	hypothetical protein
OG0001614	WP_018650103.1	B0684_RS01350	WP_018173933.1	F468_RS0112450	GO:0044780, PF02561, IPR003713	flagella export chaperone FliS
OG0001615	WP_018650104.1	B0684_RS01355	WP_018995288.1	F468_RS0112455	PF05400, IPR008622	flagellar protein FliT
OG0001619	WP_018650118.1	B0684_RS01445	WP_018139675.1	F468_RS0112500	PF09350, IPR018961	DUF1992 domain-containing protein
OG0001626	WP_018650134.1	B0684_RS10230	WP_081616970.1	F468_RS0108365	GO:0008033, GO:0017150, GO:0050660, GO:0055114, PF01207, IPR001269	tRNA dihydrouridine(20/20a) synthase DusA
OG0001628	WP_018650136.1	B0684_RS10220	WP_018139407.1	F468_RS0108375	GO:0004018, GO:0006188, PF00206, PF08328, IPR013539, IPR022761	adenylosuccinate lyase
OG0001643	WP_018650155.1	B0684_RS01625	WP_018994973.1	F468_RS0108780	GO:0000155, GO:0000160, GO:0004673, GO:0005737, GO:0006935, GO:0007165, PF01584, PF01627, PF02518, PF02895, IPR002545, IPR003594, IPR004105, IPR008207	chemotaxis protein CheA
OG0001644	WP_018650157.1	B0684_RS01655	WP_018139233.1	F468_RS0108810	GO:0009306, GO:0016020, PF00771, IPR001712	flagellar biosynthesis protein FlhA
OG0001645	WP_018650158.1	B0684_RS01660	WP_018994976.1	F468_RS0108815	GO:0009306, GO:0016020, PF01312, IPR006135	flagellar biosynthesis protein FlhB
OG0001646	WP_018650160.1	B0684_RS01695	WP_018994977.1	F468_RS0108850	GO:0006935, GO:0009425, GO:0071973, PF03748, IPR005503	hypothetical protein
OG0001647	WP_018650161.1	B0684_RS01700	WP_018994978.1	F468_RS0108855	PF02120, IPR021136	flagellar hook-length control protein FliK
OG0001648	WP_018650162.1	B0684_RS01705	WP_026296515.1	F468_RS0108860	GO:0009288, GO:0071973, PF02050, IPR012823	flagellar export protein FliJ
OG0001649	WP_018650163.1	B0684_RS01725	WP_018139221.1	F468_RS0108880	PF01514, PF08345, IPR006182, IPR013556	flagellar M-ring protein FliF
OG0001650	WP_018650164.1	B0684_RS01730	WP_018139220.1	F468_RS0108885	GO:0003774, GO:0005198, GO:0009288, GO:0071973, PF02049, IPR001624	flagellar hook-basal body complex protein FliE
OG0001653	WP_018650173.1	B0684_RS01795	WP_018994990.1	F468_RS0108975	GO:0003677, GO:0004519, GO:0006304, PF04313, PF18766, IPR007409, IPR040980	type I restriction endonuclease subunit R
OG0001655	WP_018650176.1	B0684_RS01810	WP_018994995.1	F468_RS0109000	GO:0003677, GO:0006306, GO:0008170, PF02384, PF12161, IPR003356, IPR022749	SAM-dependent DNA methyltransferase
OG0001658	WP_018650186.1	B0684_RS01885	WP_018994891.1	F468_RS0107630	GO:0003824, GO:0016746, PF00501, PF00550, PF01553, PF13193, IPR000873, IPR002123, IPR009081, IPR025110	AMP-dependent synthetase

Orthogroup	$AL2^T$ protein ID	$AL2^T$ locus tag	$ALJ2$ protein ID	$ALJ2$ locus tag	GO, PFAM and IPR ID's	Protein product
OG0001663	WP_018650198.1	B0684_RS01990	WP_018173902.1	F468_RS0112680	GO:0005975, GO:0016868, GO:0071704, PF00408, PF02878, PF02879, PF02880, IPR005843, IPR005844, IPR005845, IPR005846	phosphomannomutase/phosphoglucomutase
OG0001674	WP_018650213.1	B0684_RS02115	WP_038038771.1	F468_RS0108295	PF11845, IPR021796	DUF3365 domain-containing protein
OG0001675	WP_018650214.1	B0684_RS02120	WP_018139391.1	F468_RS0108290	PF13426, IPR000014	PAS sensor domain-containing protein
OG0001676	WP_018650215.1	B0684_RS02125	WP_081601220.1	F468_RS0108285	GO:0001522, GO:0003723, GO:0009451, GO:0009982, PF01142, IPR001656	tRNA pseudouridine(13) synthase TruD
OG0001677	WP_018650217.1	B0684_RS02140	WP_018139387.1	F468_RS0108270	PF14559	hypothetical protein
OG0001680	WP_018650222.1	B0684_RS02185	WP_018994940.1	F468_RS0108225	GO:0006400, GO:0016763, PF01702, IPR002616	tRNA-guanine(34) transglycosylase;tRNA guanosine(34) transglycosylase Tgt
OG0001681	WP_018650223.1	B0684_RS02190	WP_018993883.1	F468_RS0108220	GO:0008616, GO:0016740, GO:0016853, PF02547, IPR003699	tRNA preQ1(34) S-adenosylmethionine ribosyltransferase-isomerase QueA
OG0001692	WP_018650240.1	B0684_RS02315	WP_018139345.1	F468_RS0108050		hypothetical protein;LysM peptidoglycan-binding domain-containing protein
OG0001703	WP_018650253.1	B0684_RS02405	WP_018139178.1	F468_RS0111835	PF04298, IPR007395	peptidase
OG0001711	WP_018650272.1	B0684_RS04790	WP_018139588.1	F468_RS0113100	GO:0003677, GO:0004519, GO:0009307, PF04471, IPR007560	hypothetical protein
OG0001714	WP_018650305.1	B0684_RS06480	WP_018995128.1	F468_RS0110630	GO:0003677, GO:0004803, GO:0006313, PF01797, IPR002686	hypothetical protein
OG0001721	WP_018650315.1	B0684_RS06185	WP_018174040.1	F468_RS0110725		hypothetical protein
OG0001732	WP_018650330.1	B0684_RS06020	WP_018138737.1	F468_RS0105075	PF04244, IPR007357	hypothetical protein;cryptochrome/photolyase family protein
OG0001733	WP_018650331.1	B0684_RS06015	WP_018994709.1	F468_RS0105070	PF00875, PF03441, IPR005101, IPR006050	deoxyribodipyrimidine photo-lyase
OG0001735	WP_018650333.1	B0684_RS05965	WP_012982223.1	F468_RS0105020	PF07396, IPR010870	hypothetical protein;phosphate porin
OG0001742	WP_018650341.1	B0684_RS05695	WP_018994688.1	F468_RS0104750	GO:0003700, GO:0006355, PF00581, PF01022, IPR001763, IPR001845	hypothetical protein;ArsR family transcriptional regulator
OG0001745	WP_018650345.1	B0684_RS05640	WP_012982158.1	F468_RS0104695		hypothetical protein
OG0001753	WP_018650359.1	B0684_RS05540	WP_018995226.1	F468_RS0111550	PF13419, IPR041492	phosphatase
OG0001768	WP_018650385.1	B0684_RS12055	WP_018994792.1	F468_RS0106600	GO:0008773, PF00027, PF00571, PF03445, PF10335, IPR000595, IPR000644, IPR005105, IPR018821	CBS domain-containing protein
OG0001771	WP_018650390.1	B0684_RS11350	WP_018174828.1	F468_RS0111100	GO:0003677, GO:0032784, PF01272, IPR001437	hypothetical protein
OG0001772	WP_018650396.1	B0684_RS11165	WP_018994188.1	F468_RS0111205	GO:0003676, GO:0004519, PF01844, IPR002711	HNH endonuclease
OG0001778	WP_018650407.1	B0684_RS09310	WP_012982072.1	F468_RS0104260	PF09335, IPR032816	TVP38/TMEM64 family protein
OG0001785	WP_018650424.1	B0684_RS09485	WP_018138060.1	F468_RS0105610	PF05957, IPR010279	hypothetical protein;DUF883 domain-containing protein
OG0001791	WP_018650435.1	B0684_RS09555	WP_018138638.1	F468_RS0105860	GO:0005525, PF01018, PF01926, IPR006073, IPR006169	GTPase ObgE
OG0001796	WP_018650443.1	B0684_RS09630	WP_038038639.1	F468_RS0103500	PF13505, IPR027385	hypothetical protein
OG0001797	WP_018650444.1	B0684_RS09635	WP_018138570.1	F468_RS0103495	GO:0009055, GO:0019646, PF01355, IPR000170	hypothetical protein;high potential iron-sulfur protein

Orthogroup	*AL2*^{T} protein ID	*AL2*^{T} locus tag	*ALJ2* protein ID	*ALJ2* locus tag	GO, PFAM and IPR ID's	Protein product
OG0001798	WP_018650446.1	B0684_RS09645	WP_018174458.1	F468_RS0111880		hypothetical protein
OG0001803	WP_018650452.1	B0684_RS09690	WP_038049037.1	F468_RS0105825	GO:0004222, GO:0006508, GO:0008270, GO:0031012, PF00413, IPR001818	hypothetical protein;peptidase M10
OG0001816	WP_018650469.1	B0684_RS06235	WP_018994741.1	F468_RS0105555	GO:0006629, PF01734, IPR002641	serine protease
OG0001818	WP_018650471.1	B0684_RS06260	WP_018994740.1	F468_RS0105540	GO:0016020, GO:0030001, GO:0046873, GO:0055085, PF01544, IPR002523	magnesium and cobalt transport protein CorA
OG0001819	WP_018650485.1	B0684_RS06355	WP_018138033.1	F468_RS0105475	GO:0004602, GO:0006979, GO:0055114, PF00255, IPR000889	glutathione peroxidase
OG0001820	WP_018650491.1	B0684_RS08205	WP_038049066.1	F468_RS0105470	GO:0010181, GO:0016491, GO:0055114, PF00724, IPR001155	alkene reductase
OG0001834	WP_018650515.1	B0684_RS03930	WP_018995108.1	F468_RS0110490	GO:0004190, GO:0016020, PF01478, PF06750, IPR000045, IPR010627	prepilin peptidase
OG0001838	WP_018650520.1	B0684_RS04905	WP_018137995.1	F468_RS0110355	PF02021, IPR003509	YraN family protein
OG0001843	WP_018650527.1	B0684_RS05110	WP_018994593.1	F468_RS0102825	GO:0016705, GO:0055114, PF00296, IPR011251	LLM class flavin-dependent oxidoreductase
OG0001844	WP_018650528.1	B0684_RS05115	WP_018175368.1	F468_RS0102830	GO:0003824, GO:0050662, PF01370, IPR001509	hypothetical protein
OG0001850	WP_018650536.1	B0684_RS05220	WP_018139043.1	F468_RS0102255	PF05137, IPR007813	pilus assembly protein PilN
OG0001863	WP_018650565.1	B0684_RS08940	WP_018994520.1	F468_RS0101860	PF01564, PF17284, IPR035246	polyamine aminopropyltransferase
OG0001867	WP_018650569.1	B0684_RS08895	WP_018138246.1	F468_RS0101815	PF12224, IPR022025	hypothetical protein
OG0001876	WP_018650585.1	B0684_RS08715	WP_018138948.1	F468_RS0110150	GO:0005524, GO:0016787, PF00580, PF13361, IPR014017, IPR034739	ATP-dependent DNA helicase Rep
OG0001880	WP_018650594.1	B0684_RS10465	WP_018995046.1	F468_RS0109720	GO:0046855, PF00459, IPR000760	inositol monophosphatase family protein
OG0001886	WP_018650601.1	B0684_RS10400	WP_018994512.1	F468_RS0101785	GO:0008705, GO:0009086, GO:0031419, GO:0042558, GO:0046872, PF00809, PF02310, PF02574, PF02607, PF02965, IPR000489, IPR003726, IPR003759, IPR004223, IPR006158	methionine synthase
OG0001888	WP_018650604.1	B0684_RS13040	WP_018995029.1	F468_RS0109260	GO:0030001, GO:0046872, PF01297, IPR006127	zinc ABC transporter substrate-binding protein
OG0001892	WP_018650610.1	B0684_RS00025	WP_018994430.1	F468_RS0100620	PF13466, IPR002645	STAS domain-containing protein
OG0001893	WP_018650613.1	B0684_RS00040	WP_018139562.1	F468_RS0100605	GO:0006935, GO:0007165, PF01584, IPR002545	chemotaxis protein CheW
OG0001894	WP_018650615.1	B0684_RS00050	WP_018994427.1	F468_RS0100595	GO:0007165, GO:0016020, PF00015, PF13426, IPR000014, IPR004089	methyl-accepting chemotaxis protein;PAS domain-containing protein
OG0001895	WP_018650616.1	B0684_RS00055	WP_018139559.1	F468_RS0100590	PF01739, PF03705, IPR022641, IPR022642	protein-glutamate O-methyltransferase CheR
OG0001896	WP_018650617.1	B0684_RS00070	WP_018994425.1	F468_RS0100575	GO:0007165, GO:0016020, PF00015, IPR004089	hypothetical protein
OG0001897	WP_018650618.1	B0684_RS00075	WP_018139555.1	F468_RS0100570	PF13466, IPR002645	anti-sigma factor antagonist
OG0001898	WP_018650619.1	B0684_RS00080	WP_018994424.1	F468_RS0100565	GO:0000160, GO:0003824, PF00072, PF07228, PF13581, IPR001789, IPR001932, IPR003594	hypothetical protein
OG0001905	WP_018650627.1	B0684_RS00145	WP_018994417.1	F468_RS0100490	PF14907, IPR039498	hypothetical protein
OG0001906	WP_018650628.1	B0684_RS00150	WP_018994416.1	F468_RS0100485		HprK-related kinase A
OG0001907	WP_018650630.1	B0684_RS00165	WP_018139537.1	F468_RS0100470	GO:0008168, PF08241, IPR013216	class I SAM-dependent methyltransferase

Orthogroup	$AL2^T$ protein ID	$AL2^T$ locus tag	$ALJ2$ protein ID	$ALJ2$ locus tag	GO, PFAM and IPR ID's	Protein product
OG0001908	WP_018650632.1	B0684_RS00175	WP_018139536.1	F468_RS0100465	PF04892, PF04932, IPR006976, IPR007016	VanZ family protein
OG0001909	WP_018650634.1	B0684_RS00185	WP_018994414.1	F468_RS0100460		hypothetical protein
OG0001910	WP_018650635.1	B0684_RS00190	WP_018139533.1	F468_RS0100450	PF02350, IPR003331	UDP-N-acetylglucosamine 2-epimerase (non-hydrolyzing)
OG0001911	WP_018650639.1	B0684_RS00205	WP_018994412.1	F468_RS0100440	GO:0005975, GO:0016810, PF01522, IPR002509	hypothetical protein;polysaccharide deacetylase
OG0001912	WP_018650643.1	B0684_RS00225	WP_038048924.1	F468_RS0100385	GO:0003824, PF00278, PF02784, IPR022643, IPR022644	pyridoxal-dependent decarboxylase, exosortase A system-associated
OG0001913	WP_018650644.1	B0684_RS00230	WP_018994403.1	F468_RS0100390	GO:0003824, PF00501, PF13193, IPR000873, IPR025110	acyl-CoA ligase (AMP-forming), exosortase A system-associated
OG0001914	WP_018650645.1	B0684_RS00235	WP_018175022.1	F468_RS0100395	PF04932, IPR007016	putative O-glycosylation ligase, exosortase A system-associated
OG0001915	WP_018650646.1	B0684_RS00240	WP_018994404.1	F468_RS0100400		hypothetical protein
OG0001916	WP_018650652.1	B0684_RS00275	WP_018994400.1	F468_RS0100375	GO:0003824, PF00501, PF13193, IPR000873, IPR025110	hypothetical protein;thioester reductase
OG0001917	WP_018650655.1	B0684_RS00290	WP_018994397.1	F468_RS0100360	PF12146, IPR022742	hydrolase 1, exosortase A system-associated
OG0001918	WP_018650656.1	B0684_RS00295	WP_018994396.1	F468_RS0100355	PF12146, IPR022742	hydrolase 2, exosortase A system-associated
OG0001919	WP_018650657.1	B0684_RS00300	WP_012983883.1	F468_RS0100350	PF00550, IPR009081	acyl carrier protein
OG0001920	WP_018650659.1	B0684_RS00305	WP_018994395.1	F468_RS0100345	GO:0004066, GO:0006529, PF00733, PF13537, IPR001962, IPR017932	hypothetical protein;asparagine synthase
OG0001921	WP_018650661.1	B0684_RS00320	WP_018994394.1	F468_RS0100340	PF00501, IPR000873	phenylacetate–CoA ligase family protein
OG0001922	WP_018650664.1	B0684_RS00335	WP_018994391.1	F468_RS0100320	GO:0004066, GO:0006529, PF00733, PF13537, IPR001962, IPR017932	amidotransferase 1, exosortase A system-associated
OG0001923	WP_018650666.1	B0684_RS00345	WP_018139505.1	F468_RS0100310	PF09721, PF11984, IPR014263, IPR019127	exosortase A
OG0001924	WP_018650668.1	B0684_RS00360	WP_018994389.1	F468_RS0100300	PF13480, IPR038740	GNAT family N-acetyltransferase
OG0001925	WP_018650669.1	B0684_RS00365	WP_018175003.1	F468_RS0100295	GO:0005975, GO:0016810, PF01522, PF11959, IPR002509, IPR022560	DUF3473 domain-containing protein
OG0001926	WP_018650670.1	B0684_RS00380	WP_018139499.1	F468_RS0100280	PF13614, IPR025669	hypothetical protein;protein tyrosine kinase
OG0001927	WP_018650671.1	B0684_RS00385	WP_018994387.1	F468_RS0100275	GO:0009103, GO:0016020, PF02706, IPR003856	hypothetical protein;LPS biosynthesis protein
OG0001928	WP_018650672.1	B0684_RS00390	WP_018139497.1	F468_RS0100270	PF07238, IPR009875	PilZ domain-containing protein
OG0001930	WP_018650674.1	B0684_RS00400	WP_018994385.1	F468_RS0100260	PF13444	PEP-CTERM/exosortase system-associated acyltransferase
OG0001931	WP_018650675.1	B0684_RS00405	WP_018174998.1	F468_RS0100255	GO:0008641, PF00899, IPR000594	hypothetical protein
OG0001936	WP_018948416.1	B0684_RS07380	WP_012983836.1	F468_RS0100105	PF01138, PF03725, IPR001247, IPR015847	ribonuclease PH
OG0001937	WP_019563614.1	B0684_RS12495	WP_018138197.1	F468_RS0105170	GO:0003735, GO:0005840, GO:0006412, PF00253, IPR001209	30S ribosomal protein S14
OG0001941	WP_019571183.1	B0684_RS07075	WP_018138642.1	F468_RS0105900	GO:0003684, GO:0003906, GO:0006284, GO:0006289, GO:0008270, GO:0016799, PF01149, PF06827, PF06831, IPR010663, IPR012319, IPR015886	bifunctional DNA-formamidopyrimidine glycosylase/DNA-(apurinic or apyrimidinic site) lyase
OG0001943	WP_019572082.1	B0684_RS10355	WP_018139433.1	F468_RS0109340		hypothetical protein
OG0001948	WP_026182005.1	B0684_RS10740	WP_017925754.1	F468_RS0105785	GO:0003700, GO:0006355, PF01022, IPR001845	ArsR family transcriptional regulator

Orthogroup	$AL2^T$ protein ID	$AL2^T$ locus tag	$ALJ2$ protein ID	$ALJ2$ locus tag	GO, PFAM and IPR ID's	Protein product
OG0001949	WP_026182008.1	B0684_RS04565	WP_018137748.1	F468_RS0107145		hypothetical protein
OG0001965	WP_026304771.1	B0684_RS04990	WP_018995087.1	F468_RS0110275	PF13561	3-oxoacyl-ACP reductase;SDR family oxidoreductase
OG0001976	WP_026305896.1	B0684_RS12245	WP_018138299.1	F468_RS0104380	GO:0003755, PF00254, PF00581, IPR001179, IPR001763	peptidylprolyl isomerase
OG0001978	WP_026305949.1	B0684_RS05875	WP_018994698.1	F468_RS0104930	GO:0009058, PF02514, IPR003672	cobalamin biosynthesis protein CobN
OG0001979	WP_026305950.1	B0684_RS05945	WP_018138726.1	F468_RS0105000	GO:0008168, PF00590, PF01135, IPR000878	bifunctional cobalt-precorrin-7 (C(5))-methyltransferase/cobalt-precorrin-6B (C(15))-methyltransferase
OG0001981	WP_026305973.1	B0684_RS01510	WP_018139686.1	F468_RS0112560	PF04982, IPR007065	HPP family protein
OG0001983	WP_026312994.1	B0684_RS07805	WP_018994441.1	F468_RS0100825	GO:0000049, GO:0004526, GO:0008033, PF00825, IPR000100	ribonuclease P protein component
OG0001984	WP_026312995.1	B0684_RS07825	WP_081616948.1	F468_RS0100845	PF13476, IPR038729	DNA replication and repair protein RecF
OG0001985	WP_026312998.1	B0684_RS03160	WP_018138211.1	F468_RS0101635	PF13561	KR domain-containing protein
OG0001988	WP_026313012.1	B0684_RS10870	WP_018174636.1	F468_RS0103650	GO:0004751, GO:0009052, PF06026, IPR004788	ribose-5-phosphate isomerase RpiA
OG0001998	WP_026313029.1	B0684_RS08515	WP_038049217.1	F468_RS0110975	GO:0015562, GO:0055085, PF02321, IPR003423	TolC family protein;transporter
OG0002000	WP_026313034.1	B0684_RS04385	WP_018139321.1	F468_RS0107930	PF00011, IPR002068	Hsp20/alpha crystallin family protein
OG0002008	WP_026313055.1	B0684_RS01680	WP_018139230.1	F468_RS0108835	GO:0016021, GO:0044781, PF04347, IPR022781	flagellar biosynthetic protein FliO
OG0002016	WP_026313083.1	B0684_RS11120	WP_018137847.1	F468_RS0111240	PF04168, IPR007296	alpha-E domain-containing protein
OG0002020	WP_026313095.1	B0684_RS05225	WP_018139042.1	F468_RS0102250	PF11104, IPR005883	pilus assembly protein PilM
OG0002021	WP_026313097.1	B0684_RS09255	WP_018994543.1	F468_RS0102125		hypothetical protein
OG0002027	WP_038037866.1	B0684_RS09140	WP_018649190.1	F468_RS0102095	PF01262, PF05222, IPR007698, IPR007886	NAD(P) transhydrogenase subunit alpha
OG0002038	WP_038096132.1	B0684_RS02575	WP_026296495.1	F468_RS0104855	PF00248, IPR023210	aldo/keto reductase
OG0002040	WP_038096420.1	B0684_RS08890	WP_038048865.1	F468_RS0101810	GO:0016787, PF07722, IPR011697	gamma-glutamyl-gamma-aminobutyrate hydrolase family protein
OG0002046	WP_051074783.1	B0684_RS13630	WP_018994801.1	F468_RS0106700		hypothetical protein
OG0002049	WP_051080701.1	B0684_RS00375	WP_018175001.1	F468_RS0100285		TIGR03016 family PEP-CTERM system-associated outer membrane protein
OG0002050	WP_051080705.1	B0684_RS05950	WP_018138727.1	F468_RS0105005	GO:0009236, PF01890, IPR002750	cobalamin biosynthesis protein;hypothetical protein
OG0002052	WP_077276934.1	B0684_RS00855	WP_018174473.1	F468_RS0112045	GO:0005198, GO:0009428, GO:0030288, GO:0071973, PF02119, IPR001782	flagellar basal body P-ring protein FlgI
OG0002054	WP_077276936.1	B0684_RS01340	WP_081616996.1	F468_RS14360	PF03646, IPR005186	flagellar biosynthesis protein FlaG;hypothetical protein
OG0002056	WP_077276942.1	B0684_RS01650	WP_081616971.1	F468_RS0108805	GO:0005525, GO:0006614, PF00448, IPR000897	flagellar biosynthesis protein FlhF
OG0002064	WP_077276953.1	B0684_RS01805	WP_018994994.1	F468_RS0108995	GO:0003677, GO:0006304, PF01420, IPR000055	restriction endonuclease subunit S
OG0002065	WP_077276954.1	B0684_RS02355	WP_018994920.1	F468_RS0108010	GO:0016021, PF03073, IPR004307	tryptophan-rich sensory protein
OG0002069	WP_077276963.1	B0684_RS03515	WP_018994818.1	F468_RS0106870	GO:0016491, GO:0055114, PF01593, IPR002937	FAD-dependent oxidoreductase;NAD/FAD-binding protein
OG0002070	WP_077276964.1	B0684_RS03435	WP_081601188.1	F468_RS14170		hypothetical protein

Orthogroup	$AL2^T$ protein ID	$AL2^T$ locus tag	$ALJ2$ protein ID	$ALJ2$ locus tag	GO, PFAM and IPR ID's	Protein product
OG0002079	WP_077276983.1	B0684_RS05105	WP_018994592.1	F468_RS0102820		hypothetical protein
OG0002082	WP_077276988.1	B0684_RS05530	WP_018995225.1	F468_RS0111540	GO:0004812, GO:0005524, GO:0043039, PF00749, IPR020058	tRNA glutamyl-Q(34) synthetase GluQRS
OG0002083	WP_077276994.1	B0684_RS06455	WP_018138533.1	F468_RS0110770	GO:0015128, GO:0016020, GO:0035429, PF02447, IPR003474	GntP family permease
OG0002093	WP_077277009.1	B0684_RS08210	WP_018995146.1	F468_RS0110765	GO:0008887, GO:0031388, PF02595, IPR004381	glycerate kinase
OG0002094	WP_077277011.1	B0684_RS08575	WP_018995228.1	F468_RS0111560	GO:0005524, GO:0016021, GO:0016887, GO:0042626, GO:0055085, PF00005, PF00664, IPR003439, IPR011527	thiol reductant ABC exporter subunit CydD
OG0002096	WP_077277013.1	B0684_RS08970	WP_018994529.1	F468_RS0101930	PF07963, IPR012902	prepilin-type cleavage/methylation domain-containing protein;hypothetical protein
OG0002097	WP_077277014.1	B0684_RS09075	WP_018139003.1	F468_RS0102030	GO:0035438, PF07238, IPR009875	PilZ domain-containing protein
OG0002100	WP_077277023.1	B0684_RS09940	WP_018994969.1	F468_RS0108660	PF00753, PF03772, PF13567, IPR001279, IPR004477, IPR025405	DNA internalization-related competence protein ComEC/Rec2
OG0002103	WP_077277032.1	B0684_RS11615	WP_018137622.1	F468_RS0102655	PF00691, IPR006665	OmpA family protein
OG0002107	WP_077277037.1	B0684_RS11835	WP_018994775.1	F468_RS0106300	PF04305, IPR007402	DUF455 domain-containing protein
OG0002111	WP_077277042.1	B0684_RS12890	WP_038048878.1	F468_RS0102315	GO:0015562, GO:0055085, PF02321, IPR003423	TolC family protein
OG0002112	WP_077277050.1	B0684_RS13140	WP_018994384.1	F468_RS0100240	PF13174, PF13432, PF14559, IPR019734	PEP-CTERM system TPR-repeat protein PrsT
