# Supplementary material for: Molecular and Physiological Adaptations to Low Temperature in Thioalkalivibrio Strains Isolated from Soda Lakes with Different Temperature Regimes
Source: mSystems. 2021 Apr 27;6(2):e01202-20. doi: 10.1128/mSystems.01202-20 (PMC8092127; doi:10.1128/mSystems.01202-20)
Supplement: TABLE S9 [file msystems.01202-20-st009.pdf]

Annotation	*Tv. versutus* AL2T			*Tv. nitratis* ALJ2		
	Locus-tag	*b*-value	P_{adj}-value	Locus-tag	*b*-value	P_{adj}-value
**(1) Membrane lipid composition**						
**Fatty acid synthesis:**						
3-hydroxyacyl-(acyl-carrier-protein) dehydratase (*fab* A)	B0684_RS05635	1.24	5.18E-21	F468_RS0104690	1.22	4.27E-38
Acyl-carrier-protein S-malonyltransferase (*fab* D)	B0684_RS10155	0.39	9.39E-04	F468_RS0108440	1.05	6.29E-12
Cyclopropane-fatty-acyl-phospholipid synthase	B0684_RS05855	-0.21	1.25E-02	F468_RS0104910	-1.06	1.65E-31
Cyclopropane-fatty-acyl-phospholipid synthase	B0684_RS03525	-0.28	7.52E-03	F468_RS0106860	-1.33	1.81E-09
Cyclopropane-fatty-acyl-phospholipid synthase	B0684_RS08230	-0.54	5.72E-09	F468_RS0106715	0.36	1.50E-04
Cyclopropane-fatty-acyl-phospholipid synthase	B0684_RS01870	-0.43	3.19E-05			
**Squalene synthesis:**						
Squalene-associated FAD-dependent desaturase (*hpn* E)	B0684_RS08130	0.75	1.94E-04	F468_RS0113170	1.28	1.60E-18
Squalene synthase *hpn* D (Farnesyl-diphosphate farnesyltransferase)	B0684_RS08135	0.62	2.82E-10	F468_RS0113175	1.07	4.60E-11
**Peptidoglycan synthesis:**						
Polysaccharide pyruvyl transferase family protein (*wca* K)	B0684_RS01385	1.20	3.92E-13			
Peptidoglycan-binding domain-containing protein (*lys* M)	B0684_RS13400	1.04	5.55E-18	F468_RS0103390	0.62	1.89E-08
Undecaprenyl pyrophosphate synthetase (EC 2.5.1.31)	B0684_RS01115	0.47	8.07E-09	F468_RS0113270	1.04	1.22E-21
Hypothetical protein	B0684_RS00185	-1.29	9.99E-18	F468_RS0100460	-0.96	2.01E-17
Polysaccharide deacetylase	B0684_RS00195	-0.87	5.88E-06	F468_RS0100445	-1.14	6.06E-15
FkbM family methyltransferase	B0684_RS00200	-0.87	1.97E-07			
Hypothetical protein (Polysaccharide deacetylase)	B0684_RS00205	-1.08	1.86E-08	F468_RS0100440	-1.20	1.49E-11
GNAT family N-acetyltransferase	B0684_RS00210	-1.02	4.61E-13			
GNAT family N-acetyltransferase	B0684_RS00215	-1.31	9.62E-16			
GNAT family N-acetyltransferase	B0684_RS00220	-1.02	2.94E-10	F468_RS0100435	-1.00	1.89E-07
Pyridoxal-dependent decarboxylase, exosortase A system-associated	B0684_RS00225	-1.03	3.15E-15	F468_RS0100385	-1.31	2.07E-29
Acyl-CoA ligase (AMP-forming), exosortase A system-associated	B0684_RS00230	-0.95	5.48E-16	F468_RS0100390	-1.21	1.30E-25

**Lipoprotein synthesis:**						
Hypothetical protein (Lipoprotein-attachment site-containing protein)	B0684_RS07645	1.05	6.46E-03	F468_RS14005	0.97	6.46E-04
Prokaryotic membrane lipoprotein lipid attachment site profile	B0684_RS06685	-1.95	2.64E-12	F468_RS0104095	-2.11	5.06E-07
Hypothetical protein (Prokaryotic membrane lipoprotein lipid attachment site profile)	B0684_RS03705	-1.25	1.46E-08			
Apolipoprotein N-acyltransferase				F468_RS0102155	1.01	7.14E-17
Prolipoprotein diacylglyceryl transferase	B0684_RS05355	0.71	7.58E-01	F468_RS0106395	1.04	6.89E-26
Hypothetical protein (Prokaryotic membrane lipoprotein lipid attachment site profile)	B0684_RS04345	-1.27	7.68E-06	F468_RS0106380	-1.63	9.56E-06
**Lipopolysaccharide synthesis:**						
UDP-2,3-diacylglucosamine diphosphatase	B0684_RS06535	-0.04	7.44E-01	F468_RS0106305	1.05	4.12E-16
Hypothetical protein (3-deoxy-D-manno-octulosonic-acid transferase)	B0684_RS03725	-0.20	1.88E-01	F468_RS0103160	1.03	2.16E-19
UDP-N-acetylglucosamine 2-epimerase (non-hydrolyzing)	B0684_RS00190	-1.16	3.68E-12	F468_RS0100450	-1.45	6.21E-24
**PEP-CTERM system:**						
Hypothetical protein (Polysacchartide chain length determination protein)	B0684_RS00385	-1.34	5.06E-10	F468_RS0100275	-1.80	3.21E-50
Exopolysaccharide/PEP-CTERM locus tyrosine autokinase	B0684_RS00380	-1.35	5.59E-09	F468_RS0100280	-1.35	4.30E-14
TIGR03016 family PEP-CTERM system-associated outer membrane protein	B0684_RS00375	-1.58	1.93E-11	F468_RS0100285	-1.48	4.39E-48
Hypothetical protein (Putative secretion ATPase)	B0684_RS00370	-1.67	9.19E-10	F468_RS0100290	-1.62	1.09E-34
DUF3473 domain-containing protein (Polysaccharide deacetylase family protein)	B0684_RS00365	-1.52	6.24E-12	F468_RS0100295	-1.58	4.35E-35
GNAT family N-acetyltransferase	B0684_RS00360	-1.20	6.42E-08	F468_RS0100300	-1.03	8.48E-18
GxxExxY protein	B0684_RS00355	-1.07	8.50E-07			
Hypothetical protein (Sugar transferase, PEP-CTERM/EpsH1 system associated)	B0684_RS00350	-1.14	2.08E-09	F468_RS0100305	-1.30	4.58E-25
Exosortase A	B0684_RS00345	-1.05	4.99E-10	F468_RS0100310	-0.93	4.32E-13

Sugar transferase, PEP-CTERM/EpsH1 system associated	B0684_RS00340	-0.98	2.41E-08	F468_RS0100315	-1.15	3.50E-11
PEP-CTERM system TPR-repeat protein PrsT, sugar transferase	B0684_RS13140	-1.18	7.48E-16	F468_RS0100240	-0.96	2.94E-24
Two-component system, PEP-CTERM-box response regulator transcription factor	B0684_RS13145	-1.35	1.86E-15	F468_RS0100235	-1.74	6.51E-34
PEP-CTERM system histidine kinase (*prs* K)	B0684_RS13150	-1.57	2.53E-24	F468_RS0100230	-1.49	4.92E-27
Hypothetical protein (sugar transferase)	B0684_RS13155	-1.67	2.88E-16	F468_RS0100225	-1.79	3.62E-22
Sugar ABC transporter substrate-binding protein (Polysaccharide export outer membrane protein)	B0684_RS13160	-1.65	5.75E-13	F468_RS0100220	-2.14	3.27E-35
**(2) Compatible solutes and antifreeze proteins**						
**Glycine betaine transporters:**						
Betaine/carnitine/choline transporter, BCCT	B0684_RS04420	-1.48	3.76E-09	F468_RS0100980	-1.64	9.62E-25
Betaine/carnitine/choline transporter, BCCT	B0684_RS09355	-0.58	3.13E-11	F468_RS0106375	-0.84	1.41E-09
Betaine/carnitine/choline transporter, BCCT	B0684_RS03265	0.27	2.04E-03	F468_RS0101745	-0.42	3.01E-03
Betaine/carnitine/choline transporter, BCCT	B0684_RS10705	-0.04	7.17E-01	F468_RS0105750	-0.13	1.87E-01
Glycine betaine/proline ABC transporter ATP-binding protein	B0684_RS06805	0.18	3.66E-02			
Glycine betaine/proline ABC transporter permease protein	B0684_RS06810	0.14	1.32E-01			
Glycine betaine/proline ABC transporter substrate-binding protein	B0684_RS06815	0.42	4.80E-06			
**Glycine betaine de novo synthesis:**						
Glycine/sarcosine N-methyltransferase	B0684_RS10385	0.28	8.48E-03	F468_RS0101770	0.69	1.31E-07
Sarcosine/dimethylglycine N-methyltransferase	B0684_RS10390	0.15	1.34E-01	F468_RS0101775	0.89	2.30E-12
**Anti-freeze protein:**						
Hypothetical protein (Antifreeze-like protein)	B0684_RS01375	1.02	1.14E-31			
**Spermidine synthesis:**						
Polyamine aminopropyltransferase (spermidine synthase)	B0684_RS08940	0.41	9.54E-05	F468_RS0101860	1.52	5.96E-23
S-adenosylmethionine decarboxylase (*spe* D)	B0684_RS12725	0.28	9.61E-04	F468_RS0109655	1.44	5.86E-24

(3) Stability of nucleic acids and proteins						
**Nucleotide synthesis and repair:**						
Adenylate kinase	B0684_RS13090	1.37	5.15E-32	F468_RS0106025	0.58	1.04E-07
Nucleoside-diphosphate kinase	B0684_RS06095	1.33	2.08E-23	F468_RS0104625	1.14	4.93E-20
Non-canonical purine NTP pyrophosphatase, RdgB/HAM1 family	B0684_RS07385	1.05	2.70E-06	F468_RS0100100	0.74	2.58E-13
Translesion DNA synthesis-associated protein (*imu* A)	B0684_RS04595	1.03	1.06E-02	F468_RS0106750	0.62	1.06E-02
DUF1249 domain-containing protein	B0684_RS06150	0.10	3.12E-01	F468_RS0104685	1.04	7.92E-18
(d)CMP kinase	B0684_RS09985	0.58	1.04E-10	F468_RS0108615	1.09	1.05E-30
Carbohydrate kinase family protein	B0684_RS09770	0.40	1.51E-03	F468_RS0105570	1.08	7.13E-11
Adenylosuccinate lyase	B0684_RS10220	0.57	3.10E-09	F468_RS0108375	1.05	6.63E-08
**Transcriptional regulator:**						
RNA polymerase-binding protein (*dks* A)	B0684_RS05535	1.14	1.20E-18	F468_RS0111545	0.91	1.69E-04
Transcriptional regulator (*mra* Z)	B0684_RS04885	-0.36	2.32E-03	F468_RS0110370	1.22	1.52E-38
Transcriptional regulator				F468_RS0110840	1.12	1.46E-14
Crp/Fnr family transcriptional regulator	B0684_RS01005	-1.60	1.52E-19	F468_RS0107830	-0.41	4.41E-05
DeoR family transcriptional regulator	B0684_RS07975	-1.19	4.96E-14	F468_RS0107720	-0.71	5.27E-04
DksA/TraR family C4-type zinc finger protein	B0684_RS04435	-1.04	6.42E-08	F468_RS0107965	-0.91	2.50E-02
AraC family transcriptional regulator	B0684_RS10575	-1.41	1.92E-07	F468_RS0109795	-1.57	1.24E-09
Helix-turn-helix transcriptional regulator				F468_RS13750	-1.44	7.24E-05
Hydrogen peroxide-inducible genes activator	B0684_RS10475	0.18	3.60E-02	F468_RS0109730	-1.38	1.54E-43
Transcriptional regulator	B0684_RS01470	-0.70	1.15E-12	F468_RS0112525	-1.19	6.29E-20
**Transcription and RNA stability:**						
Cold-shock protein	B0684_RS00095	0.08	7.57E-01	F468_RS0100555	0.68	6.63E-05
Cold-shock protein	B0684_RS07170	0.08	7.77E-01	F468_RS0107635	-1.30	3.53E-02
RNA helicase	B0684_RS02505	2.64	2.55E-26	F468_RS0107385	2.50	4.68E-68
Ribonuclease PH	B0684_RS07380	1.16	1.60E-13	F468_RS0100105	1.64	2.58E-13
HU family DNA-binding protein	B0684_RS07030	1.03	1.16E-20	F468_RS0103800	0.19	1.08E-01
Transcription termination/antitermination protein (*nus* A)	B0684_RS11075	0.79	1.87E-10	F468_RS0104590	1.03	2.61E-14

**tRNA:**						
tRNA-Val (TAC)	B0684_RS07035	2.49	3.00E-06	F468_RS0103805	2.75	1.09E-12
tRNA-Ile (GAT)	B0684_RS11475	2.06	3.80E-04	F468_RS0102920	3.38	5.05E-13
tRNA-Leu (GAG)	B0684_RS10985	1.71	3.52E-05	F468_RS0104500	1.87	3.70E-04
tRNA-Leu (CAG)	B0684_RS11900	1.25	6.39E-07	F468_RS0106215	2.66	2.26E-04
tRNA-Leu (TAG)	B0684_RS07005	0.69	1.85E-02	F468_RS0103775	3.25	1.66E-06
tRNA-Trp (CCA)	B0684_RS13275	1.67	5.07E-03	F468_RS0113565	3.56	2.99E-18
tRNA-Thr (GGT)	B0684_RS03905	1.57	2.22E-08	F468_RS0110515	1.43	2.42E-06
tRNA-His (GTG)	B0684_RS06570	1.52	1.06E-02	F468_RS0103980	0.61	1.52E-03
tRNA-Asp (GTC)	B0684_RS07040	1.37	3.24E-09	F468_RS0103810	0.39	1.72E-01
tRNA-Gln (CTG)	B0684_RS10835	1.23	2.02E-13	F468_RS0103620	1.82	1.21E-10
tRNA-Gln (TTG)	B0684_RS13530	-0.18	4.32E-01	F468_RS0102605	1.60	3.91E-13
tRNA-Pro (TGG)	B0684_RS06560	1.16	3.20E-03	F468_RS0103970	2.00	1.96E-03
tRNA-Ser (GGA)	B0684_RS12315	0.00	9.89E-01	F468_RS0104445	2.21	8.77E-06
tRNA-Glu (TTC)	B0684_RS05995	0.81	3.72E-05	F468_RS0105050	2.17	3.51E-20
tRNA-Arg (TCT)	B0684_RS06565	-0.04	8.88E-01	F468_RS0103975	1.47	3.27E-15
tRNA-Gly (TCC)	B0684_RS03910	0.82	9.21E-16	F468_RS0110510	1.35	2.89E-07
tRNA-Tyr (GTA)	B0684_RS03915	0.07	9.00E-01	F468_RS0110505	1.32	3.73E-03
tRNA-Met (CAT)	B0684_RS09265	NA	NA	F468_RS0102185	1.28	1.40E-02
tRNA-Lys (CTT)	B0684_RS06575	0.89	5.09E-02	F468_RS0103985	1.14	1.60E-03
tRNA-Asn (GTT)	B0684_RS10060	0.65	2.08E-01	F468_RS0108540	1.12	1.33E-03
tRNA-Ala (CGC)	B0684_RS00580	-1.67	1.90E-02	F468_RS0112185	-1.21	6.66E-06
**tRNA synthesis:**						
tRNA-guanine(34) transglycosylase	B0684_RS02185	1.31	5.66E-52	F468_RS0108225	1.08	3.73E-10
tRNA preQ1(34) S-adenosylmethionine ribosyltransferase-isomerase QueA	B0684_RS02190	1.05	3.32E-26	F468_RS0108220	1.42	6.76E-33
tRNA glutamyl-Q(34) synthetase GluQRS	B0684_RS05530	1.12	1.52E-13	F468_RS0111540	0.96	3.85E-04
Hypothetical protein (Ala-tRNA(Pro) deacylase)	B0684_RS04470	-1.02	1.74E-04	F468_RS0107980	-1.50	5.43E-03
tRNA threonylcarbamoyl adenosine modification protein	B0684_RS12220	0.83	3.09E-09	F468_RS0107250	1.24	3.12E-29
tRNA (cytidine(34)-2'-O)-methyltransferase	B0684_RS03080	0.32	1.54E-01	F468_RS0101125	1.39	2.76E-13

tRNA (cytidine32/uridine32-2'-O)-methyltransferase	B0684_RS06045	0.58	4.02E-06	F468_RS0111425	1.30	1.32E-35
Aminoacyl-tRNA hydrolase	B0684_RS13510	0.52	7.83E-10	F468_RS0102620	1.22	3.04E-20
tRNA threonylcarbamoyladenosine biosynthesis protein RimN	B0684_RS08800	0.39	5.93E-03	F468_RS0110030	1.05	5.15E-12
**rRNA and ribosomal proteins:**						
5S ribosomal RNA	B0684_RS11460	1.06	2.14E-02	F468_RS0102935	2.50	7.09E-06
50S ribosomal protein L28	B0684_RS07105	1.20	5.54E-01	F468_RS0105925	0.89	7.91E-15
50S ribosomal protein L33	B0684_RS07100	1.24	1.48E-08	F468_RS0105920	1.37	6.68E-22
30S ribosomal protein S9	B0684_RS10830	0.97	3.72E-15	F468_RS0103615	1.48	5.96E-11
30S ribosomal protein S13	B0684_RS12450	0.31	3.60E-03	F468_RS0105215	1.16	1.06E-07
30S ribosomal protein S19	B0684_RS12540	0.80	2.12E-07	F468_RS0105125	1.07	4.22E-08
30S ribosomal protein S20	B0684_RS02900	0.37	1.69E-02	F468_RS0101385	1.40	6.24E-09
50S ribosomal protein L13	B0684_RS10825	0.91	2.28E-06	F468_RS0103610	1.53	4.73E-15
50S ribosomal protein L24	B0684_RS12505	0.67	2.24E-06	F468_RS0105160	1.03	3.41E-20
50S ribosomal protein L25	B0684_RS13515	0.59	1.77E-07	F468_RS0102615	1.18	9.79E-19
50S ribosomal protein L27	B0684_RS09560	0.57	9.63E-04	F468_RS0105865	1.15	8.71E-19
**Translation:**						
Translation initiation factor IF-1	B0684_RS10160	0.12	2.15E-01	F468_RS0108435	1.37	4.90E-15
Translational GTPase (*typ* A)	B0684_RS08955	1.18	2.78E-19	F468_RS0101875	1.28	1.90E-15
50S ribosomal protein L3 N(5)-glutamine methyltransferase	B0684_RS06940	0.13	2.71E-01	F468_RS0106690	1.12	5.45E-28
Redox-regulated Ribosome-binding ATPase (*ych* F)	B0684_RS13505	0.31	2.25E-04	F468_RS0102625	1.12	2.08E-15
**Amino acid synthesis:**						
Phosphoserine phosphatase	B0684_RS04025	1.12	4.56E-42			
Asp-tRNA(Asn)/Glu-tRNA(Gln) amidotransferase subunit (*gat* A)	B0684_RS11775	1.27	2.33E-27	F468_RS0102545	1.05	1.03E-16
Protein-L-isoaspartate O-methyltransferase	B0684_RS13565	0.78	2.64E-10	F468_RS0109190	1.53	2.03E-23
Cysteine synthase (*cys* M)	B0684_RS05550	0.61	4.58E-12	F468_RS0103885	1.08	3.95E-29
ATP phosphoribosyltransferase	B0684_RS05065	0.68	3.43E-16	F468_RS0102785	1.07	2.80E-28
Methionine aminopeptidase, type I (EC 3.4.11.18)	B0684_RS01090	-0.81	8.39E-24	F468_RS0113245	-1.42	1.42E-54
**ncRNA:**						

RtT sRNA	B0684_RS13525	1.83	8.65E-03	F468_RS14055	3.13	4.62E-19
RtT sRNA				F468_RS14105	3.07	3.51E-19
RtT sRNA				F468_RS14450	1.46	7.77E-19
RtT sRNA	B0684_RS08095	0.55	2.97E-01	F468_RS14455	2.27	6.49E-07
Signal recognition particle sRNA small type ffs SRP RNA	B0684_RS12085	1.14	9.63E-02	F468_RS14145	1.54	2.11E-02
RNase P RNA component class A (Rnase P RNA)	B0684_RS04890	-1.58	4.44E-50	F468_RS13915	-0.83	7.16E-03
Transfer-messenger RNA ssrA (tmRNA)	B0684_RS01755	-0.70	5.13E-14	F468_RS14215	-1.06	1.62E-10
**Chaperons:**						
Co-chaperone (*gro* ES)	B0684_RS11635	1.61	4.91E-26	F468_RS0102330	2.29	8.53E-84
Chaperonin (*gro* EL)	B0684_RS11640	1.19	3.30E-26	F468_RS0102335	1.77	1.88E-39
Hsp20/alpha crystallin family protein	B0684_RS04385	-1.86	4.33E-10	F468_RS0107930	-0.48	9.25E-02
Hsp20/alpha crystallin family protein	B0684_RS07365	-2.18	1.18E-14	F468_RS0100120	-1.12	1.14E-04
Peptidylprolyl isomerase (FKBP-type)	B0684_RS03665	-0.26	2.68E-03	F468_RS0103085	1.06	1.30E-25
Peptidylprolyl isomerase (cyclophilin B-type)	B0684_RS06540	0.47	6.61E-09	F468_RS0106310	1.67	9.53E-35
Peptidyl-prolyl cis-trans isomerase (FKBP-type)				F468_RS0101200	-1.25	1.83E-15
**Posttranslational changes:**						
Protein translocase subunit (*sec* D)	B0684_RS02175	1.04	7.47E-16	F468_RS0108235	1.01	1.97E-18
Preprotein translocase subunit (*yaj* C)	B0684_RS02180	1.22	8.46E-17	F468_RS0108230	1.59	4.31E-35
**(4) Protection against oxidative stress**						
**Antioxidants:**						
Superoxide dismutase	B0684_RS00510	0.36	1.72E-05	F468_RS0112115	0.40	6.32E-04
Catalase HPII	B0684_RS09405	0.15	2.06E-01			
Catalase/peroxidase HPI	B0684_RS06350	-0.36	8.73E-04	F468_RS0105525	0.00	9.93E-01
Peroxiredoxin (peroxidase)	B0684_RS08605	-0.78	5.45E-13	F468_RS0111590	1.83	9.22E-72
Glutathione amide-dependent peroxidase	B0684_RS12080	0.31	2.70E-04	F468_RS0106635	1.09	2.75E-17
Peroxiredoxin (peroxidase)	B0684_RS10080	0.85	4.73E-11	F468_RS0108520	1.61	1.53E-17
Thioredoxin family protein	B0684_RS09045	0.05	6.76E-01	F468_RS0102000	1.05	1.09E-24
Glutathione S-transferase family protein	B0684_RS13470	1.05	1.64E-13	F468_RS0105655	1.17	8.26E-14
**Vitamin B12 synthesis:**						

TonB-dependent receptor (*btu* B)	B0684_RS05865	0.73	1.61E-12	F468_RS0104920	0.75	6.61E-09
ATP:cob(I)alamin adenosyltransferase (*cob* A)	B0684_RS05870	0.54	5.35E-10	F468_RS0104925	0.32	8.68E-03
Cobalamine biosynthesis protein (*cob* N)	B0684_RS05875	1.67	4.31E-51	F468_RS0104930	0.75	1.51E-06
Flagellar motor protein (*mot* A)	B0684_RS05880	1.97	3.45E-21	F468_RS0104935	1.25	2.03E-23
Hypothetical protein	B0684_RS05885	1.83	5.93E-12	F468_RS0104940	1.11	1.23E-05
Cob(I)yric acid a,c-diamide adenosyltransferase (*cob* A/*cob* O)	B0684_RS05890	1.33	4.59E-48	F468_RS0104945	0.79	5.26E-04
Cobyrinate a,c-diamide synthase (*cob* B)	B0684_RS05895	1.63	7.92E-44	F468_RS0104950	0.83	3.65E-18
Uroporphyrinogen-III C-methyltransferase (*cob* A)	B0684_RS05900	1.40	9.42E-17	F468_RS0104955	0.97	1.46E-18
Cobalt-precorrin-5B (C(1))-methyltransferase (*cob* F)	B0684_RS05905	1.25	1.56E-18	F468_RS0104960	1.10	6.72E-23
ABC transporter ATP-binding protein (*btu* D)	B0684_RS05910	1.36	1.15E-30	F468_RS0104965	1.01	1.20E-21
ABC transporter permease (*btu* C)	B0684_RS05915	1.34	1.61E-15	F468_RS0104970	0.86	1.34E-12
Precorrin-3B synthase (*cob* S)	B0684_RS05920	1.23	1.09E-16	F468_RS0104975	0.96	1.33E-14
Precorrin-8X methylmutase (*cob* H)	B0684_RS05925	1.49	7.45E-25	F468_RS0104980	0.64	4.20E-06
Precorrin-2 C(20)-methyltransferase (*cob* I)	B0684_RS05930	1.39	2.42E-30	F468_RS0104985	0.70	6.69E-11
Precorrin-3B C(17)-methyltransferase (*cob* J)	B0684_RS05935	1.17	3.72E-20	F468_RS0104990	0.53	4.96E-06
Cobalt-precorrin-6A reductase (*cob* K)	B0684_RS05940	1.03	1.67E-12	F468_RS0104995	1.08	8.77E-06
Bifunctional cobalt-precorrin-7 (C(5))-methyltransferase/ cobalt-precorrin-6B (C(15))-methyltransferase (*cob* L)	B0684_RS05945	1.23	1.72E-11	F468_RS0105000	1.13	2.00E-07
Hypothetical protein (Cobalt-precorrin 5A hydrolase) (*cob* E)	B0684_RS05950	0.47	3.27E-02	F468_RS0105005	1.43	4.39E-03
Precorrin-4 C(11)-methyltransferase (*cob* M)	B0684_RS05955	0.08	5.83E-01	F468_RS0105010	0.85	7.93E-10
**(5) Sulfur, carbon and energy metabolism**						
**Sulfur metabolism:**						
Formate dehydrogenase (Sulfite-oxidizing enzyme) (*soe* A1)				F468_RS0101160	-2.51	2.08E-04
4Fe-4S dicluster domain-containing protein (Sulfite-oxidizing enzyme) (*soe* B1)				F468_RS0101155	-2.36	1.75E-04
DMSO reductase (Sulfite-oxidizing enzyme) (*soe* C1)				F468_RS0101150	-1.88	5.62E-08
Formate dehydrogenase (Sulfite-oxidizing enzyme) (*soe* A2)	B0684_RS03120	0.68	2.30E-11	F468_RS0101595	2.09	6.19E-46

4Fe-4S dicluster domain-containing protein (Sulfite-oxidizing enzyme) (*soe* B2)	B0684_RS03125	0.48	5.74E-06	F468_RS0101600	0.98	3.61E-04
DMSO reductase (Sulfite-oxidizing enzyme (*soe* C2)	B0684_RS03130	0.31	2.84E-03	F468_RS0101605	0.80	4.66E-03
Sulfite oxidase (Sulfite dehydrogenase (cytochrome) subunit) (*sor* A)	B0684_RS12250	-1.03	1.24E-02	F468_RS0104385	-3.36	4.19E-07
Hypothetical protein (Cytochrome c, mono- and diheme variants) (*sor* B)	B0684_RS12255	-0.89	5.81E-02	F468_RS0104390	-1.57	2.05E-08
Adenylyl-sulfate reductase subunit alpha (*apr* A)				F468_RS0101190	-1.88	7.49E-10
Adenylyl-sulfate reductase subunit beta (*apr* B)				F468_RS0101185	-1.79	1.52E-07
Sulfate adenylyltransferase (sat)				F468_RS0101175	-1.27	2.72E-09
Hypothetical protein (Sulfide dehydrogenase (flavocytochrome c), flavoprotein subunit) (*fcc* A)	B0684_RS09070	-1.16	4.16E-05	F468_RS0102025	-2.29	5.65E-42
Sulfide dehydrogenase (Flavocytochrome c), cytochrome c subunit (EC 1.8.2.3) (*fcc* B)	B0684_RS09065	-1.26	6.98E-13	F468_RS0102020	-2.01	8.71E-19
Hypothetical protein (Sulfide dehydrogenase (flavocytochrome c), flavoprotein subunit) (*fcc* A)				F468_RS0107475	-2.30	1.08E-35
Sulfide dehydrogenase (Flavocytochrome c), cytochrome c subunit (EC 1.8.2.3) (*fcc* B)				F468_RS0107480	-2.38	3.42E-33
Sulfide dehydrogenase (flavocytochrome c), cytochrome c subunit (EC 1.8.2.3) (*fcc* B)				F468_RS0112815	-0.40	2.44E-04
Sulfur oxidation c-type cytochrome (*sox* A)	B0684_RS03640	0.07	7.91E-01	F468_RS0103065	-0.69	2.61E-06
Thiosulfohydrolase (*sox* B)	B0684_RS01835	-1.09	4.66E-30	F468_RS0108920	0.52	5.13E-04
Thiosulfohydrolase (*sox* B)	B0684_RS06690	0.35	5.16E-04	F468_RS0104100	-0.25	4.61E-02
Sulfur oxidation c-type cytochrome (*sox* X)	B0684_RS03650	0.12	6.62E-01	F468_RS0103075	-0.60	3.60E-06
Sulfur oxidation c-type cytochrome (*sox* X)	B0684_RS03645	-0.12	6.63E-01	F468_RS0103070	-0.80	1.84E-08
Thiosulfate oxidation carrier protein (*sox* Y)	B0684_RS07635	-0.04	8.51E-01	F468_RS0101475	-0.50	3.88E-04
Thiosulfate oxidation carrier complex protein (*sox* Z)	B0684_RS07630	0.02	9.34E-01	F468_RS13705	-0.12	5.24E-01
**Carbon metabolism:**						
Ribulose-bisphosphate carboxylase large subunit rbcL (Rubisco large subunit)	B0684_RS08280	1.23	5.09E-34	F468_RS0111615	0.78	3.41E-08

Ribulose bisphosphate carboxylase small subunit (Rubisco small subunit)	B0684_RS08285	1.03	5.08E-27	F468_RS0111620	0.34	1.63E-01
BMC domain-containing protein (subunit of the carboxysome)	B0684_RS08310	2.06	3.03E-93	F468_RS0111645	0.55	5.46E-04
BMC domain-containing protein (subunit of the carboxysome)				F468_RS0111650	0.38	8.81E-04
BMC domain-containing protein (subunit of the carboxysome)	B0684_RS08315	0.66	2.84E-16	F468_RS0111655	-0.09	5.18E-01
Carbon storage regulator (*csr* A)				F468_RS0109120	1.83	2.20E-25
**Energy metabolism:**						
NADH-quinone oxidoreductase subunit (*nuo* A)	B0684_RS10990	0.72	6.21E-04	F468_RS0104505	0.32	1.07E-02
NADH-quinone oxidoreductase subunit (*nuo* B)	B0684_RS10995	0.62	6.49E-07	F468_RS0104510	0.18	1.69E-01
NADH-quinone oxidoreductase subunit (*nuo* C)	B0684_RS11000	0.51	1.76E-08	F468_RS0104515	0.26	3.65E-02
NADH-quinone oxidoreductase subunit (*nuo* D)	B0684_RS11005	0.43	1.77E-03	F468_RS0104520	0.19	1.12E-01
NADH-dependent oxidoreductase subunit (*nuo* E)	B0684_RS11010	0.42	1.83E-04	F468_RS0104525	-0.29	5.42E-03
NADH oxidoreductase (quinone) subunit (*nuo* F)	B0684_RS11015	0.29	6.21E-02	F468_RS0104530	-0.14	2.28E-01
NADH-quinone oxidoreductase subunit (*nuo* G)	B0684_RS11020	0.15	1.22E-01	F468_RS0104535	-0.27	2.49E-02
NADH-quinone oxidoreductase subunit (*nuo* H)	B0684_RS11025	0.20	1.80E-02	F468_RS0104540	-0.21	3.02E-02
NADH-quinone oxidoreductase subunit (*nuo* I)	B0684_RS11030	0.25	2.25E-03	F468_RS0104545	0.17	1.69E-01
NADH-quinone oxidoreductase subunit (*nuo* J)	B0684_RS11035	0.21	1.36E-02	F468_RS0104550	-0.28	4.01E-03
NADH-quinone oxidoreductase subunit (*nuo* K)	B0684_RS11040	0.91	3.47E-20	F468_RS0104555	0.04	7.12E-01
NADH-quinone oxidoreductase subunit (*nuo* L)	B0684_RS11045	1.01	1.61E-23	F468_RS0104560	-0.10	3.93E-01
NADH-quinone oxidoreductase subunit (*nuo* M)	B0684_RS11050	0.95	2.02E-16	F468_RS0104565	-0.13	2.62E-01
NADH-quinone oxidoreductase subunit (*nuo* N)	B0684_RS11055	0.77	1.94E-18	F468_RS0104570	-0.19	5.52E-02
F0F1 ATP synthase subunit epsilon (*atp* C)	B0684_RS07725	0.36	4.67E-02	F468_RS0100750	0.59	1.17E-02
F0F1 ATP synthase subunit beta (*atp* D)	B0684_RS07730	0.49	3.46E-03	F468_RS0100755	0.70	8.19E-05
F0F1 ATP synthase subunit gamma (*atp* G)	B0684_RS07735	0.44	6.04E-02	F468_RS0100760	0.49	2.14E-02
F0F1 ATP synthase subunit alpha (*atp* A)	B0684_RS07740	0.54	8.30E-04	F468_RS0100765	0.84	3.33E-06
F0F1 ATP synthase subunit delta (*atp* H)	B0684_RS07745	0.55	2.45E-04	F468_RS0100770	1.10	6.30E-16
F0F1 ATP synthase subunit B (*atp* F)	B0684_RS07750	0.51	7.39E-04	F468_RS0100775	0.97	8.84E-05
F0F1 ATP synthase subunit C (*atp* E)	B0684_RS07755	1.30	6.36E-11	F468_RS0100780	1.01	2.36E-08

F0F1 ATP synthase subunit A (*atp* B)	B0684_RS07760	0.81	9.50E-16	F468_RS0100785	1.18	9.59E-20
Na+/H+ antiporter	B0684_RS05655	1.04	2.22E-28	F468_RS0104710	0.87	8.18E-18
**(6) Chemotaxis and motility**						
**Chemotaxis:**						
Methyl-accepting chemotaxis protein	B0684_RS11310	2.46	6.91E-66	F468_RS0111155	1.48	7.89E-06
Methyl-accepting chemotaxis protein	B0684_RS00520	2.33	3.86E-59	F468_RS0112125	1.34	7.53E-22
Methyl-accepting chemotaxis protein	B0684_RS01475	1.77	2.23E-34			
Methyl-accepting chemotaxis protein	B0684_RS00015	1.43	9.23E-64	F468_RS0100630	0.84	1.99E-04
Methyl-accepting chemotaxis protein	B0684_RS02500	0.96	3.15E-15	F468_RS0107390	1.68	6.94E-09
Methyl-accepting chemotaxis protein	B0684_RS03415	-0.76	1.39E-05	F468_RS0107460	-3.25	3.06E-23
**Signalling:**						
GGDEF domain-containing protein	B0684_RS10480	1.30	2.18E-08	F468_RS0109735	-3.03	1.19E-52
GGDEF domain-containing protein	B0684_RS11230	-1.48	6.82E-33	F468_RS0111175	-0.52	2.18E-07
GGDEF domain-containing protein	B0684_RS04410	-1.15	1.52E-34			
GGDEF domain-containing protein	B0684_RS01210	-1.10	3.04E-16	F468_RS0113365	-1.17	6.40E-25
GGDEF domain-containing protein	B0684_RS04320	-1.05	6.74E-10			
Two-component sensor histidine kinase	B0684_RS06160	-0.32	1.19E-02	F468_RS0110750	-1.13	3.72E-24
Two-component system response regulator	B0684_RS06155	-0.24	9.31E-02	F468_RS0110755	-0.86	3.47E-17
**Flagella synthesis:**						
c-di-GMP-binding flagellar brake protein (*ycg* R)	B0684_RS00785	1.21	1.85E-44	F468_RS0111980	-0.95	1.17E-12
Flagellar synthesis protein (*flg* N)	B0684_RS00790	1.37	1.31E-27	F468_RS0111985	-1.99	1.15E-33
Flagellar biosynthesis anti-sigma factor (*flg* M)	B0684_RS00795	1.73	6.90E-32	F468_RS0111990	-2.17	9.24E-59
Flagella basal body P-ring formation protein (*flg* A)	B0684_RS00800	0.76	9.32E-08	F468_RS0111995	-3.46	4.37E-67
Two-component system, chemotaxis family, response regulator (*che* V)	B0684_RS00805	0.35	3.40E-05	F468_RS0112000	0.17	1.03E-01
Chemotaxis protein methyltransferase (*che* R)	B0684_RS00810	0.41	5.31E-07	F468_RS0112005	0.25	1.06E-02
Flagellar basal body rod protein (*flg* B)	B0684_RS00815	1.46	3.33E-05	F468_RS0112010	-2.52	2.93E-38
Flagellar basal body rod protein (*flg* C)	B0684_RS00825	1.69	1.22E-06	F468_RS0112015	-2.53	1.44E-44

Hypothetical protein (Flagellar basal-body rod modification protein) (*flg* D)	B0684_RS00830	1.67	3.51E-08	F468_RS0112020	-2.62	1.41E-57
Flagellar hook protein (*flg* E)	B0684_RS00835	1.53	7.33E-14	F468_RS0112025	-2.27	7.64E-48
Flagellar basal-body rod protein (*flg* F)	B0684_RS00840	1.50	8.21E-10	F468_RS0112030	-2.43	4.08E-44
Flagellar basal-body rod protein (*flg* G)	B0684_RS00845	1.59	4.56E-11	F468_RS0112035	-2.58	5.18E-50
Flagellar basal body L-ring protein (*flg* H)	B0684_RS00850	1.45	1.11E-08	F468_RS0112040	-2.78	2.36E-83
Flagellar basal body P-ring protein (*flg* I)	B0684_RS00855	1.35	1.86E-10	F468_RS0112045	-2.87	4.45E-78
Flagellar assembly peptidoglycan hydrolase (*flg* J)	B0684_RS00860	1.15	1.25E-05	F468_RS0112050	-2.86	8.66E-64
Flagellar hook-associated protein (*flg* K)	B0684_RS00865	1.53	3.43E-20	F468_RS0112055	-2.44	2.05E-65
Flagellar hook-associated protein 3 (*flg* L)	B0684_RS00870	1.26	1.66E-20	F468_RS0112060	-2.45	8.35E-82
Hypothetical protein	B0684_RS00875	0.92	6.85E-18	F468_RS0112065	-1.94	1.12E-96
Flagellin (*fli* C)	B0684_RS01335	2.45	1.62E-32	F468_RS14355	2.21	1.38E-21
Flagellar biosynthesis protein (*fla* G)	B0684_RS01340	1.92	4.48E-56	F468_RS14360	1.85	1.38E-20
Flagellin (*fli* C)				F468_RS0112440	1.62	2.84E-06
Hypothetical protein (flagellar hook-associated protein 2) (*fli* D)	B0684_RS01345	1.50	9.68E-58	F468_RS14365	1.98	1.38E-03
Flagella export chaperone (*fli* S)	B0684_RS01350	1.41	8.98E-68	F468_RS14370	1.33	3.34E-03
Flagellar protein (*fli* T)	B0684_RS01355	0.97	5.00E-24	F468_RS0112455	-0.04	8.03E-01
Flagellar motor protein MotB	B0684_RS01610	0.84	1.25E-23	F468_RS0108765	-0.39	1.33E-04
Flagellar motor protein MotC	B0684_RS01615	0.60	3.41E-09	F468_RS0108770	-0.99	6.79E-09
Chemotaxis response regulator protein-glutamate methylesterase	B0684_RS01620	0.75	1.53E-19	F468_RS0108775	-0.78	5.16E-14
Chemotaxis protein CheA	B0684_RS01625	0.76	1.48E-17	F468_RS0108780	-0.57	3.48E-06
Protein phosphatase (*che* Z)	B0684_RS01630	0.40	9.52E-07	F468_RS0108785	-1.21	6.09E-28
Chemotaxis protein (*che* Y)	B0684_RS01635	0.33	7.32E-05	F468_RS0108790	-0.92	8.43E-11
RNA polymerase sigma factor (*fli* A)	B0684_RS01640	1.04	8.06E-17	F468_RS0108795	-0.75	3.14E-15
Flagellar biosynthesis protein (*flh* G)	B0684_RS01645	0.81	2.91E-12	F468_RS0108800	-1.35	9.94E-39
Flagellar biosynthesis protein (*flh* F)	B0684_RS01650	0.82	9.64E-09	F468_RS0108805	-1.62	1.60E-45
Flagellar biosynthesis protein (*flh* A)	B0684_RS01655	1.75	9.34E-11	F468_RS0108810	-1.56	1.14E-19
Flagellar biosynthesis protein (*flh* B)	B0684_RS01660	1.66	1.71E-24	F468_RS0108815	-1.42	5.09E-23
Flagellar biosynthetic protein (*fli* R)	B0684_RS01665	1.79	1.67E-10	F468_RS0108820	-1.53	4.15E-31

Flagellar biosynthetic protein (*fli* Q)	B0684_RS01670	1.71	2.27E-09	F468_RS0108825	-1.17	5.78E-09
Flagellar biosynthetic protein (*fli* P)	B0684_RS01675	1.76	2.26E-10	F468_RS0108830	-1.33	3.42E-20
Flagellar biosynthetic protein (*fli* O)	B0684_RS01680	1.74	1.97E-10	F468_RS0108835	-1.31	6.94E-12
Flagellar motor switch protein (*fli* N)	B0684_RS01685	2.17	1.53E-10	F468_RS0108840	-1.68	1.56E-20
Flagellar motor switch protein (*fli* M)	B0684_RS01690	1.86	5.37E-16	F468_RS0108845	-1.78	4.30E-17
Hypothetical protein (Flagellar protein) (*fli* L)	B0684_RS01695	1.95	6.58E-13	F468_RS0108850	-2.12	3.21E-26
Flagellar hook-length control protein (*fli* K)	B0684_RS01700	0.81	1.81E-04	F468_RS0108855	-2.95	3.69E-132
Flagellar export protein (*fli* J)	B0684_RS01705	-0.08	5.75E-01	F468_RS0108860	-1.25	1.23E-43
Flagellum-specific ATP synthase (*fli* I)	B0684_RS01710	0.20	9.98E-02	F468_RS0108865	-1.19	6.10E-31
Hypothetical protein (Flagellar assembly protein) (*fli* H)	B0684_RS01715	0.05	8.15E-01	F468_RS0108870	-1.63	2.79E-51
Flagellar motor switch protein (*fli* G)	B0684_RS01720	0.27	3.46E-02	F468_RS0108875	-1.39	1.04E-38
Flagellar M-ring protein (*fli* F)	B0684_RS01725	1.09	5.88E-08	F468_RS0108880	-1.62	7.04E-36
Flagellar hook-basal body complex protein (*fli* E)	B0684_RS01730	1.51	5.08E-07	F468_RS0108885	-1.58	2.31E-25
Sigma-54-dependent Fis family transcriptional regulator	B0684_RS01735	0.50	1.39E-06	F468_RS0108890	-1.55	5.77E-20
**Pilus synthesis:**						
Type IV pilus assembly protein (*pil* E)				F468_RS0101880	0.53	5.33E-03
Hypothetical protein				F468_RS0101885	0.36	4.99E-02
Type IV pilus assembly protein (*pil* Y1)				F468_RS0101890	0.72	4.11E-04
Type IV pilus assembly protein (*pil* X)				F468_RS0101895	1.02	1.53E-12
Type IV pilus assembly protein (*pil* W)				F468_RS0101900	0.74	5.96E-07
Type IV pilus assembly protein (*pil* V)	B0684_RS08960	0.01	9.08E-01	F468_RS0101905	1.24	6.58E-07
type IV fimbrial biogenesis protein (*fim* T)				F468_RS0101910	0.76	5.88E-06
Type IV fimbrial biogenesis protein (*fim* T)	B0684_RS08985	0.05	5.87E-01	F468_RS0101915	-0.17	1.09E-01
Prepilin-type N-terminal cleavage/methylation domain-containing protein				F468_RS13710	-1.13	1.21E-17
Hypothetical protein	B0684_RS08965	0.22	1.17E-02	F468_RS0101925	-1.53	7.28E-30
Type IV pilin N-term methylation site GFxxxE	B0684_RS08970	0.30	5.94E-04	F468_RS0101930	-1.77	1.15E-23
Type IV fimbrial biogenesis protein (*fim* T)	B0684_RS08975	0.41	9.13E-06	F468_RS0101935	-1.76	1.98E-19
Type IV pilus assembly protein (*pil* E)	B0684_RS08980	0.02	8.10E-01	F468_RS0101940	-1.11	1.74E-20
Type IV pilus assembly protein (*pil* A)	B0684_RS10930	0.73	2.74E-07	F468_RS0111320	-1.33	1.46E-22
