# Supplementary material for: Molecular and Physiological Adaptations to Low Temperature in Thioalkalivibrio Strains Isolated from Soda Lakes with Different Temperature Regimes
Source: mSystems. 2021 Apr 27;6(2):e01202-20. doi: 10.1128/mSystems.01202-20 (PMC8092127; doi:10.1128/mSystems.01202-20)
Supplement: TABLE S5 [file msystems.01202-20-st005.pdf]

| Orthogroup | Mean | $b \pm se$ | $P$ | $P_{adj}$ | Protein product |
|---|---|---|---|---|---|
| OG0000407 | 6.47 | -1.90±0.13 | $5.091e-51$ | $1.927e-48$ | co-chaperone GroES |
| OG0001236 | 6.47 | -1.89±0.22 | $4.680e-18$ | $7.703e-17$ | methyl-accepting chemotaxis protein |
| OG0002054 | 7.32 | -1.82±0.11 | $4.466e-60$ | $2.818e-57$ | flagellar biosynthesis protein FlaG;hypothetical protein |
| OG0001542 | 6.83 | -1.74±0.16 | $4.939e-27$ | $1.833e-25$ | methyl-accepting chemotaxis protein |
| OG0001745 | 3.74 | -1.62±0.16 | $7.090e-24$ | $2.033e-22$ | hypothetical protein |
| OG0000691 | 5.03 | -1.57±0.35 | $7.431e-06$ | $2.364e-05$ | transporter;TolC family protein |
| OG0000988 | 4.28 | -1.55±0.15 | $5.776e-24$ | $1.682e-22$ | flagellar motor protein MotA |
| OG0001237 | 5.65 | -1.50±0.30 | $3.716e-07$ | $1.430e-06$ | hypothetical protein |
| OG0001095 | 9.03 | -1.42±0.11 | $7.542e-38$ | $5.949e-36$ | chaperonin GroEL |
| OG0000989 | 3.31 | -1.38±0.17 | $1.340e-16$ | $1.812e-15$ | hypothetical protein;DUF2149 domain-containing protein |
| OG0000267 | 6.19 | -1.35±0.23 | $2.454e-09$ | $1.263e-08$ | rhodanese-like domain-containing protein |
| OG0000499 | 6.18 | -1.35±0.10 | $7.485e-41$ | $7.457e-39$ | preprotein translocase subunit YajC |
| OG0001936 | 5.52 | -1.34±0.10 | $5.043e-38$ | $4.151e-36$ | ribonuclease PH |
| OG0000977 | 6.50 | -1.32±0.09 | $1.083e-54$ | $5.124e-52$ | hypothetical protein |
| OG0000504 | 5.58 | -1.26±0.17 | $5.041e-13$ | $4.430e-12$ | methyl-accepting chemotaxis protein |
| OG0000225 | 6.67 | -1.25±0.10 | $7.227e-34$ | $4.275e-32$ | 50S ribosomal protein L33 |
| OG0000897 | 4.82 | -1.23±0.10 | $4.801e-33$ | $2.456e-31$ | hypothetical protein |
| OG0001753 | 5.53 | -1.20±0.18 | $1.226e-11$ | $8.690e-11$ | phosphatase |
| OG0000991 | 5.68 | -1.19±0.14 | $1.995e-18$ | $3.312e-17$ | cobyrinate a,c-diamide synthase |
| OG0000525 | 5.62 | -1.18±0.08 | $1.988e-44$ | $3.763e-42$ | 3-hydroxyacyl-[acyl-carrier-protein] dehydratase FabA |
| OG0001354 | 4.82 | -1.17±0.13 | $1.774e-20$ | $3.713e-19$ | DUF1244 domain-containing protein |
| OG0000283 | 7.35 | -1.17±0.13 | $1.476e-18$ | $2.473e-17$ | 30S ribosomal protein S9 |
| OG0000510 | 6.28 | -1.17±0.09 | $2.659e-41$ | $2.960e-39$ | nucleoside-diphosphate kinase |
| OG0001681 | 5.28 | -1.17±0.09 | $1.235e-39$ | $1.118e-37$ | tRNA preQ1(34) S-adenosylmethionine ribosyltransferase-isomerase QueA |
| OG0002027 | 6.91 | -1.17±0.08 | $3.646e-44$ | $6.275e-42$ | NAD(P) transhydrogenase subunit alpha |
| OG0000284 | 7.25 | -1.16±0.15 | $2.183e-15$ | $2.667e-14$ | 50S ribosomal protein L13 |
| OG0001355 | 4.17 | -1.16±0.12 | $4.133e-23$ | $1.134e-21$ | rod shape-determining protein MreD |
| OG0001978 | 7.30 | -1.15±0.16 | $3.988e-13$ | $3.578e-12$ | cobalamin biosynthesis protein CobN |
| OG0000266 | 7.92 | -1.14±0.09 | $5.397e-36$ | $3.930e-34$ | sulfurtransferase TusA family protein |
| OG0000992 | 4.94 | -1.13±0.11 | $4.863e-23$ | $1.315e-21$ | uroporphyrinogen-III C-methyltransferase |
| OG0001680 | 6.02 | -1.12±0.09 | $1.240e-39$ | $1.118e-37$ | tRNA-guanine(34) transglycosylase;tRNA guanosine(34) transglycosylase Tgt |
| OG0000789 | 9.42 | -1.11±0.10 | $9.343e-29$ | $4.020e-27$ | glutamate–ammonia ligase |
| OG0000217 | 6.94 | -1.10±0.13 | $6.596e-18$ | $1.058e-16$ | F0F1 ATP synthase subunit C |
| OG0001979 | 4.18 | -1.10±0.13 | $1.618e-17$ | $2.532e-16$ | bifunctional cobalt-precorrin-7 (C(5))-methyltransferase / cobalt-precorrin-6B (C(15))-methyltransferase |
| OG0000993 | 4.81 | -1.10±0.10 | $1.625e-29$ | $7.326e-28$ | cobalt-precorrin-5B (C(1))-methyltransferase |
| OG0000351 | 5.82 | -1.09±0.13 | $3.351e-16$ | $4.406e-15$ | protein-L-isoaspartate O-methyltransferase |
| OG0000981 | 4.98 | -1.07±0.11 | $7.029e-22$ | $1.663e-20$ | hypothetical protein |
| OG0000622 | 4.29 | -1.07±0.11 | $2.522e-22$ | $6.200e-21$ | glutathione S-transferase family protein |
| OG0000374 | 5.41 | -1.05±0.15 | $2.584e-12$ | $2.013e-11$ | hypothetical protein |
| OG0000519 | 7.27 | -1.05±0.13 | $7.411e-17$ | $1.063e-15$ | hypothetical protein |
| OG0000690 | 4.39 | -1.04±0.23 | $8.415e-06$ | $2.633e-05$ | hypothetical protein;copper resistance protein CopC |
| OG0000994 | 4.71 | -1.04±0.10 | $3.301e-25$ | $1.041e-23$ | precorrin-3B synthase |

| Orthogroup | Mean | $b \pm se$ | $P$ | $P_{adj}$ | Protein product |
|---|---|---|---|---|---|
| OG0000995 | 4.61 | -1.02±0.14 | $9.265e-13$ | $7.830e-12$ | precorrin-8X methylmutase |
| OG0001177 | 6.22 | -1.01±0.18 | $1.302e-08$ | $6.102e-08$ | cyclophilin;peptidylprolyl isomerase |
| OG0002082 | 4.28 | -1.01±0.14 | $1.129e-13$ | $1.119e-12$ | tRNA glutamyl-Q(34) synthetase GluQRS |
