# Supplementary material for: Molecular and Physiological Adaptations to Low Temperature in Thioalkalivibrio Strains Isolated from Soda Lakes with Different Temperature Regimes
Source: mSystems. 2021 Apr 27;6(2):e01202-20. doi: 10.1128/mSystems.01202-20 (PMC8092127; doi:10.1128/mSystems.01202-20)
Supplement: TABLE S4 [file msystems.01202-20-st004.pdf]

| Orthogroup | Mean | $b \pm se$ | $P$ | $P_{adj}$ | Protein product |
|---|---|---|---|---|---|
| OG0000755 | 6.90 | 2.28±0.67 | $6.542e-04$ | $1.455e-03$ | efflux RND transporter periplasmic adaptor subunit |
| OG0000759 | 8.76 | 2.25±0.48 | $3.534e-06$ | $1.205e-05$ | hypothetical protein;sulfite oxidase |
| OG0000306 | 6.24 | 2.09±0.40 | $1.378e-07$ | $5.574e-07$ | hypothetical protein;methyl-accepting chemotaxis protein |
| OG0001260 | 6.82 | 2.09±0.32 | $5.557e-11$ | $3.628e-10$ | heme-binding protein |
| OG0000343 | 3.51 | 2.05±0.55 | $2.038e-04$ | $4.986e-04$ | DUF302 domain-containing protein |
| OG0001001 | 7.71 | 1.97±0.30 | $7.663e-11$ | $4.917e-10$ | DUF4399 domain-containing protein |
| OG0000353 | 6.18 | 1.97±0.15 | $1.355e-39$ | $1.166e-37$ | sugar ABC transporter substrate-binding protein |
| OG0000781 | 5.11 | 1.86±0.13 | $3.053e-43$ | $4.445e-41$ | cytochrome d ubiquinol oxidase subunit II |
| OG0001254 | 3.80 | 1.83±0.19 | $1.058e-22$ | $2.635e-21$ | DUF302 domain-containing protein |
| OG0000784 | 5.95 | 1.79±0.13 | $1.846e-42$ | $2.496e-40$ | hypothetical protein;UDP-phosphate galactose phosphotransferase |
| OG0001451 | 6.14 | 1.74±0.56 | $1.963e-03$ | $3.966e-03$ | ParA;hypothetical protein |
| OG0000785 | 6.67 | 1.71±0.24 | $6.313e-13$ | $5.507e-12$ | Hsp20/alpha crystallin family protein |
| OG0000942 | 6.59 | 1.71±0.14 | $1.773e-33$ | $1.017e-31$ | hypothetical protein;NACHT domain-containing protein |
| OG0002070 | 7.07 | 1.69±0.66 | $9.860e-03$ | $1.730e-02$ | hypothetical protein |
| OG0000925 | 4.27 | 1.67±0.38 | $1.003e-05$ | $3.071e-05$ | class I SAM-dependent methyltransferase |
| OG0000751 | 6.14 | 1.67±0.14 | $3.397e-33$ | $1.838e-31$ | GNAT family N-acetyltransferase |
| OG0001927 | 6.89 | 1.64±0.13 | $1.563e-34$ | $9.864e-33$ | hypothetical protein;LPS biosynthesis protein |
| OG0001491 | 5.60 | 1.63±0.10 | $1.597e-62$ | $1.512e-59$ | cytochrome ubiquinol oxidase subunit I |
| OG0001551 | 5.10 | 1.62±0.20 | $1.740e-16$ | $2.336e-15$ | acyl-CoA dehydrogenase |
| OG0001925 | 5.81 | 1.61±0.11 | $9.414e-49$ | $2.970e-46$ | DUF3473 domain-containing protein |
| OG0001301 | 5.90 | 1.60±0.10 | $1.228e-63$ | $2.325e-60$ | PEP-CTERM system histidine kinase PrsK |
| OG0002049 | 6.90 | 1.59±0.12 | $7.474e-41$ | $7.457e-39$ | TIGR03016 family PEP-CTERM system-associated outer membrane protein |
| OG0000439 | 4.35 | 1.58±0.30 | $1.299e-07$ | $5.279e-07$ | hypothetical protein |
| OG0000924 | 5.81 | 1.55±0.17 | $7.380e-19$ | $1.270e-17$ | AraC family transcriptional regulator |
| OG0001090 | 5.56 | 1.48±0.16 | $8.184e-20$ | $1.597e-18$ | serine protease |
| OG0000344 | 7.68 | 1.47±0.48 | $2.236e-03$ | $4.446e-03$ | hypothetical protein |
| OG0002069 | 8.29 | 1.42±0.32 | $1.008e-05$ | $3.083e-05$ | FAD-dependent oxidoreductase;NAD/FAD-binding protein |
| OG0001926 | 6.35 | 1.41±0.13 | $1.579e-25$ | $5.337e-24$ | hypothetical protein;protein tyrosine kinase |
| OG0001073 | 6.73 | 1.40±0.60 | $1.909e-02$ | $3.137e-02$ | flagella basal body P-ring formation protein FlgA |
| OG0000943 | 3.69 | 1.39±0.23 | $2.800e-09$ | $1.425e-08$ | large-conductance mechanosensitive channel protein MscL |
| OG0001555 | 5.34 | 1.37±0.14 | $2.598e-24$ | $7.684e-23$ | hypothetical protein |
| OG0001910 | 6.01 | 1.36±0.11 | $1.452e-34$ | $9.476e-33$ | UDP-N-acetylglucosamine 2-epimerase (non-hydrolyzing) |
| OG0001768 | 6.22 | 1.33±0.32 | $3.671e-05$ | $1.019e-04$ | CBS domain-containing protein |
| OG0002093 | 5.47 | 1.33±0.20 | $5.137e-11$ | $3.365e-10$ | glycerate kinase |
| OG0001519 | 6.14 | 1.32±0.28 | $3.387e-06$ | $1.159e-05$ | hypothetical protein |
| OG0001302 | 4.21 | 1.31±0.26 | $3.115e-07$ | $1.211e-06$ | YdcF family protein |
| OG0000539 | 6.34 | 1.31±0.23 | $7.254e-09$ | $3.512e-08$ | host attachment protein |
| OG0000413 | 5.57 | 1.30±0.20 | $6.149e-11$ | $3.987e-10$ | hypothetical protein |
| OG0000397 | 6.84 | 1.30±0.17 | $3.847e-14$ | $4.024e-13$ | cytochrome c |
| OG0001089 | 5.80 | 1.29±0.11 | $1.354e-32$ | $6.570e-31$ | hypothetical protein;sugar transferase |

| Orthogroup | Mean | $b \pm se$ | $P$ | $P_{adj}$ | Protein product |
|---|---|---|---|---|---|
| OG0000966 | 4.99 | 1.28±0.09 | $1.449e-45$ | $3.918e-43$ | sodium-dependent bicarbonate transport family permease |
| OG0001490 | 5.83 | 1.27±0.09 | $5.297e-45$ | $1.254e-42$ | hypothetical protein;NAD(P)/FAD-dependent oxidoreductase |
| OG0001506 | 6.18 | 1.26±0.18 | $6.752e-13$ | $5.836e-12$ | hypothetical protein |
| OG0001565 | 5.99 | 1.24±0.15 | $2.484e-16$ | $3.289e-15$ | SIMPL domain-containing protein |
| OG0002000 | 4.75 | 1.22±0.27 | $4.405e-06$ | $1.458e-05$ | Hsp20/alpha crystallin family protein |
| OG0001912 | 5.78 | 1.22±0.09 | $1.512e-44$ | $3.179e-42$ | pyridoxal-dependent decarboxylase, exosortase A system-associated |
| OG0000376 | 5.91 | 1.21±0.23 | $8.682e-08$ | $3.620e-07$ | hypothetical protein |
| OG0001356 | 4.68 | 1.21±0.10 | $1.963e-33$ | $1.093e-31$ | hypothetical protein |
| OG0000428 | 7.48 | 1.20±0.25 | $2.303e-06$ | $8.088e-06$ | hypothetical protein |
| OG0001556 | 5.65 | 1.20±0.09 | $8.698e-38$ | $6.586e-36$ | DUF692 domain-containing protein |
| OG0001054 | 7.36 | 1.20±0.09 | $6.269e-44$ | $9.890e-42$ | GGDEF domain-containing protein |
| OG0000721 | 5.73 | 1.19±0.26 | $3.859e-06$ | $1.300e-05$ | sulfite exporter TauE/SafE family protein |
| OG0001909 | 5.51 | 1.19±0.10 | $3.107e-34$ | $1.897e-32$ | hypothetical protein |
| OG0002021 | 4.37 | 1.18±0.15 | $2.166e-15$ | $2.667e-14$ | hypothetical protein |
| OG0001924 | 6.19 | 1.18±0.12 | $7.155e-23$ | $1.830e-21$ | GNAT family N-acetyltransferase |
| OG0001911 | 4.67 | 1.18±0.12 | $1.482e-24$ | $4.452e-23$ | hypothetical protein;polysaccharide deacetylase |
| OG0000463 | 6.56 | 1.18±0.10 | $9.056e-33$ | $4.511e-31$ | type I methionyl aminopeptidase |
| OG0001549 | 5.95 | 1.17±0.16 | $3.269e-13$ | $2.989e-12$ | 3',5'-cyclic-nucleotide phosphodiesterase |
| OG0001442 | 4.82 | 1.16±0.15 | $3.441e-14$ | $3.639e-13$ | hypothetical protein |
| OG0001322 | 7.23 | 1.15±0.18 | $6.422e-11$ | $4.149e-10$ | hypothetical protein;porin |
| OG0000820 | 5.01 | 1.14±0.17 | $3.476e-11$ | $2.317e-10$ | oxidoreductase |
| OG0001913 | 5.76 | 1.14±0.08 | $9.304e-42$ | $1.161e-39$ | acyl-CoA ligase (AMP-forming), exosortase A system-associated |
| OG0002112 | 6.78 | 1.13±0.08 | $9.813e-42$ | $1.161e-39$ | PEP-CTERM system TPR-repeat protein PrsT |
| OG0001329 | 4.99 | 1.12±0.15 | $2.109e-14$ | $2.282e-13$ | hypothetical protein |
| OG0001580 | 4.86 | 1.11±0.14 | $4.938e-15$ | $5.657e-14$ | DeoR family transcriptional regulator;alpha/beta hydrolase |
| OG0000917 | 7.62 | 1.10±0.23 | $2.606e-06$ | $9.120e-06$ | hypothetical protein |
| OG0001138 | 5.62 | 1.10±0.15 | $4.641e-14$ | $4.749e-13$ | Ni/Fe hydrogenase subunit alpha |
| OG0001086 | 4.63 | 1.10±0.14 | $9.861e-15$ | $1.092e-13$ | hypothetical protein |
| OG0001056 | 7.47 | 1.08±0.09 | $5.214e-35$ | $3.525e-33$ | hypothetical protein;ABC transporter substrate-binding protein |
| OG0001552 | 5.30 | 1.08±0.09 | $4.372e-33$ | $2.299e-31$ | hypothetical protein |
| OG0001575 | 6.58 | 1.07±0.18 | $6.499e-09$ | $3.187e-08$ | Crp/Fnr family transcriptional regulator |
| OG0002094 | 5.42 | 1.07±0.14 | $8.715e-14$ | $8.729e-13$ | thiol reductant ABC exporter subunit CydD |
| OG0000403 | 7.42 | 1.06±0.19 | $4.162e-08$ | $1.807e-07$ | RNA polymerase subunit sigma-24;RNA polymerase sigma factor |
| OG0001923 | 5.76 | 1.06±0.10 | $2.872e-28$ | $1.208e-26$ | exosortase A |
| OG0000491 | 6.90 | 1.05±0.11 | $6.184e-23$ | $1.604e-21$ | fructose-bisphosphate aldolase class I |
| OG0000946 | 5.43 | 1.04±0.10 | $2.116e-25$ | $7.029e-24$ | hypothetical protein |
| OG0001663 | 6.96 | 1.04±0.08 | $8.121e-36$ | $5.694e-34$ | phosphomannomutase/phosphoglucomutase |
| OG0002083 | 6.31 | 1.03±0.23 | $1.128e-05$ | $3.422e-05$ | GntP family permease |
| OG0001516 | 4.49 | 1.03±0.20 | $2.311e-07$ | $9.152e-07$ | DksA/TraR family C4-type zinc finger protein |
| OG0001327 | 2.58 | 1.03±0.19 | $3.098e-08$ | $1.370e-07$ | HypC/HybG/HupF family hydrogenase formation chaperone |
| OG0001578 | 5.04 | 1.03±0.19 | $2.811e-08$ | $1.252e-07$ | MarC family protein |
| OG0001344 | 7.16 | 1.03±0.18 | $5.361e-09$ | $2.649e-08$ | hypothetical protein |
| OG0001557 | 4.97 | 1.03±0.09 | $8.103e-30$ | $3.741e-28$ | DUF2063 domain-containing protein |
| OG0001922 | 6.20 | 1.03±0.09 | $7.669e-28$ | $3.156e-26$ | amidotransferase 1, exosortase A system-associated |
| OG0000626 | 3.88 | 1.02±0.43 | $1.686e-02$ | $2.817e-02$ | cupin domain-containing protein |
| OG0001592 | 7.13 | 1.02±0.11 | $1.987e-21$ | $4.477e-20$ | [protein-PII] uridylyltransferase |
| OG0001658 | 6.26 | 1.02±0.11 | $1.852e-20$ | $3.811e-19$ | AMP-dependent synthetase |

| Orthogroup | Mean | $b \pm se$ | $P$ | $P_{adj}$ | Protein product |
|---|---|---|---|---|---|
| OG0001075 | 7.01 | 1.01±0.15 | $6.694e-12$ | $4.940e-11$ | MBL fold metallo-hydrolase |
| OG0000473 | 5.55 | 1.01±0.11 | $6.374e-22$ | $1.527e-20$ | transcriptional regulator |
