# Supplementary material for: Molecular and Physiological Adaptations to Low Temperature in Thioalkalivibrio Strains Isolated from Soda Lakes with Different Temperature Regimes
Source: mSystems. 2021 Apr 27;6(2):e01202-20. doi: 10.1128/mSystems.01202-20 (PMC8092127; doi:10.1128/mSystems.01202-20)
Supplement: TABLE S1 [file msystems.01202-20-st001.pdf]

Sample name	SO_4^{2-} (mM)		$S_2O_3^{2-}$ (mM)	
	Average	Stdev	Average	Stdev
Starting medium 10°C	nd	nd	17.7	0.2
Starting medium 30°C	nd	nd	20.0	1.0
AL2^T 10°C (1)	35.0	0.4	nd	nd
AL2^T 10°C (2)	36.5	0.5	nd	nd
AL2^T 10°C (3)	36.2	0.1	nd	nd
AL2^T 10°C (4)	35.8	0.2	nd	nd
AL2^T 30°C (1)	30.4	0.4	nd	nd
AL2^T 30°C (2)	37.7	0.4	nd	nd
AL2^T 30°C (3)	43.0	0.3	nd	nd
AL2^T 30°C (4)	44.2	0.2	nd	nd
ALJ2 10°C (1)	43.0	0.1	nd	nd
ALJ2 10°C (2)	35.0	0.2	nd	nd
ALJ2 10°C (3)	36.7	0.4	nd	nd
ALJ2 10°C (4)	39.6	0.3	nd	nd
ALJ2 30°C (1)	31.3	0.2	nd	nd
ALJ2 30°C (2)	34.5	0.4	nd	nd
ALJ2 30°C (3)	42.2	0.3	nd	nd
ALJ2 30°C (4)	33.7	0.0	nd	nd
