# Supplementary material for: Molecular and Physiological Adaptations to Low Temperature in Thioalkalivibrio Strains Isolated from Soda Lakes with Different Temperature Regimes
Source: mSystems. 2021 Apr 27;6(2):e01202-20. doi: 10.1128/mSystems.01202-20 (PMC8092127; doi:10.1128/mSystems.01202-20)
Supplement: TABLE S3 [file msystems.01202-20-st003.pdf]

Sample	Strain	Temp (°C)	Total reads	Pseudoaligned reads	% mapped	N° rRNA reads	N° tmRNA reads	N° ncRNA reads
S01	AL2(T)	10	3041992	2341726	76.98	19669	180329	45782
S02	AL2(T)	10	4083034	3118155	76.37	27718	276422	72389
S03	ALJ2	10	4884896	3653785	74.80	25237	190679	203821
S04	ALJ2	10	5625169	4286415	76.20	28170	226555	223780
S05	AL2(T)	10	5869266	4518344	76.98	39516	436287	98472
S06	AL2(T)	10	5274711	3992751	75.70	64000	402219	111745
S07	ALJ2	10	6462477	4834602	74.81	78979	303286	226747
S08	ALJ2	10	4923006	3565523	72.43	47766	293644	319909
S09	AL2(T)	30	3935993	2907617	73.87	26580	453203	324315
S10	AL2(T)	30	3534185	2772811	78.46	28963	427135	241429
S11	ALJ2	30	3123251	2486380	79.61	21308	345161	195907
S12	ALJ2	30	3291330	2638107	80.15	27079	357614	180922
S13	AL2(T)	30	4464535	3365945	75.39	26201	606295	352919
S14	AL2(T)	30	4031932	3101853	76.93	18518	539855	300297
S15	ALJ2	30	4053456	3115039	76.85	9709	534875	296195
S16	ALJ2	30	3604888	2797966	77.62	4954	340246	266938
